# Design, Synthesis and In Vitro Investigation of Cabozantinib-Based PROTACs to Target c-Met Kinase

**DOI:** 10.3390/pharmaceutics14122829

**Published:** 2022-12-16

**Authors:** Anastasia A. Sachkova, Daria V. Andreeva, Alexander S. Tikhomirov, Alexander M. Scherbakov, Diana I. Salnikova, Danila V. Sorokin, Fedor B. Bogdanov, Yulia D. Rysina, Andrey E. Shchekotikhin, Ekaterina S. Shchegravina, Alexey Yu. Fedorov

**Affiliations:** 1 Department of Organic Chemistry, Nizhny Novgorod State University, Gagarina Av. 23, 603950 Nizhny Novgorod, Russia; 2 Gause Institute of New Antibiotics, 11 B. Pirogovskaya Street, 119021 Moscow, Russia; 3 Department of Experimental Tumor Biology, Blokhin N.N. National Medical Research Center of Oncology, 115522 Moscow, Russia; 4 Faculty of Fundamental Medicine, Moscow State University, 119991 Moscow, Russia

**Keywords:** c-Met, PROTAC, cabozantinib, cancer

## Abstract

(1) Background: This investigation aimed at developing a series of c-Met-targeting cabozantinib-based PROTACs. (2) Methods: Purification of intermediate and target compounds was performed using column chromatography, in vitro antiproliferation activity was measured using a standard MTT assay and a c-Met degradation assay was performed via the immunoblotting technique. (3) Results: Several compounds exhibited antiproliferative activity towards different cell lines of breast cancer (T47D, MDA-MB-231, SKBR3, HCC1954 and MCF7) at the same level as parent cabozantinib and 7-demethyl cabozantinib. Two target conjugates, bearing a VHL-ligand as an E3-ligase binding moiety and glycol-based linkers, exhibited the effective inhibition of c-Met phosphorylation and an ability to decrease the level of c-Met in HCC1954 cells at micromolar concentrations. (4) Conclusions: Two compounds exhibit c-Met inhibition activity in the nanomolar range and can be considered as PROTAC molecules due to their ability to decrease the total level of c-Met in HCC1954 cells. The structures of the offered compounds can be used as starting points for further evaluation of cabozantinib-based PROTACs.

## 1. Introduction

PROTAC technology has exhibited impressive progress [1] since the first PROTAC molecule was designed in 2001 by C.M. Crews [2] to the point where several candidates entered clinical trials in 2019–2021 [3]. In general, the PROTAC molecule consists of an L_P_-ligand that connects to the target protein of interest (POI), associated through the linker with L_E_-ligand, recruiting E3-ligase. Attractive features of this technology are the ability of PROTAC degraders to target proteins without distinct active sites (i.e., “undruggable” proteins) and their catalytic mode of action [4].

The range of targets degraded by PROTAC is constantly growing [5]. Currently, it includes protein kinases [6,7,8] (AKT, BCR-ABL, BTK etc.), epigenetic regulators (BRD4/7/9 [9,10,11,12], TRIM24 [13], Smad3 [14], proteins related to neurodegenerative diseases (Tau [15], mutant huntingtin [16], PSD-95 [17], α-synuclein [18]), nuclear receptors (ERRα [19], AR [20], PARP1 [21], RAR [22]) and anti-apoptotic proteins [23,24,25] (MCL-1, BCL-2, BCL-XL).

Auxiliary proteins named E3 ligases are essential for the ubiquitination of POI. To date, only a few examples (VHL [11,13,19,26], CRBN [9,22,27], MDM2 [10,21,28], IAPs [29,30]) have been confirmed for PROTAC design despite the discovery of more than 700 E3 ligases in humans [31]. Proteins such as VHL can serve not only as E3 ligases but also as POIs [32] due to their ability to interact with HIF1α [33] and facilitate their proteasomal degradation. Based on this consideration, VHL was chosen for the developing of the co-called “homoPROTAC” [32,34]. Nevertheless, expanding the range of potent E3-ligases still remains important for PROTACs’ evolution [35,36,37].

One of the key steps in protein degradation via the ubiquitin–proteasome system (UPS) is the transfer of the polyubiquitin chain to the POI. The linker between L_P_ and L_E_ plays an essential role in this process. All factors: length, flexibility, polarity and presence of functional groups can influence transfer efficiency [38,39,40,41]. The most common motifs for linker design are PEG- or other glycol-based spacers, triazole-, piperidine- and piperazine-bearing fragments. Meanwhile, the choice of linker structure is often carried out empirically by screening an activity of a series of PROTAC molecules.

Despite the fundamental principles of PROTAC action being sufficiently understood, some aspects of PROTAC technology remain unclear. The main issues are related to identifying new possible targets for UPS degradation and to extend the range of E3-ligases. Furthermore, evaluation of PROTACs’ pharmacokinetic and pharmacodynamic characteristics, indicating side targets and determining selectivity, is not trivial. More efforts are needed to fully understand the complete mechanisms of action and utilization of PROTACs [42].

This work is focused on the design and synthesis of cabozantinib-based PROTACs, recruiting CRBN and VHL as E3-ligases and the investigation of their efficiency depending on the linker structure. The choice of cabozantinib as a warhead stemmed from its excellent activity against hepatocellular endothelial growth factor receptor (HEGFR or c-Met), endothelial growth factor receptor (EGFR) and other tumor-associated proteins KIT, RET, AXL, TIE-2 and FLT-3 [43,44]. In addition to the abnormal activation of c-Met along with the progression of glioblastoma, liver [45], colon [46] and pancreatic [47] carcinomas, the selection of c-Met as a target protein for degradation is due to its deep involvement in the activation of several downstream signaling pathways (MAPK, PI3K, SRC, STAT) [48] that play an essential role in cell proliferation and survival.

Cabozantinib itself has been approved by the FDA for progressive metastatic medullary thyroid cancer and advanced renal cell carcinoma [49,50]. However, a wide range of dose-dependent side effects of cabozantinib have occurred in many cases [50,51,52]. Application of cabozantinib-based PROTAC is believed to enhance antitumor action and reduce adverse effects due to the PROTAC catalytic mode of action that could significantly decrease the required drug dose.

## 2. Materials and Methods

### 2.1. Chemistry

Agilent DDR2 400 and Varian Mercury 400 Plus spectrometers were used to record the 1H NMR and 13C NMR spectra at 25 °C. The ppm measurement unit was applied for the report of the chemical shifts (δ) in relation to the solution of the compound in DMSO-d6 and CDCl_3_, while the J values and the internal reference TMS were expressed in Hertz. Only the NMR assignment received the atomic numeration. The Bruker Microflex LT spectrometer was used to record the MALDI spectra. The electron spray ionization (ESI) was applied for the high-resolution mass spectra record on a Bruker Daltonics microOTOF-QII instrument. The Elementar (Vario Micro Cube) apparatus was a tool for the elemental analysis. The Shimadzu Class-VP V6.12SP1 system was used for the HPLC, A: 0.01 M H_3_PO_4_ pH 2.6; B: MeCN; all the components were characterized to have purity more than 95%. The Merck Kieselgel 60 (70–230 mesh) was applied for the column chromatography. Commercially available reagents were acquired to perform all the reactions. The purification of the solvents was performed in compliance with the standard procedures. The purchased petroleum ether is appropriate for the fraction of 40–70 °C. Syntheses of VHL-ligand 10 [53], its derivatives 10a,b [54] and conjugates of lenalidomide with caproic acid in forms of N-Boc protected (12a) and free amine (12b) [36], cabozantinib hydroxyl derivative 3 [54] were performed as described earlier.

### 2.2. Biological Research

#### 2.2.1. Cell Culture Screening and Assessment of the Antiproliferative Effects

The A-431 (skin), A549 (lung), HeLa (cervical), T47D, MDA-MB-231, SKBR3, HCC1954 and MCF7 (breast) cancer cells were acquired from the ATCC center and incubated in normal media (Gibco, New York, NY, USA) recommended by the manufacturer. The MTT (3-[4,5-dimethylthiazol-2-yl]-2,5-diphenyltetrazolium bromide) (Applichem) assay with modifications [55] was used to assess the cell growth as described earlier in [56]. The seeding of the tumor cells was performed in 24-well plates (TPP) using 900 μL of the appropriate medium. DMSO (AppliChem, Darmstadt, Germany) was applied for the compound dissolution to a concentration of 10 mM immediately before the experiments. Subsequently, the obtained solutions were diluted in the DMEM medium to the necessary doses. The solutions of the investigated compounds with various concentrations in 100 μL of the DMEM were added a day following the seeding, and the cell cultures were incubated for 3 days. After the cell growth with the compounds, the DMEM was ablated, and the MTT dye as part of the medium was added to the end concentration of 0.2 mg/mL to every well for the incubation during 2 h. All the wells were supplemented with DMSO in the amount of 350 μL for solvation of the MTT formazan purple crystals. A MultiScan reader (ThermoFisher, Waltham, MA, USA) was applied for the measurement of the solutions’ absorbance at a wavelength of 571 nanometers. The cell survival was evaluated after subtraction of the blank value (the absorbance in each well without cells) from all wells. All of the tests were performed 3 times. The investigation of the dose–response curves was performed with the usage of regression analysis and sigmoidal curves (Log(concentration) vs. normalized absorbance). GraphPad Prism was applied for the establishment of the half-maximal inhibitory concentrations (IC_50_) (Table 1).

#### 2.2.2. Immunoblotting

To prepare samples for immunoblotting analysis, the cells were seeded on 100 mm dishes (Corning, New York, NY, USA). For experiments with HCC1954 breast cancer cells, the compounds **15a** and **15c** were added in fresh medium after 24 h of seeding. For the cell extracts’ preparation, cell cultures were washed in phosphate-buffered saline (PBS) 2 times and incubated for 10 min on ice in the lysis buffer. The aforementioned buffer had a pH of 7.5 and contained Tris-HCl (50 mM), NaCl (150 mM), Igepal CA-630 (0.5%), DTT (1 mM), EDTA (1 mM), sodium orthovanadate (0.1 mM), PMSF (1 mM) and pepstatin, leupeptin, aprotinin (1 µg/mL each) as described earlier [57]. The Bradford method [58] was used to define the level of the protein. The cell extracts were divided in SDS-PAGE (10%) at reducing conditions, transmitted to a nitrocellulose membrane (GE HealthCare, Chicago, IL, USA) and treated following a regular methodology. To prevent nonspecific absorption, the membranes were processed with 5% nonfat milk solution in TBS buffer at a pH of 7.5. The aforesaid buffer contained Tris (20 mM), NaCl (500 mM) and Tween-20 (0.1%). Thereafter, the membranes were incubated with primary antibodies during the night at a temperature of 4 °C. c-Met and phospho-c-Met (Tyr1234/1235, Tyr1003) antibodies were acquired from Cell Signaling Technology; the antibodies versus α-tubulin (Cell Signaling Technology, Danvers, MA, USA) were applied for a loading control. Goat anti-rabbit IgGs (Jackson ImmunoResearch, West Grove, PA, USA) associated with horseradish peroxidase were involved in immunoblotting in the quality of secondary antibodies. The detection of the signals was performed with the usage of the ECL reagent as characterized by Mruk and Cheng [59] and an ImageQuant LAS4000 system (GE HealthCare, Chicago, IL, USA). Densitometry for the tested proteins/α-tubulin ratio was carried out using ImageJ software.

## 3. Results and Discussion

### 3.1. Chemistry

**Figure 1:**
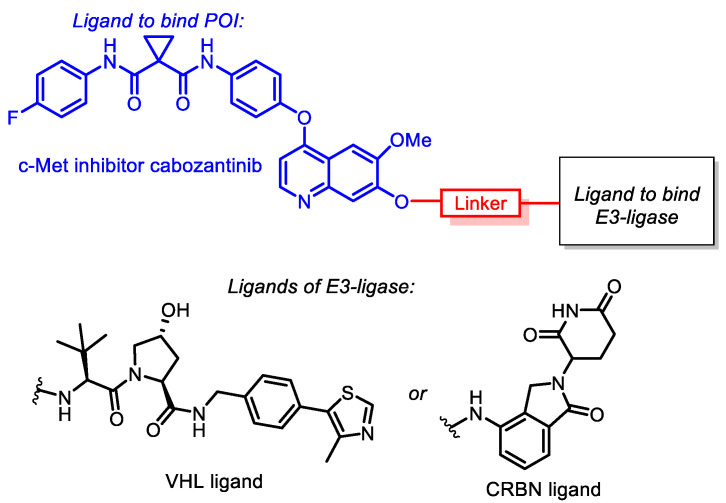
General structures of cabozantinib-based PROTACs.

All synthesized conjugates included cabozantinib as a warhead, whereas CRBN-ligand lenalidomide (an analogue of thalidomide, a derivative of naturally occurring glutamic acid) or VHL-ligand (S,R,S)-AHPC were exploited as E3-ligase ligands. The mentioned molecules were joined with linkers of various lengths and structures. Simple aliphatic spacers, glycol-based spacers and linkers bearing a triazole core were applied in conjugate synthesis. The general structures of target conjugates are represented in Figure 1.

Since the binding affinity of cabozantinib after conjugation should remain at the same level as for the intact molecule, it was important to determine the possible conjugation site. Previously [60], it was reported that structural analogues of cabozantinib functionalized on the position “7” of the quinazoline core demonstrated almost the same activity as parent compounds towards c-Met.

**Scheme 1:**
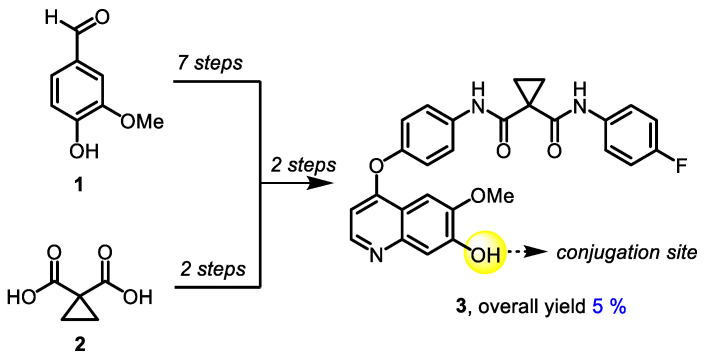
Synthesis of 7-demethylated cabozantinib **3**.

The synthetic approach to 7-demethylated cabozantinib congener **3**, a common building block for all target molecules, was based on a previously published protocol [61] and included 11 steps. 4-Hydroxy-3-methoxybenzaldehyde (**1**) and cyclopropane-1,1-dicarboxylic acid (**2**) were used as starting materials (Scheme 1).

**Scheme 2:**
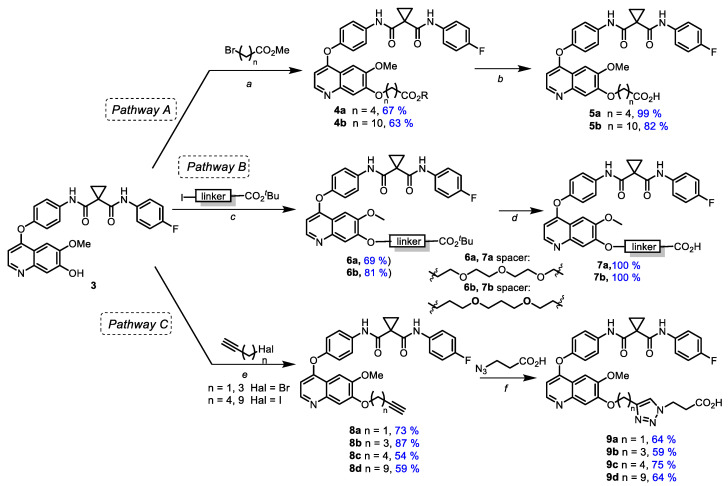
Synthesis of ω-carboxyl bearing cabozantinib derivatives. Reagents and conditions: (**a**) NaH, DMF, 0 °C → 65 °C; (**b**) NaOH, MeOH-H_2_O (2:1), 60 °C; (**c**) Cs_2_CO_3_, DMF, 65 °C; (**d**) TFA, rt; (**e**) Cs_2_CO_3_, DMF, rt; (**f**) TBTA, CuSO_4_*5H_2_O, AscNa, DMF-H_2_O (1:1), 55 °C.

Starting from 7-demethylated cabozantinib analogue **3**, a series of carboxyl-containing derivatives were synthesized via two approaches. The first route (Scheme 2, pathway A) was based on the alkylation of **3** with esters of halogenated carboxylic acids with the subsequent hydrolysis of the ester group (Scheme 2). Various bases (Cs_2_CO_3_, K_2_CO_3_, Na_2_CO_3_, DBU, NaH), solvents (1,4-dioxane, DMF, THF, toluene, acetone) and temperatures were applied, and the highest yields were achieved using NaH in DMF at 65 °C. The treatment of **3** with methyl esters of 5-bromovaleric and 11-bromoundecanoic acids in the presence of NaH in DMF resulted in 7-O-alkylated cabozantinib derivatives **4a**,**b** (67% and 63%, respectively). When methyl 2-bromoacetate and methyl 3-bromopropionate were used for the alkylation of **3**, the yields of isolated products dramatically dropped; therefore only two derivatives were entered in the next step. Cleavage of the ester group in **4a**,**b** by alkaline treatment led to appropriate acids **5a**,**b** in good to high yields. A similar synthetic approach (Scheme 2, pathway B) was applied to obtain carboxylic derivatives containing ethylene glycol (**7a**) as well as a propane-1,3-diol-based linker (**7b**).

Another pathway (Scheme 2, pathway C) included the alkylation of **3** with ω-haloalkynes containing a terminal triple bond and subsequent azide-alkyne cycloaddition with 3-azidopropionic acid followed by the formation of carboxylic cabozantinib derivatives **9a**–**d** containing triazole linkers. Alkynes with various meanings of the chain length were used to define the impact of triazole moiety location on PROTACs’ activity (Scheme 2).

**Scheme 3:**
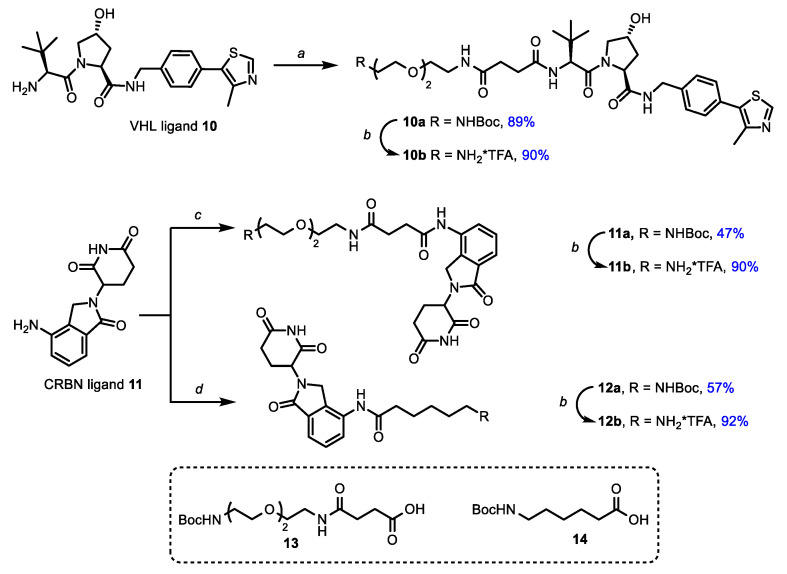
Synthesis of ligands for E3-ligases. Reagents and conditions: (**a**) **13**, DIPEA, HATU, DCM, rt; (**b**) TFA, DCM, rt; (**c**) **13**, HATU, DIPEA, DMF, rt; (**d**) **14**, HATU, DIPEA, DMF, rt.

Next, synthetic blocks, containing moieties-targeted E3-ligases CRBN and VHL, were synthesized (Scheme 3). For this purpose peptidomimetic ligand **10** for VHL, obtained by previously published methods, and commercially available lenalidomide (**11**) for CRBN [53,62] were treated with ω-*N*-Boc protected carboxylic acids [36,54] **13** and **14** (Scheme 3) that led to protected amines **10a**, **11a** and **12a** with good to high yields.

**Figure 2:**
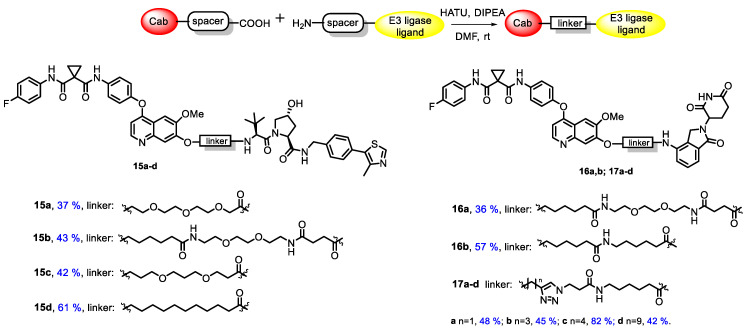
Synthesis and structures of target conjugates.

At the next step, protecting groups were removed by a trifluoroacetic acid treatment and corresponding amines **10b**, **11b** and **12b** were coupled with carboxylic cabozantinib derivatives **5a**,**b**, **7a**,**b** and **9a**–**d** to obtain target PROTACs **15a**–**d**, **16a**,**b** and **17a**–**d** (Figure 2). The final step was carried out under a standard peptide coupling procedure with HATU in the presence of DIPEA in DMF (Figure 2).

### 3.2. Biology

#### 3.2.1. Cancer Cell Line Screening and Antiproliferative Evaluation

**Figure 3:**
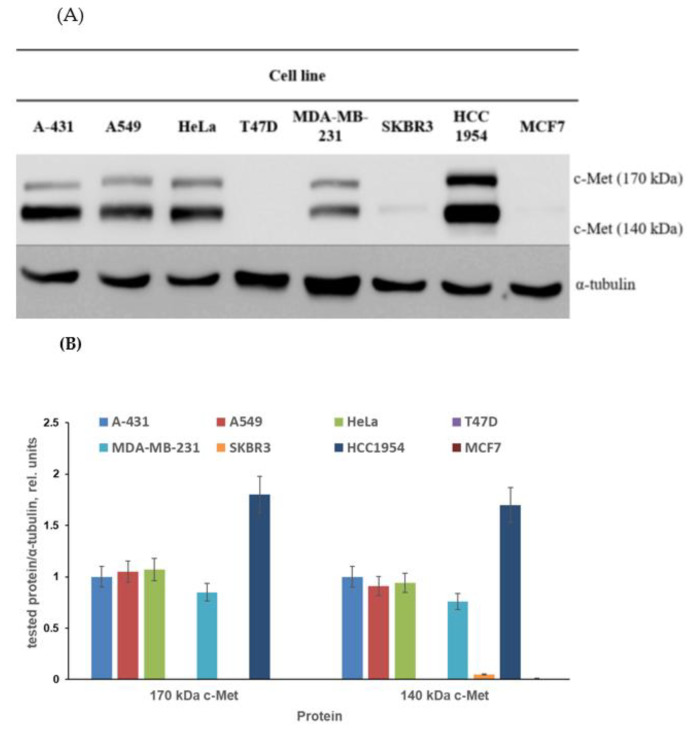
The c-Met expression level in selected cell lines. (**A**) Representative immunoblotting images; (**B**) densitometry for immunoblotting data (*n* = 3) was carried out using ImageJ software (Wayne Rasband, NIH) with the protocol provided by The University of Queensland and the recommendations from the work [69].

The first aim of the biological part of the study was to find the proper cell model for testing of obtained conjugates. The published data on c-Met expression in cancer cell lines are quite contradictory. The c-Met expression described in several papers varies considerably. The levels of c-Met expression depend on the method of analysis and characteristics of antibodies used by the researchers. Moreover, sample preparation techniques can affect the identified c-Met expression level [63,64,65,66,67,68]. Considering the above, we screened cancer cell lines to check the level of c-Met expression. The different cell lines of breast cancer (T47D, MDA-MB-231, SKBR3, HCC1954 and MCF7), skin cancer (A-431), lung cancer (A549) and cervical cancer (HeLa) were cultured without obtained compounds and then subjected to immunoblotting with antibodies against c-Met, as characterized in the Materials and Methods section. As demonstrated in Figure 3, the c-Met expression level varied considerably between cells. First, no c-Met expression was observed in MCF7, SKBR3 and T47D breast cancer cells. A rather low c-Met level was revealed in MDA-MB-231 breast tumor cell cultures; nevertheless, 140 and 170 kDa c-Met proteins were clearly visible in these cells, as can be seen in Figure 3.

The c-Met expression level evaluated in A-431, A549 and HeLa cells was moderate. Finally, the highest level of c-Met was detected in HCC1954 breast cancer cells. Thus, for further experiments, we used cell lines with different levels of c-Met expression (Table 1).

The antiproliferative activity of target compounds against selected cancer cell lines is shown in Table 1. Compounds **17a**–**d** bearing a triazole ring in the spacer between cabozantinib (L_P_) and lenalidomide (L_E_) demonstrated low activity towards all tested cell lines. The length of the spacer and position of triazole did not play any role in antiproliferative activity. Derivatives **16a**,**b**, in which L_P_ and L_E_ were bound by using a combination of ethylene or propylene glycol fragments, were slightly superior to triazole-based analogues **17a**–**d**; however, they had low antiproliferative potency towards HCC1954 cells. In contrast, conjugates **15a**–**c** were found to exhibit the most promising activity, especially towards the HCC1954 cell line with the highest c-Met expression. It may be supposed that the introduction of VHL as the L_E_-end of PROTAC conjugates can generally enhance the antitumor potency of compounds. However, compound **15d**, bearing an aliphatic nonpolar linker, was less potent in comparison with other compounds series **15** as well as with reference molecules **3** and cabozantinib. The high activity of cabozantinib in T47D cancer cells draws attention. Interestingly, these cells do not express known cabozantinib targets, including c-Met. The cabozantinib activity against T47D cells described in our work coincides with that described in [70]. We believe that the search for new targets of cabozantinib in c-Met/VEGFRs/KIT-negative cells is a promising task. Mimicking the activity of cabozantinib, compounds **3**, **15a**, and **16a**,**b** were also active against c-Met-negative T47D breast cancer cells.

#### 3.2.2. Assessment of c-Met Inhibition

**Figure 4:**
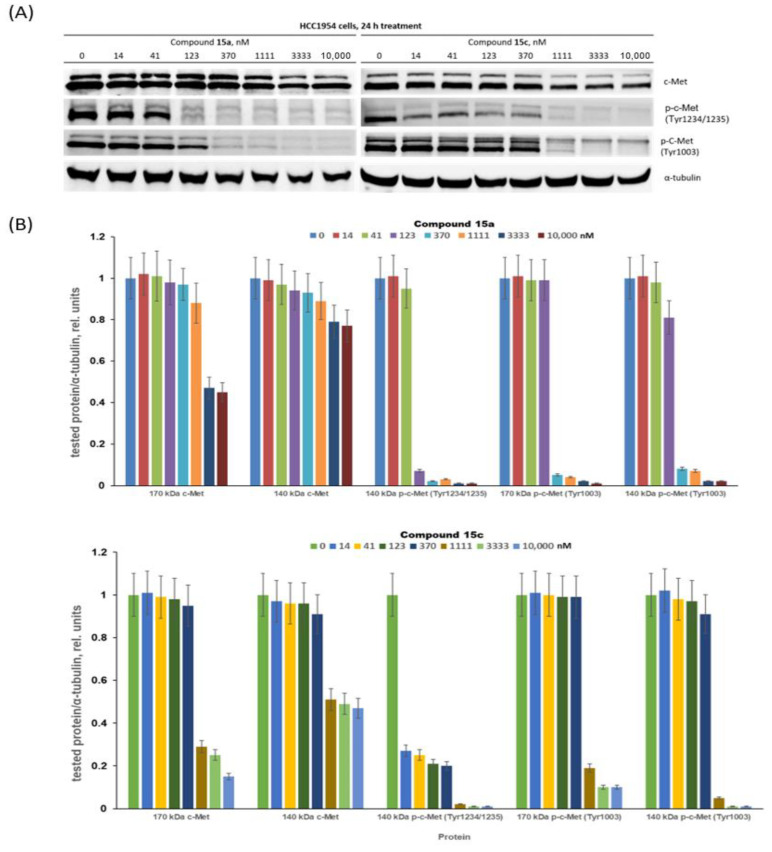
Decreasing c-Met phosphorylation and expression with conjugates **15a** and **15c**. (**A**) Representative immunoblotting images; (**B**) densitometry for immunoblotting data (*n* = 3) was carried out using ImageJ software (Wayne Rasband, NIH) with the protocol provided by The University of Queensland and the recommendations from the work [69].

Compounds **15a** and **15c** significantly inhibited the growth of c-Met-positive HCC1954 cells and had notably lower antiproliferative effects on the c-Met-negative MCF7 cells. These compounds were chosen for in-depth evaluation as c-Met targeting agents. HCC1954 cells were treated with compounds **15a** and **15c** at concentrations ranging from 14 to 10,000 nM, and then the levels of c-Met protein and its phosphorylated forms were determined by immunoblotting (Figure 4).

First, we proved that the cabozantinib part of the PROTAC molecules **15a** and **15c** can still interact with the c-Met kinase. The c-Met intracellular sequence consists of a juxtamembrane domain, a tyrosine kinase domain and a C-terminal multifunctional docking site [71,72]. The juxtamembrane domain contains amino acid residues including Tyr1003, which interacts with casitas B-lineage lymphoma (c-Cbl) and leads to ubiquitin-dependent c-Met degradation [73,74]. This signalling process is a mechanism of a negative regulatory loop, which modulates the c-Met activation status. Pascal Peschard and colleagues found that mutation of the c-Cbl tyrosine kinase binding domain on the c-Met receptor converts it into a transforming protein [73]. The authors replaced the juxtamembrane tyrosine of c-Met by phenylalanine. The mutant c-Met had transforming activity in fibroblast and epithelial cells. The tyrosine kinase c-Met domain, upon phosphorylation of Tyr1234 and Tyr1235, undergoes a conformational change resulting in increased kinase activity. Although both compounds demonstrate a similar effect on the Tyr1003 phosphorylation residue at juxtamembrane c-Met domain, it appears at 123 nM and above (Figure 4), conjugate **15c** can block phosphorylation Tyr1234/1235 (belonging to tyrosine kinase c-Met domain) already at 14 nM, whereas similar effects for **15a** were observed at concentrations higher than 100 nM. We believe that the simultaneous blocking of tyrosine phosphorylation in two receptor domains is a very important characteristic of the lead PROTAC molecules. Treatment of HCC1954 cells with both conjugates **15a** and **15c** resulted in a decrease in the levels of c-Met in a dose-dependent manner. Figure 4 shows that compounds **15a** and **15c** at nanomolar concentrations had a weak effect on the level of c-Met, while low micromolar concentrations led to near complete blocking of the accumulation of p-c-Met in HCC1954 breast cancer cells. Altogether, PROTAC conjugates **15a** and **15c**, exhibiting antiproliferative effects and suppressing c-Met kinase at low concentrations, are promising leaders for further development.

## 4. Conclusions

We characterized the design, synthesis and biological assessment of a series of cabozantinib-based PROTACs, recruiting CRBN and VHL as E3-ligases. The obtained compounds exhibited antiproliferative effects against a number of tumor cell lines. Compounds **17a**–**d**, bearing a triazole linker, demonstrated low antiproliferative activity towards all tested cell lines. No correlation was observed between the distance of triazole and cabozantinib fragments and antiproliferative activity. Compounds **15a**–**d** demonstrated more pronounced antiproliferative activity towards the panel of selected cell lines. However, compound **15d**, bearing a nonpolar linker with 11 atoms as well as compound **15b** with a polar but longer spacer, were less active than compounds with glycol- and propanediol-based linkers **15a** and **15c**. They caused a 50% suppression of HCC1954 cell proliferation at a dose of approximately 6 μM and 7 μM, respectively, meaning they provide a new perspective for further studies considering c-Met as the main target of the synthesized conjugates. Both compounds at nanomolar concentrations reduced the level of the phosphorylated form of c-Met protein and were able to block the c-Met accumulation in tumor cells at higher concentrations, indicating the high potency of novel anticancer cabozantinib-based PROTACs. Further research may be aimed at the optimization of the PROTAC linker structure and the search for other PROTAC targets in the cancer cells (MET, RET, KIT, FLT1, FLT3, FLT4, TIE2, AXL, etc.). The obtained data clearly indicate the complex relationship between the biological activity of the conjugate and the length, flexibility and polarity of the linker between L_P_ and L_E_. Apparently, the glycol-based linkers, bearing 10–12 atoms, are the most suitable in the case of the described molecules. Moreover, the mechanisms of action of the leader conjugates, and particularly an assessment of the antitumor efficacy of these agents in vivo, will be established.

## Figures and Tables

**Table 1:** Antiproliferative activity of target compounds **15a**–**d**, **16a**,**b**, **17a**–**d** against cancer cells (IC_50_ values ^1^, µM); the dose–response curves are presented in Figures S1–S5.

	Cell Line				
Compound	A-431	T47D	MCF7	HCC1954	SKBR3
c-Met Status	++	-	-	+++	-
**15a**	>25	6.1	28.1	5.7	>25
**15b**	>25	14.2	>25	9.1	>25
**15c**	>25	>25	>50	6.7	10.8
**15d**	>25	>25	>50	20.3	>25
**16a**	12.3	8.2	19.4	>25	>25
**16b**	>25	3.8	>25	>25	>25
**17a**	>25	>25	>25	>25	>25
**17b**	>25	>25	>25	>25	>25
**17c**	>25	>25	>25	>25	>25
**17d**	>25	>25	>25	>25	>25
**3**	20.6	7.2	14.8	9.8	22.6
**c** **abozantinib**	11.2	4.4	9.0	8.7	18.1

^1^ Cells were incubated with compounds for 72 h; data are shown as the averages of three experiments. Standard deviations were within 10% and not demonstrated for the simpler reading of the results. “-”, “++” and “+++” indicates the level of c-Met expression: almost no c-Met, average c-Met expression and high level of c-Met expression, respectively

## Data Availability

Not applicable.

## Supplementary Materials

The following supporting data can be downloaded at https://www.mdpi.com/article/10.3390/pharmaceutics14122829/s1: synthetic procedures, description and copies of NMR spectra, and dose–response curves. Figure S1. Antiproliferative activity of target compounds **15a**–**d**, **16a**,**b**, **17a**–**d** against A-431 cells; the A-431 cells were incubated with compounds for 3 days and the cell viability was assessed by the MTT test. Figure S2. Antiproliferative activity of target compounds **15a**–**d**, **16a**,**b**, **17a**–**d** against T47D cells. Figure S3. Antiproliferative activity of target compounds **15a**–**d**, **16a**,**b**, **17a**–**d** against MCF7 cells. Figure S4. Antiproliferative activity of target compounds **15a**–**d**, **16a**,**b**, **17a**–**d** against HCC1954 cells. Figure S5. Antiproliferative activity of target compounds **15a**–**d**, **16a**,**b**, **17a**–**d** against SKBR3 cells. Reference [75] is cited in the supplementary materials.
