# Supplementary material for: Design, Synthesis and In Vitro Investigation of Cabozantinib-Based PROTACs to Target c-Met Kinase
Source: Pharmaceutics. 2022 Dec 16;14(12):2829. doi: 10.3390/pharmaceutics14122829 (PMC9781691; doi:10.3390/pharmaceutics14122829)
Supplement: Supplementary file 1 [file pharmaceutics-14-02829-s001.zip › pharmaceutics-2052913-supplementary.pdf]

# Supporting information for:

# Design, synthesis and *in vitro* investigation of cabozantinib-based PROTACs to target c-Met kinase

*Anastasia A. Sachkova[1], Daria V. Andreeva[2], Alexander S. Tikhomirov[2], Alexander M. Scherbakov[3], Diana I. Salnikova[3], Danila V. Sorokin[3], Fedor B. Bogdanov[3,4], Andrey E. Shchekotikhin[2], Yulia D. Rysina[1], Ekaterina S. Shchegravina[1*], Alexey Yu. Fedorov[1*]*

[1] *Department of Organic Chemistry, Nizhny Novgorod State University, Gagarina av. 23, Nizhny Novgorod 603950, Russian Federation*
[2] *Gause Institute of New Antibiotics, 11 B. Pirogovskaya Street, Moscow 119021, Russian Federation*
[3] *Department of Experimental Tumor Biology, Blokhin N.N. National Medical Research Center of Oncology, 115522 Moscow, Russia*
[4] *Faculty of Fundamental Medicine, Moscow State University, 119991 Moscow, Russia*
*corresponding authors: sc.katarina@yandex.ru (E.S.S.), afedorovnn@yandex.ru (A.Yu.F)

## Table of contents

Synthetic procedures for intermediate compounds and target conjugates ........ 3
Synthesis of cabozantinib-based esters with aliphatic linker 4a, 4b........ 3
Synthesis of cabozantinib-based acids with aliphatic linker 5a, 5b ........ 4
Synthesis of cabozantinib-based esters with polyethylene- and polypropylene glycol linkers 6a, 6b ........ 6
Synthesis of cabozantinib-based acids with polyethylene- and polypropylene glycol linkers 7a, 7b ........ 7
Synthesis of alkynyl cabozantinib derivatives 8a-d ........ 8
Synthesis of cabozantinib-based acids with triazole linker 9a-d........ 10
Synthesis of cabozantinib-based PROTACs 15a-d, 16a, b, 17a-d ........ 14
$^{1}$H and $^{13}$C NMR spectra ........ 23
Antiproliferative activity of target compounds ........ 51
References ........ 54

## Synthetic procedures for intermediate compounds and target conjugates

## General information

$^{1}$H NMR and $^{13}$C NMR spectra were recorded on Agilent DDR2 400 spectrometer or Varian Mercury 400 Plus spectrometer at 25 °C. Chemical shifts (δ) are reported in ppm for the solution of compound in $CDCl_3$ and DMSO-$d_6$ with internal reference TMS and *J* values in Hertz. Atomic numeration is given only for NMR assignment. MALDI spectra were recorded on Bruker Microflex LT spectrometer. High resolution mass spectra were recorded with electron spray ionization (ESI) on a Bruker Daltonics microOTOF-QII instrument. Elemental analysis was performed using an Elementar (Vario Micro Cube) apparatus, HPLC was performed using a Shimadzu Class-VP V6.12SP1 system, A: 0.01 M $H_3PO_4$ pH 2.6; B: MeCN, all the compounds were found to have purity >95%. Column chromatography was performed using *Merck Kieselgel 60* (70 – 230 mesh). All reactions were performed with commercially available reagents. Solvents were purified according to standard procedures. The petroleum ether used corresponds to fraction 40 – 70 °C.

Syntheses of VHL-ligand **10** [1], its` derivatives **10a-b** [2], and conjugates of lenalidomide with carpoic acid in forms of *N*-Boc protected (**12a**) and free amine (**12b**) [4], cabozantinib hydroxyl derivative **3** [5] were performed as described earlier.

## Synthesis of cabozantinib-based esters with aliphatic linker 4a, 4b

### General procedure for synthesis of esters 4a, 4b

A suspension of NaH in mineral oil (3.00 equiv.) in THF (3 mL) was added dropwise to the ice-cold solution of cabozantinib derivative **3** (1.00 equiv.) in THF (3 mL) in inert atmosphere. Reaction mixture was stirred at 0°C for 1 h and the solution of corresponding halogenated acid ester (2.00 equiv.) in THF (2 mL) was added dropwise. The resulting solution was stirred at 65° C for 2 h. The solvent was removed under reduced pressure and the residue was purified using column chromatography to give the corresponding ethers.

**Methyl 5-((4-(4-(1-((4-fluorophenyl)carbamoyl)cyclopropane-1-carboxamido)phenoxy)-6-methoxyquinolin-7-yl)oxy)pentanoate 4a**

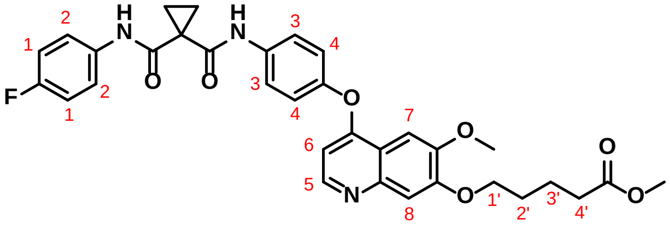

Eluent: ethyl acetate-methanol (1:0 → 9:1). Pale-yellow solid, yield 67%.
**M.p.** = 110 – 111 °C.
**$^{1}$H NMR** (400 MHz, DMSO-$d_6$): δ 10.18 (s, *NH*, 1H), 10.05 (s, *NH*, 1H), 8.46 (d, *J* = 5.2 Hz, *C5-H*, 1H), 7.76 (d, *J* = 8.8 Hz, *C3-H*, 2H), 7.67-7.61 (m, *C2-H*, 2H), 7 .50 (s, *C7-H*, 1H), 7 .38 (s, *C8 -H*, 1H), 7.22 (d, *J* = 8.9 Hz, *C4-H*, 2H), 7.19-7.12 (m, *C1-H*, 2H), 6.42 (d, *J* = 5.2 Hz, *C6 -H* ,1H), 4.15 (t, *J* = 6.1 Hz, *C1'-H*, 2H), 3.93 (s, *OMe*, 3H), 3.60 (s, *COOMe*, 3H), 2.43 (t, *J* = 7.3 Hz, *C4'-H*, 2H), 1.89 – 1.79 (m, *C2'-H*, 2H), 1.79 – 1.68 (m, *C3'-H*, 2H), 1.48 (s, $C_{cyclopropane}$-*H*, 4H).

**$^{13}$C NMR** (101 MHz, DMSO-$d_6$): δ 173.27, 168.16, 168.11, 158.27 (d, *J* = 240.1 Hz), 159.92, 151.81, 149.50, 149.41, 148.77, 146.44, 136.37, 135.16 (d, *J* = 2.6 Hz), 122.41 (d, *J* = 7.9 Hz, 2C), 122.16 (2C), 121.13 (2C), 115.07, 115.01 (d, *J* = 22.2 Hz, 2C), 108.53, 103.00, 99.14, 67.90, 55.72, 51.22, 32.90, 31.53, 27.84, 21.24, 15.40 (2C).
**$^{19}$F NMR** (376 MHz, DMSO-$d_6$): δ -118.98 – -119.10 (m).
**Elemental analysis**: for $C_{33}H_{32}FN_3O_7$ calcld.: C, 65.88; H, 5.36; found: C, 65.95; H, 5.30.
**MS (MALDI-TOF, pos. mode):** m/z $[M+H]^+$ for $C_{33}H_{32}FN_3O_7$ calcld.: 602.24; found: 602.00.

**Methyl 11-((4-(4-(1-((4-fluorophenyl)carbamoyl)cyclopropane-1-carboxamido)phenoxy)-6-methoxyquinolin-7-yl)oxy)undecanoate 4b**

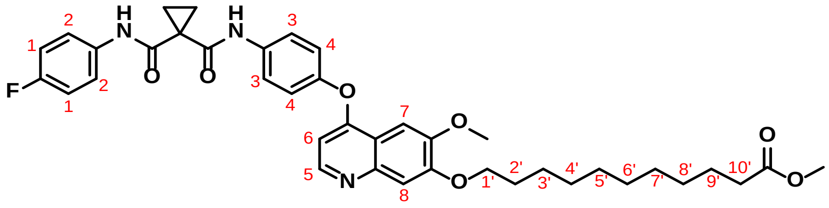

Eluent: ethyl acetate-methanol (1:0 → 9:1). Pale-yellow solid, yield 63%.

**M.p.** = 120 – 121 °C.
**$^1$H NMR** (400 MHz, DMSO-$d_6$): δ 10.17 (s, *NH*, 1H), 10.04 (s, *NH*, 1H), 8.45 (d, *J* = 5.2 Hz, *C5-H*, 1H), 7.75 (d, *J* = 9.0 Hz, *C3-H*, 2H), 7.66-7.59 (m, *C2-H*, 2H), 7 .49 (s, *C7-H*, 1H), 7 .36 (s, *C8 -H*, 1H), 7.22 (d, *J* = 9.0 Hz, *C4-H*, 2H), 7.18 – 7.11 (m, *C1-H*, 2H), 6.42 (d, J = 5.2 Hz, *C6 -H* ,1H), 4.13 (t, *J* = 6.5 Hz, *C1'-H*, 2H), 3.92 (s, *OMe*, 3H), 3.57 (s, *COOMe*, 3H), 2.28 (t, *J* = 7.4 Hz, *C10'-H*, 2H), 1.84 – 1.75 (m, *C2'-H*, 2H), 1.55 – 1.40 (m, *C3'-H*, *C9'-H*, $C_{cyclopropane}$-*H*, 8H), 1.40-1.20 (m, *C4'-H*, *C5'-H*, *C6'-H*, *C7'-H*, *C8'-H*, 10H).
**$^{13}$C NMR** (101 MHz, DMSO -$d_6$): δ 173.46, 168.26, 168.21, 159.99, 158.35 (d, *J* = 240.1 Hz), 151.97, 149.58, 149.50, 148.83, 146.47, 136.39, 135.17 (d, *J* = 2.7 Hz), 122.51 (d, *J* = 7.7 Hz, 2C), 122.26 (2C), 121.20 (2C), 115.20, 115.09 (d, *J* = 22.2 Hz, 2C), 108.48, 103.05, 99.18, 68.34, 55.78, 51.22, 33.33, 31.57, 28.96, 28.86, 28.76, 28.69, 28.50 (2C), 25.58, 24.48, 15.48 (2C).
**$^{19}$F NMR** (376 MHz, DMSO-$d_6$): δ -118.93 – -119.05 (m).
**Elemental analysis**: for $C_{39}H_{44}FN_3O_7$ calcld.: C, 68.30; H, 6.47; found: C, 68.41; H, 6.40.
**MS (MALDI-TOF, pos. mode):** m/z $[M+H]^+$ for $C_{39}H_{44}FN_3O_7$ calcld.: 686.3; found: 686.5.

## Synthesis of cabozantinib-based acids with aliphatic linker 5a, 5b

### General procedure for synthesis of acids 5a, 5b

A solution of corresponding ester **4a-b** (1.00 equiv.) and NaOH (6.00 equiv.) in mixture of solvents MeOH:$H_2O$ = 2:1 (5 ml) was stirred at 60 °C for 12 h. The reaction mixture was concentrated under reduced pressure, poured into saturated $NH_4Cl$ solution (10 mL) and the product was extracted with EtOAc (3×5 mL). The organic layer was dried over $Na_2SO_4$, filtered and evaporated under reduced pressure to give corresponding product.

**5-((4-(4-(1-((4-fluorophenyl)carbamoyl)cyclopropane-1-carboxamido)phenoxy)-6-methoxyquinolin-7-yl)oxy)pentanoic acid 5a**

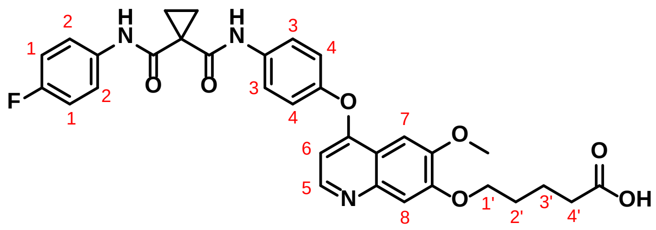


Pale-beige powder, yield 99%.

**M.p.** = 133 – 134 °C.
**$^{1}$H NMR** (400 MHz, DMSO-*d$_6$*): δ 10.18 (s, *NH*, 1H), 10.06 (s, *NH*, 1H), 8.46 (d, *J* = 5.2 Hz, *C5-H*, 1H), 7.76 (d, *J* = 9.0 Hz, *C3-H*, 2H), 7.68-7.61 (m, *C2-H*, 2H), 7 .50 (s, *C7-H*, 1H), 7 .39 (s, *C8 -H*, 1H), 7.25 – 7.20 (m, *C4-H*, 2H), 7.19 – 7.11(m, *C1-H*, 2H), 6.42 (d, *J* = 5.2 Hz, *C6 -H* ,1H), 4.15 (t, *J* = 6.3 Hz, *C1'-H*, 2H), 3.93 (s, *OMe*, 3H), 2.33 (t, *J* = 7.4 Hz, *C4'-H*, 2H), 1.88 – 1.79 (m, *C2'-H*, 2H), 1.76 – 1.66 (m, *C3'-H*, 2H), 1.46 (s, *C$_{cyclopropane}$-H*, 4H).
**$^{13}$C NMR** (101 MHz, DMSO-*d$_6$*): δ 174.42, 168.17, 168.13, 159.93, 158.28 (d, *J* = 240.1 Hz), 151.85, 149.52, 149.43, 148.77, 146.45, 136.37, 135.17 (d, *J* = 2.6 Hz), 122.42 (d, *J* = 7.9 Hz, 2C), 122.18 (2C), 121.15 (2C), 115.08, 115.02 (d, *J* = 22.2 Hz, 2C), 108.52, 103.01, 99.15, 67.99, 55.73, 33.31, 31.54, 27.96, 21.29, 15.42 (2C).
**$^{19}$F NMR** (376 MHz, DMSO-*d$_6$*): δ -118.96 – -119.15 (m).
**Elemental analysis**: for $C_{32}H_{30}FN_3O_7$ calcld.: C, 65.41; H, 5.15; found: C, 65.49; H, 5.20.
**MS (MALDI-TOF, pos. mode):** m/z $[M+H]^+$ for $C_{32}H_{30}FN_3O_7$ calcld.: 588.21; found: 588.77.

**11-((4-(4-(1-((4-fluorophenyl)carbamoyl)cyclopropane-1-carboxamido)phenoxy)-6-methoxyquinolin-7-yl)oxy)undecanoic acid 5b**

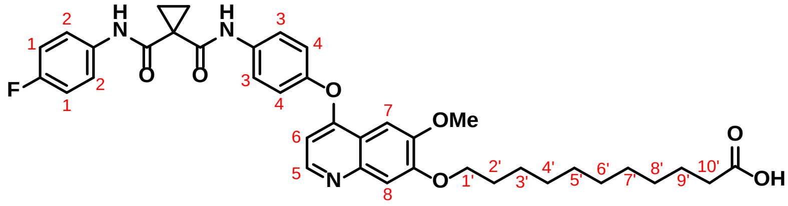


White solid, yield 82%.

**M.p.** = 140 – 141 °C.
**$^{1}$H NMR** (400 MHz, DMSO-*d$_6$*): δ 11.96 (s, *COOH*, 1H), 10.17 (s, *NH*, 1H), 10.05 (s, *NH*, 1H), 8.45 (d, *J* = 5.2 Hz, *C5-H*, 1H), 7.76 (d, *J* = 9.0 Hz, *C3-H*, 2H), 7.67-7.60 (m, *C2-H*, 2H), 7 .50 (s, *C7-H*, 1H), 7 .37 (s, *C8 -H*, 1H), 7.22 (d, *J* = 9.0 Hz, *C4-H*, 2H), 7.17 – 7.12 (m, *C1-H*, 2H), 6.42 (d, *J* = 5.2 Hz, *C6 -H* ,1H), 4.13 (t, *J* = 6.5 Hz, *C1'-H*, 2H), 3.93 (s, *C11'-H*, 3H), 2.18 (t, *J* = 7.4 Hz, *C10'-H*, 2H), 1.85 – 1.73 (m, *C2'-H*, 2H), 1.53-1.20 (m, *C3'-H, C4'-H, C5'-H, C6'-H, C7'-H, C8'-H, C9'-H, C$_{cyclopropane}$-H*, 18H).
**$^{13}$C NMR** (101 MHz, DMSO-*d$_6$*): δ 174.49, 168.14, 168.10, 159.91, 158.25 (d, *J* = 240.0 Hz), 151.90, 149.49, 149.43, 148.75, 146.44, 136.36, 135.16 (d, *J* = 2.6 Hz), 122.40 (d, *J* = 7.8 Hz, 2C), 122.15 (2C), 121.13 (2C), 115.02 (d, *J* = 22.1 Hz, 2C), 115.01, 108.45, 102.97, 99.11, 68.27, 55.71, 33.66, 31.54, 28.94, 28.85, 28.72, 28.55 (2C), 28.46, 25.54, 24.49, 15.38 (2C).
**$^{19}$F NMR** (376 MHz, DMSO-*d$_6$*): δ -118.99 – -119.11 (m).
**Elemental analysis**: for $C_{38}H_{42}FN_3O_7$ calcld.: C, 67.94; H, 6.30; found: C, 67.88; H, 6.25.
**MS (MALDI-TOF, pos. mode):** m/z $[M+H]^+$ for $C_{38}H_{42}FN_3O_7$ calcld.: 672.3; found: 672.3.

## Synthesis of cabozantinib-based esters with polyethylene- and polypropylene glycol linkers 6a, 6b

### General procedure for synthesis of esters 6a, 6b

A solution of cabozantinib hydroxyl derivative **3** (1.00 equiv.), $Cs_2CO_3$ (2.00 equiv.) was added and *tert*-butyl ester of corresponding terminal iodoalkane acid (2.00 equiv.) in DMF (2 mL) was stirred for 1 h at 60 °C. The solvent was removed under reduced pressure and the residue was purified using column chromatography to give the corresponding ethers.

**Tert-butyl 2-(2-(2-(2-((4-(4-(1-((4-fluorophenyl)carbamoyl)cyclopropane-1-carboxamido)phenoxy)-6-methoxyquinolin-7-yl)oxy)ethoxy)ethoxy)ethoxy)acetate 6a**

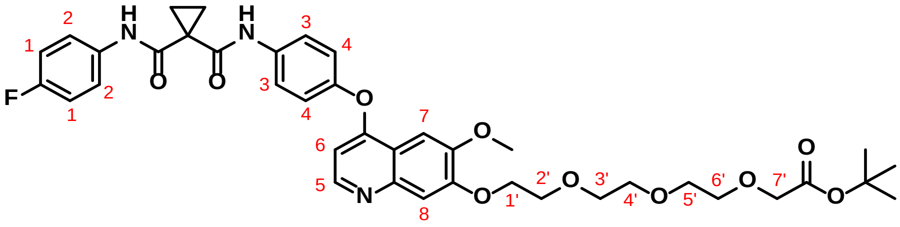

Eluent: chloroform – methanol (29:1 → 19:1), pale-yellow solid, yield 69 %.

**M.p.** = 134 – 135 °C.
**$^1$H NMR** (400 MHz, $CDCl_3$): δ 9.59 (s, *NH*, 1H), 9.09 (s, *NH*, 1H), 8.44 (d, *J* = 5.2 Hz, *C5-H*, 1H), 7.61 (d, *J* = 8.8 Hz, *C3-H*, 2H), 7 .51 (s, *C7-H*, 1H), 7.50-7.44 (m, *C2-H*, 2H), 7 .36 (s, *C8-H*, 1H), 7.14 (d, *J* = 8.8 Hz, *C4-H*, 2H), 7.06 – 6.97 (m, *C1-H*, 2H), 6.42 (d, *J* = 5.3 Hz, *C6 -H* , 1H), 4.30 (t, *J* = 4.8 Hz, *C1'-H*, 2H), 4.02 – 3.94 (m, *C6'-H, C7'-H, OMe*, 7H), 3.78 – 3.72 (m, *C2'-H*, 2H), 3.72 – 3.65 (m, *C3'-H, C4'-H, C5'-H*, 6H), 1.75 – 1.60 (m, $C_{cyclopropane}$-*H*, 4H), 1.45 (s, *COOtBu*, 9H).
**$^{13}$C NMR** (100 MHz, $CDCl_3$): δ 169.81, 169.49, 169.05, 160.70, 159.81 (d, *J* = 244.5 Hz), 152.21, 150.98, 149.84, 148.86, 146.83, 134.99, 133.33 (d, *J* = 3.3 Hz), 122.90 (d, *J* = 8.1 Hz, 2C), 122.59 (2C), 121.72 (2C), 116.26, 115.82 (d, *J* = 22.5 Hz, 2C), 108.82, 103.46, 99.72, 81.71, 70.99, 70.82, 70.73, 70.71, 69.37, 69.12, 68.30, 56.18, 29.30, 28.21 (3C), 17.68 (2C).
**Elemental analysis**: for $C_{39}H_{44}FN_3O_{10}$ calcld.: C, 63.84; H, 6.04; found: C, 63.94; H, 5.98.
**$^{19}$F NMR** (376 MHz, $CDCl_3$): δ -121.01 – -121.13 (m).
**HRMS (ESI):** m/z $[M+H]^+$ for $C_{39}H_{44}FN_3O_{10}$ calcld.: 734.3083; found: 734.2926.

**Tert-butyl 3-(3-(3-((4-(4-(1-((4-fluorophenyl)carbamoyl)cyclopropane-1-carboxamido)phenoxy)-6-methoxyquinolin-7-yl)oxy)propoxy)propoxy)propanoate 6b**

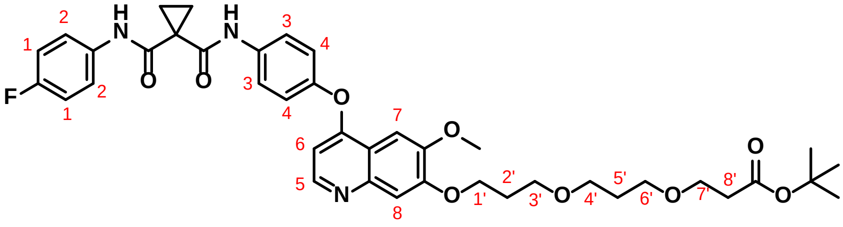

Eluent: chloroform – methanol (29:1 → 19:1), white solid, yield 81%.

**M.p.** = 132 – 133 °C.

**$^{1}$H NMR** (400 MHz, $CDCl_3$): δ 9.70 (s, *NH*, 1H), 9.22 (s, *NH*, 1H), 8.44 (d, J = 5.2 Hz, *C5-H*, 1H), 7.61 (d, J = 8.8 Hz, *C3-H*, 2H), 7 .51 (s, *C7-H*, 1H), 7.46 (dd, J = 8.9, 4.8 Hz, *C2-H*, 2H), 7.38 (s, *C8 -H*, 1H), 7.14 (d, J = 8.8 Hz, *C4-H*, 2H), 7.01 (t, J = 8.6 Hz, *C1-H*, 2H), 6.42 (d, J = 5.2 Hz, *C6 -H* ,1H), 4.25 (t, J = 6.3 Hz, *C1'-H*, 2H), 4.00 (s, *OMe*, 3H), 3.66 – 3.56 (m, *C3'-H*, *C7'-H*, 4H), 3.53 – 3.44 (m, *C4'-H*, *C6'-H*, 4H), 2.44 (t, *J* = 6.5 Hz, *C8'-H*, 2H), 2.22 – 2.12 (m, *C2'-H*, 2H), 1.85 – 1.77 (m, *C5'-H*, 2H), 1.73 – 1.61 (m, $C_{cyclopropane}$-*H*, 4H), 1.42 (s, *tBu*, 9H).
**$^{13}$C NMR** (100 MHz, $CDCl_3$): δ 171.15, 169.63, 169.12, 160.71, 159.78 (d, J = 244.5 Hz), 152.40, 150.99, 149.89, 148.76, 146.90, 134.99, 133.35 (d, J = 3.2 Hz), 122.93 (d, J = 8.1 Hz, 2C), 122.61 (2C), 121.67 (2C), 116.04, 115.77 (d, J = 22.6 Hz, 2C), 108.65, 103.37, 99.62, 80.58, 77.16, 68.00, 67.97, 67.37, 66.54, 66.14, 56.22, 30.07, 29.38, 29.23, 28.18 (3C), 17.70 (2C).
**$^{19}$F NMR** (376 MHz, $CDCl_3$): δ -122.00 – -121.12 (m).
**Elemental analysis**: for $C_{40}H_{46}FN_3O_9$ calcld.: C, 65.65; H, 6.34; found: C: C, 65.60; H, 6.29
**HRMS (ESI):** m/z $[M+H]^+$ for $C_{40}H_{46}FN_3O_9$ calcld.: 732.3291; found: 732.3413.

## Synthesis of cabozantinib-based acids with polyethylene- and polypropylene glycol linkers 7a, 7b

### General procedure for synthesis of acids 7a, 7b

A solution of corresponding *tert*-butyl ester **6a-b** (1.00 equiv.) in TFA (1 mL) was stirred at room temperature for 2 h. The solvent was evaporated under reduced pressure and the crude product was dried in vacuum to give the desired product in quantitative yield.

### 2-(2-(2-(2-((4-(4-(1-((4-fluorophenyl)carbamoyl)cyclopropane-1-carboxamido)phenoxy)-6-methoxyquinolin-7-yl)oxy)ethoxy)ethoxy)ethoxy)acetic acid 7a

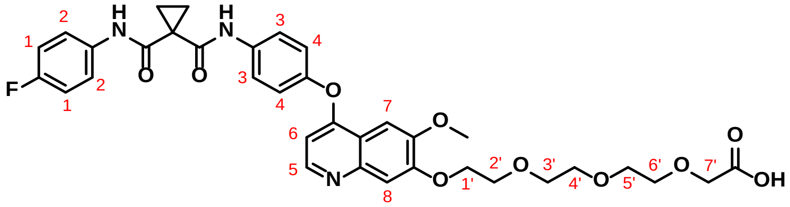

**M.p.** = 139 – 140 °C.
**$^{1}$H NMR** (400 MHz, DMSO-$d_6$): δ 10.34 (s, *NH*, 1H), 10.04 (s, *NH*, 1H), 8.81 (d, *J* = 6.6 Hz, *C5-H*, 1H), 7.86 (d, *J* = 8.9 Hz, *C3-H*, 2H), 7.74 (s, *C7-H*, 1H), 7.69-7.60 (m, *C2-H*, *C8 -H*, 3H), 7.38 (d, *J* = 8.8 Hz, *C4-H*, 2H), 7.19 – 7.11 (m, *C1-H*, 2H), 6.82 (d, *J* = 6.6 Hz, *C6 -H* ,1H), 4.38 – 4.34 (m, *C1'-H*, 2H), 4.04 (s, *OMe*, 3H), 4.01 (s, *C7'-H*, 2H), 3.92 – 3.87 (m, *C6'-H*, 2H), 3.67 – 3.61 (m, *C2'-H*, 2H), 3.60 – 3.53 (m, *C3'-H*, *C4'-H*, *C5'-H*, 6H), 1.50 (s, $C_{cyclopropane}$-*H*, 4H).
**$^{13}$C NMR** (101 MHz, DMSO-$d_6$): δ 171.66, 168.28, 168.22, 158.73, 158.33 (d, *J* = 240.2 Hz), 155.24, 151.10, 147.92, 143.36, 137.80, 137.28, 135.20 (d, *J* = 3.1 Hz), 122.47 (d, *J* = 8.1 Hz, 2C), 122.25 (2C), 121.42 (2C), 115.34, 115.07 (d, *J* = 22.2 Hz, 2C), 103.24, 100.85, 100.40,70.06, 69.86, 69.77 (2C), 68.98, 68.41, 67.58, 56.44, 31.73, 15.44 (2C).
**$^{19}$F NMR** (376 MHz, DMSO-$d_6$): δ -121.06 – -121.17 (m).
**Elemental analysis**: for $C_{35}H_{36}FN_3O_{10}$ calcld.: C, 62.03; H, 5.35; found: C, 62.10; H, 5.27.
**HRMS (ESI):** m/z $[M+H]^+$ for $C_{35}H_{36}FN_3O_{10}$ calcld.: 678.2457; found: 678.2530.

### 3-(3-(3-((4-(4-(1-((4-fluorophenyl)carbamoyl)cyclopropane-1-carboxamido)phenoxy)-6-methoxyquinolin-7-yl)oxy)propoxy)propoxy)propanoic acid 7b

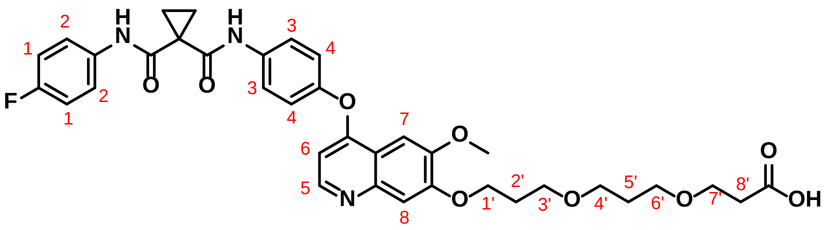

**M.p.** = 137 – 138 °C.
**$^{1}$H NMR** (400 MHz, DMSO-*d6*): δ 10.34 (s, *NH*, 1H), 10.04 (s, *NH*, 1H), 8.82 (d, *J* = 6.6 Hz, *C5-H*, 1H), 7.87 (d, *J* = 8.8 Hz, *C3-H*, 2H), 7.73 (s, *C7-H*, 1H), 7.68 – 7.61 (m, *C2-H*, *C8 -H*, 3H), 7.37 (d, *J* = 8.8 Hz, *C4-H*, 2H), 7.15 (t, *J* = 8.8 Hz, *C1-H*, 2H), 6.43 (d, *J* = 6.6 Hz, *C6 -H*, 1H), 4.28 (t, *J* = 5.9 Hz, *C1'-H*, 2H), 4.04 (s, *OMe*, 3H), 3.61 – 3.48 (m, *C3'-H*, *C7'-H*, 4H), 3.47 – 3.36 (m, *C4'-H*, *C6'-H*, 4H), 2.39 (t, *J* = 6.2 Hz, *C8'-H*, 2H), 2.14 – 2.05 (m, *C2'-H*, 2H), 1.77 – 1.66 (m, *C5'-H*, 2H), 1.40 (s, $C_{cyclopropane}$-*H*, 4H).
**$^{13}$C NMR** (100 MHz, DMSO-*d6*): δ 172.69, 168.31, 168.25, 158.79, 158.35 (d, *J* = 240.1 Hz), 155.40, 151.21, 147.90, 143.18, 137.86, 137.14, 135.22 (d, *J* = 3.0 Hz), 122.48 (d, *J* = 8.2 Hz, 2C), 122.28 (2C), 121.43 (2C), 115.30, 115.08 (d, *J* = 22.2 Hz, 2C), 103.19, 100.48, 100.39, 67.13 (2C), 66.63, 66.34, 65.93, 56.51, 34.75, 31.73, 29.52, 28.63, 15.46 (2C).
**$^{19}$F NMR** (376 MHz, DMSO-*d6*): δ -121.07 – -121.19 (m).
**Elemental analysis**: for $C_{36}H_{38}FN_3O_9$ calcld.: C, 63.99; H, 5.67; found: C, 64.07; H, 5.62.

## Synthesis of alkynyl cabozantinib derivatives 8a-d

### General procedure for synthesis of derivatives 8a-d

A solution of cabozantinib derivative **3** (1.00 equiv.) and $Cs_2CO_3$ (3.00 equiv.) in DMF (10 mL) in inert atmosphere was stirred at room temperature for 15 min. Then the mixture was treated with the corresponding haloalkyne (2.00 equiv.) and stirred at room temperature for 12 h. Solvent was removed under reduced pressure and the residue was purified by column chromatography.

***N*-(4-fluorophenyl)-*N*-(4-((6-methoxy-7-(prop-2-yn-1-yloxy)quinolin-4-yl)oxy)phenyl)cyclopropane-1,1-dicarboxamide 8a**

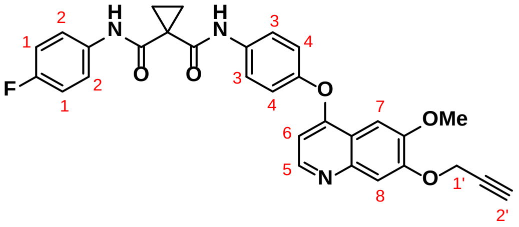

Eluent: petroleum ester-ethyl acetate (1:1 → 0:1). Pale-yellow solid, yield 73%.

**M.p.** = 110 – 111 °C.
**$^{1}$H NMR** (400 MHz, DMSO-*d6*): δ 10.18 (s, *NH*, 1H), 10.05 (s, *NH*, 1H), 8.48 (d, *J* = 5.2 Hz, *C5-H*, 1H), 7.76 (d, *J* = 9.0 Hz, *C3-H*, 2H), 7.67-7.61 (m, *C2-H*, 2H), 7 .53 (s, *C7-H*, 1H), 7.50 (s, *C8 -H*, 1H), 7.25 – 7.20 (m, *C4-H*, 2H), 7.18 – 7.11 (m, *C1-H*, 2H), 6.45 (d, *J* = 5.2 Hz, *C6 -H*, 1H), 5.01 (d, *J* = 2.3 Hz, *C1'-H*, 2H), 3.94 (s, *OMe*, 3H), 3.65 (t, *J* = 2.3 Hz, *C2'-H*, 1H), 1.48 (s, $C_{cyclopropane}$-*H*, 4H).

$^{13}$**C NMR** (101 MHz, DMSO-*d*$_6$): δ, 168.14, 168.11, 159.98, 158.26 (d, *J* = 240.1 Hz), 150.16, 149.45, 149.31, 148.97, 146.01, 136.41, 135.16 (d, *J* = 2.6 Hz), 122.41 (d, *J* = 7.8 Hz, 2C), 122.17 (2C), 121.15 (2C), 115.59, 115.01 (d, *J* = 22.2 Hz, 2C), 109.62, 103.26, 99.40, 78.86, 78.68, 56.06, 55.76, 31.55, 15.39 (2C).
$^{19}$**F NMR** (376 MHz, DMSO-*d*$_6$): δ -118.99-119.13 (m).
**Elemental Analysis**: for $C_{30}H_{24}FN_3O_5$ calcld.: C, 68.56; H, 4.60; found: C, 68.51; H, 4.67.
**MS (MALDI-TOF, pos. mode):** m/z $[M+H]^+$ for $C_{30}H_{24}FN_3O_5$ calcld.: 526.18; found: 526.19.

***N*-(4-fluorophenyl)-*N*-(4-((6-methoxy-7-(pent-4-yn-1-yloxy)quinolin-4-yl)oxy)phenyl)cyclopropane-1,1-dicarboxamide 8b**

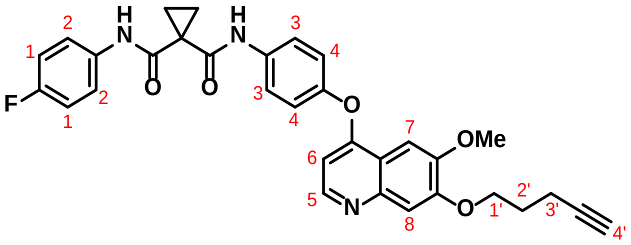


Eluent: petroleum ester - ethyl acetate (1:1 → 0:1). Pale-yellow solid, yield 87%.

**M.p.** = 113 – 114 °C.
$^{1}$**H NMR** (400 MHz, DMSO-*d*$_6$): δ 10.18 (s, *NH*, 1H), 10.05 (s, *NH*, 1H), 8.47 (d, *J* = 5.2 Hz, *C5-H*, 1H), 7.77 (d, *J* = 9.0 Hz, *C3-H*, 2H), 7.67-7.61 (m, *C2-H*, 2H), 7 .52 (s, *C7-H*, 1H), 7.40 (s, *C8 -H*, 1H), 7.26 – 7.20 (m, *C4-H*, 2H), 7.18 – 7.11 (m, *C1-H*, 2H), 6.44 (d, *J* = 5.2 Hz, *C6 -H*, 1H), 4.22 (t, *J* = 6.2 Hz, *C1'-H*, 2H), 3.94 (s, *OMe*, 3H), 2.85 (t, *J* = 2.6 Hz, *C4'-H*, 1H), 2.42 – 2.36 (m, *C3'-H*, 2H), 2.04 – 1.96 (m, *C2'-H*, 2H), 1.48 (s, $C_{cyclopropane}$-*H*, 4H).
$^{13}$**C NMR** (101 MHz, DMSO-*d*$_6$): δ 168.14, 168.10, 160.11, 158.26 (d, *J* = 240.2 Hz), 151.79, 149.44, 149.43, 148.64, 146.06, 136.42, 135.16 (d, *J* = 2.6 Hz), 122.40 (d, *J* = 7.8 Hz, 2C), 122.16 (2C), 121.14 (2C), 115.17, 115.01 (d, *J* = 22.2 Hz, 2C), 108.30, 103.06, 99.28, 83.55, 71.76, 66.77, 55.77, 31.54, 27.53, 15.39, 14.54 (2C).
$^{19}$**F NMR** (376 MHz, DMSO-*d*$_6$): δ -118.99 – -119.09 (m).
**Elemental analysis**: for $C_{32}H_{28}FN_3O_5$ calcld.: C, 69.43; H, 5.10; found: C, C, 69.51; H, 5.00.
**MS (MALDI-TOF, pos. mode):** m/z $[M+H]^+$ for $C_{32}H_{28}FN_3O_5$ calcld.: 554.2; found: 554.0.

**N-(4-fluorophenyl)-N-(4-((7-(hex-5-yn-1-yloxy)-6-methoxyquinolin-4-yl)oxy)phenyl)cyclopropane-1,1-dicarboxamide 8c**

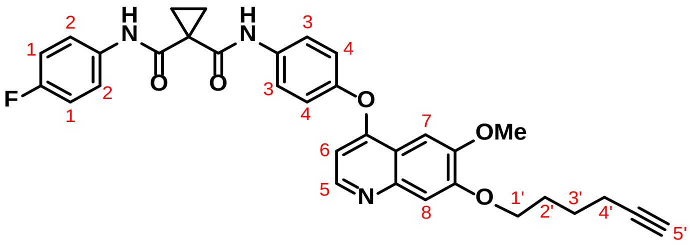


Eluent: ethyl acetate-methanol (1:0 → 5:1). White solid, yield 54%.

**M.p.** = 117 – 118 °C.
$^{1}$**H NMR** (400 MHz, DMSO-*d*$_6$): δ 10.18 (s, *NH*, 1H), 10.05 (s, *NH*, 1H), 8.45 (d, *J* = 5.2 Hz, *C5-H*, 1H), 7.76 (d, *J* = 8.4 Hz, *C3-H*, 2H), 7.68-7.61 (m, *C2-H*, 2H), 7.50 (s, *C7-H*, 1H), 7.39 (s, *C8*

-*H*, 1H), 7.22 (d, *J* = 8.2 Hz, *C4-H*, 2H), 7.18 – 7.11 (m, *C1-H*, 2H), 6.42 (d, *J* = 5.2 Hz, *C6-H*, 1H), 4.17 (t, *J* = 6.2 Hz, *C1'-H*, 2H), 3.93 (s, *OMe*, 3H), 2.81 – 2.78 (m, *C5'-H*, 1H), 2.42 – 2.36 (m, *C4'-H*, 2H), 2.04 – 1.96 (m, *C2'-H*, 2H), 1.71 – 1.58 (m, *C3'-H*, 2H), 1.48 (s, $C_{Cyclopropane}$-*H*, 4H).
**$^{13}$C NMR** (101 MHz, DMSO-$d_6$): δ 168.16, 168.11, 159.92, 158.27 (d, *J* = 240.1 Hz), 151.82, 149.51, 149.41, 148.77, 146.44, 136.37, 135.16 (d, *J* = 2.6 Hz), 122.41 (d, *J* = 7.8 Hz, 2C), 122.17 (2C), 121.13 (2C), 115.08, 115.01 (d, *J* = 22.2 Hz, 2C), 108.53, 103.00, 99.14, 84.34, 71.41, 67.77, 55.73, 31.52, 27.62, 24.70, 17.46, 15.41 (2 C).
**$^{19}$F NMR** (376 MHz, DMSO-$d_6$): δ -118.81 – -118.99 (m).
**Elemental analysis**: for $C_{33}H_{30}FN_3O_5$ calcld.: C, 69.83; H, 5.33; found: C, 69.95; H, 5.21.
**MS (MALDI-TOF, pos. mode):** m/z $[M+H]^+$ for $C_{33}H_{30}FN_3O_5$ calcld.: 568.2; found: 568.0.

**N-(4-fluorophenyl)-N-(4-((6-methoxy-7-(undec-10-yn-1-yloxy)quinolin-4-yl)oxy)phenyl)cyclopropane-1,1-dicarboxamide 8d**

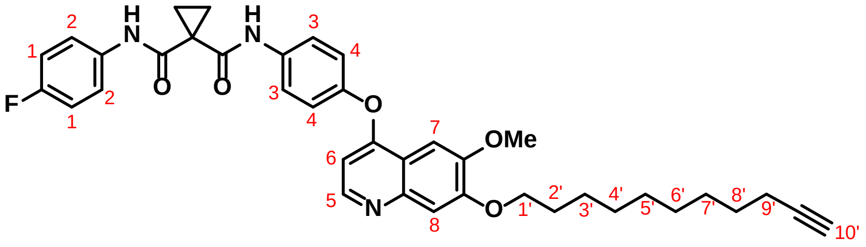

Eluent: ethyl acetate-methanol (1:1 → 9:1). Oily yellowish solid, yield 59%.

**M.p.** = 120 – 121 °C.
**$^1$H NMR** (400 MHz, DMSO-$d_6$): δ 10.18 (s, *NH*, 1H), 10.06 (s, *NH*, 1H), 8.45 (d, *J* = 5.2 Hz, *C5-H*, 1H), 7.76 (d, *J* = 9 Hz, *C3-H*, 2H), 7.67-7.62 (m, *C2-H*, 2H), 7.50 (s, *C7-H*, 1H), 7.37 (s, *C8 -H*, 1H), 7.22 (d, *J* = 9 Hz, *C4-H*, 2H), 7.18 – 7.12 (m, *C1-H*, 2H), 6.42 (d, *J* = 5.2 Hz, C6 -H, 1H), 4.13 (t, *J* = 6.5 Hz, *C1'-H*, 2H), 3.93 (s, *OMe*, 3H), 2.72 (t, *J* = 2.6 Hz, *C10'-H*, 1H), 2.18-2.10 (m, *C9'-H*, 2H), 1.85-1.76 (m, *C2'-H*, 2H), 1.51-1.25 (m, *C3'-H, C4'-H, C5'-H, C6'-H, C7'-H, C8'-H*, $C_{cyclopropane}$-*H*, 16H).
**$^{13}$C NMR** (101 MHz, DMSO-$d_6$): δ 168.16, 168.11, 159.92, 158.27 (d, *J* = 240.1 Hz), 151.90, 149.51, 149.43, 148.73, 146.44, 136.36, 135.16 (d, *J* = 2.6 Hz), 122.40 (d, *J* = 7.9 Hz, 2C), 122.16 (2C), 121.12(2C), 115.03, 115.00 (d, *J* = 22.2 Hz, 2C), 108.44, 102.97, 99.12, 84.54, 71.77, 68.27, 55.70, 31.51, 28.89, 28.71, 28.46, 28.43, 28.11, 27.96, 25.53, 17.66, 15.42 (2C).
**$^{19}$F NMR** (376 MHz, DMSO-$d_6$): δ -118.98 – -119.10 (m).
**Elemental analysis**: for $C_{38}H_{40}FN_3O_5$ calcld.: C, 71.57; H, 6.32; found: C, 71.50; H, 6.39.
**MS (MALDI-TOF, pos. mode):** m/z $[M+H]^+$ for $C_{38}H_{40}FN_3O_5$ calcld.: 638.30; found: 638.59.

## Synthesis of cabozantinib-based acids with triazole linker 9a-d

### General procedure for synthesis of acids 9a-d

To a solution of corresponding alkynyl cabozantinib derivative **8a-d** (1.00 equiv.) and 2-azidoacetic acid (1.00 equiv.) in DMF (3 mL) was added a mixture of $CuSO_4*5H_2O$ (0.20 equiv.), TBTA (0.40 equiv.), AscNa (0.20 equiv.) and $H_2O$ (3 mL). The reaction mixture was stirred at 55

°C for 1 h and solvent was evaporated under reduced pressure. The crude product was purified by column chromatography.

**3-(4-(((4-(4-(1-((4-fluorophenyl)carbamoyl)cyclopropane-1-carboxamido)phenoxy)-6-methoxyquinolin-7-yl)oxy)methyl)-1H-1,2,3-triazol-1-yl)propanoic acid 9a**

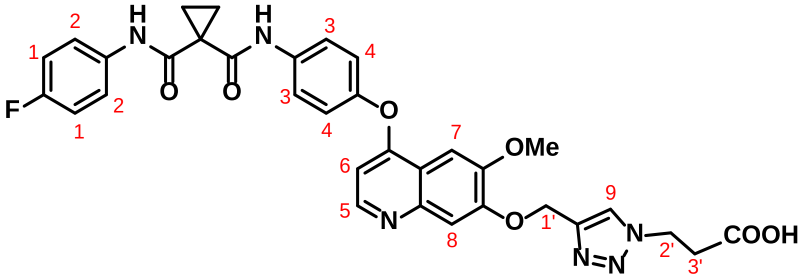


Eluent: ethyl acetate-methanol (9:1 → 7:3). Pale-yellow solid, yield 64%.

**M.p.** = 115 – 116 °C.
**$^1$H NMR** (400 MHz, DMSO-*d*$_6$): δ 10.25 (s, *NH*, 1H), 10.13 (s, *NH*, 1H), 8.45 (d, *J* = 5.2 Hz, *C5-H*, 1H), 8.28 (s, *C9-H*, 1H),7.77 (d, *J* = 9.0 Hz, *C3-H*, 2H), 7.68-7.62 (m, *C2-H*, *C7-H*, 3H), 7 .51 (s, *C8 -H*, 1H), 7.22 (d, *J* = 9.0 Hz, *C4-H*, 2H), 7.18 – 7.11 (m, *C1-H*, 2H), 6.43 (d, *J* = 5.2 Hz, *C6 -H* ,1H), 5.29 (s, *C1'-H*, 2H), 4.56 (t, *J* = 6.8 Hz, *C2'-H*, 2H), 3.90 (s, *OMe*, 3H), 3.65 (t, *J* = 2.3 Hz, *C2'-H*, 2H), 2.76 (t, *J* = 6.7 Hz, *C3'-H*, 2H), 1.48 (s, $C_{cyclopropane}$*-H*, 4H).
**$^{13}$C NMR** (101 MHz, DMSO -d$_6$): δ 172.48, 168.18 (d, *J* = 2.9 Hz, 2C, 168.19, 168.16), 159.95, 158.25 (d, J = 240.2 Hz), 151.23, 149.46, 149.32, 148.85, 146.29, 141.79, 136.42, 135.20 (d, *J* = 2.6 Hz), 125.11, 122.39 (d, *J* = 7.9 Hz, 2C), 122.16 (2C), 121.15 (2C), 115.31, 115.00 (d, *J* = 22.2 Hz, 2C), 109.10, 103.11, 99.22, 61.68, 55.64, 46.31, 35.64, 31.62, 15.36 (2C).
**$^{19}$F NMR** (376 MHz, DMSO-*d*$_6$): δ -119.02 – -119.13 (m).
**Elemental analysis**: for $C_{33}H_{29}FN_6O_7$ calcld.: C, 61.87; H, 4.56; found: C, 62.98; H, 4.5.
**MS (MALDI-TOF, pos. mode):** m/z $[M+H]^+$ for $C_{33}H_{29}FN_6O_7$ calcld.: 641.22; found: 641.90.

**3-(4-(3-((4-(4-(1-((4-fluorophenyl)carbamoyl)cyclopropane-1-carboxamido)phenoxy)-6-methoxyquinolin-7-yl)oxy)propyl)-1H-1,2,3-triazol-1-yl)propanoic acid 9b**

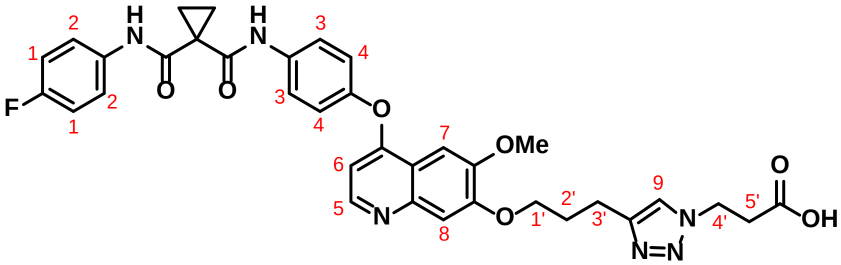


Eluent: ethyl acetate-methanol (1:0 → 5:1). Pale-yellow solid, yield 87%.

**M.p.** = 120 – 121 °C.
**$^1$H NMR** (400 MHz, DMSO-*d*$_6$): δ 10.22 (s, *NH*, 1H), 10.11 (s, *NH*, 1H), 8.45 (d, *J* = 5.2 Hz, *C5-H*, 1H), 7.90 (s, C9-H, 1H), 7.76 (d, *J* = 8.9 Hz, *C3-H*, 2H), 7.67-7.61 (m, *C2-H*, 2H), 7.50 (s, *C7-H*, 1H), 7 .37 (s, *C8 -H*, 1H), 7.22 (d, *J* = 9.0 Hz, *C4-H*, 2H), 7.18 – 7.12 (m, *C1-H*, 2H), 6.42 (d, *J* = 5.2 Hz, C6 -H, 1H), 4.46 (t, *J* = 6.9 Hz, *C4'-H*, 2H), 4.20 (t, *J* = 6.3 Hz, *C1'-H*, 2H), 3.94 (s,

*OMe*, 3H), 2.81 (t, *J* = 7.5 Hz, *C3'-H*, 2H), 2.63 (t, *J* = 7.0 Hz, C5'-H, 2H), 2.18 – 2.08 (dd, *J* = 14.0, 6.5 Hz, *C2'-H*, 2H), 1.47 (s, $C_{cyclopropane}$-*H*, 4H).
**$^{13}$C NMR** (101 MHz, DMSO-$d_6$): δ 172.47, 168.19, 168.17, 159.96, 158.28 (d, *J* = 240.0 Hz), 151.83, 149.51, 149.45, 148.80, 146.39, 145.84, 136.40, 135.20 (d, *J* = 2.6 Hz), 128.75, 122.42 (d, *J* = 7.8 Hz, 2C), 122.19 (2C), 121.17 (2C), 115.12, 115.03 (d, *J* = 22.2 Hz, 2C), 108.53, 103.03, 99.20, 67.61, 55.76, 46.53, 36.56, 31.61, 28.36, 21.71, 15.37 (2C).
**$^{19}$F NMR** (376 MHz, DMSO-$d_6$): δ -119.00 – -119.10 (m).
**Elemental analysis**: for $C_{35}H_{33}FN_6O_7$ calcld.: C, 62.87; H, 4.97; found: C, 62.95; H, 4.85.
**MS (MALDI-TOF, pos. mode):** m/z $[M+H]^+$ for $C_{35}H_{33}FN_6O_7$ calcld.: 669.2; found: 669.0.

**3-(4-(4-((4-(4-(1-((4-fluorophenyl)carbamoyl)cyclopropane-1-carboxamido)phenoxy)-6-methoxyquinolin-7-yl)oxy)butyl)-1H-1,2,3-triazol-1-yl)propanoic acid 9c**

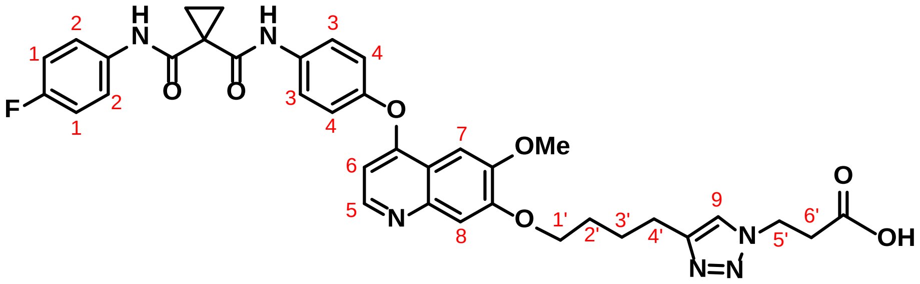

Eluent: ethyl acetate-methanol (1:0 → 7:3). Pale-yellow solid, yield 75%.

**M.p.** = 121 – 122 °C.
**$^{1}$H NMR** (400 MHz, DMSO-$d_6$): δ 10.22 (s, *NH*, 1H), 10.10 (s, *NH*, 1H), 8.45 (d, *J* = 5.2 Hz, *C5-H*, 1H), 7.84 (s, C9-H, 1H), 7.76 (d, *J* = 8.0 Hz, *C3-H*, 2H), 7.67-7.62 (m, *C2-H*, 2H), 7.50 (s, *C7-H*, 1H), 7.38 (s, *C8 -H*, 1H), 7.24-7.21 (m, *C4-H*, 2H), 7.17 – 7.12 (m, *C1-H*, 2H), 6.42 (d, *J* = 5.2 Hz, C6 -H ,1H), 4.47 (t, *J* = 6.9 Hz, *C5'-H*, 2H), 4.16 (t, *J* = 6.1 Hz, *C1'-H*, 2H), 3.93 (s, *OMe*, 3H), 2.76 – 2.67 (m, *C4'-H*, *C6'-H*, 4H), 1.90-1.74 (m, *C2'-H*, *C3'-H*, 4H), 1.48 (s, $C_{cyclopropane}$-*H*, 4H).
**$^{13}$C NMR** (101 MHz, DMSO-$d_6$): δ 172.40, 168.16, 168.12, 159.93, 158.25 (d, *J* = 240.1 Hz), 151.86, 149.48, 149.41, 148.74, 146.41, 146.39, 136.39, 135.19 (d, *J* = 2.6 Hz), 122.39 (d, *J* = 7.9 Hz, 2C), 122.15 (2C), 121.95, 121.14 (2C), 115.04, 115.00 (d, *J* = 22.2 Hz, 2C), 108.45, 102.98, 99.12, 68.04, 55.71, 45.90, 35.52, 31.59, 28.02, 25.59, 24.68, 15.35 (2C).
**$^{19}$F NMR** (376 MHz, DMSO-$d_6$): δ -119.01 – -119.12 (m).
**Elemental analysis**: for $C_{36}H_{35}FN_6O_7$ calcld.: C, 63.34; H, 5.17; found: C, 63.30; H, 5.23.
**MS (MALDI-TOF, pos. mode):** m/z $[M+H]^+$ for $C_{36}H_{35}FN_6O_7$ calcld.: 683.3; found: 683.0.

**3-(4-(9-((4-(4-(1-((4-fluorophenyl)carbamoyl)cyclopropane-1-carboxamido)phenoxy)-6-methoxyquinolin-7-yl)oxy)nonyl)-1H-1,2,3-triazol-1-yl)propanoic acid 9d**

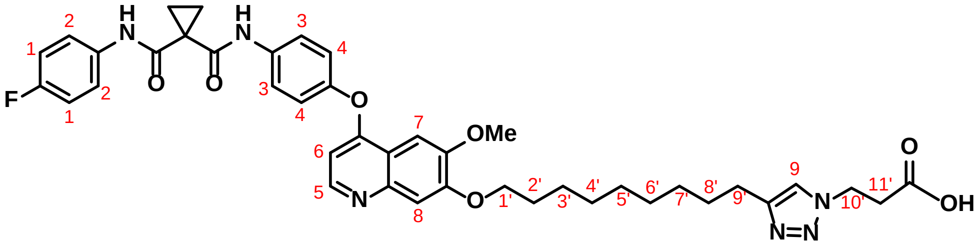

Eluent: ethyl acetate-methanol (1:1→ 5:1). Oily pale-yellow solid, yield 64%.

**M.p.** = 123 – 124 °C.
**$^{1}$H NMR** (400 MHz, DMSO-*d*$_6$): 400 MHz, DMSO-d$_6$) δ 10.24 (s, *NH*, 1H), 10.12 (s, *NH*, 1H), 8.45 (d, *J* = 5.2 Hz, *C5-H*, 1H), 7.80-7.75 (m, *C3-H, C9-H*, 3H), 7.68-7.62 (m, *C2-H*, 2H), 7 .49 (s, *C7-H*, 1H), 7.37 (s, *C8 -H*, 1H), 7.21 (d, *J* = 8.9 Hz, *C4-H*, 2H), 7.18 – 7.11 (m, *C1-H*, 2H), 6.41 (d, *J* = 5.2 Hz, C6 -H, 1H), 4.43 (t, *J* = 7.0 Hz, *C10'-H*, 2H), 4.13 (t, *J* = 6.4 Hz, *C1'-H*, 2H), 3.92 (s, *OMe*, 3H), 2.63 – 2.54 (m, *C9'-H, C11'-H*, 4H), 1.85 – 1.74 (m, *C2'-H*, 2H), 1.61 – 1.53 (m, *C8'-H*, 2H), 1.51-1.28 (*C3'-H, C4'-H, C5'-H, C6'-H, C7'-H*, $C_{cyclopropane}$-*H*, 14H)
**$^{13}$C NMR** (101 MHz, DMSO-*d*$_6$): 172.42, 168.16, 168.11, 159.93, 158.25 (d, *J* = 240.1 Hz), 151.85, 149.48, 149.43, 148.72, 146.44, 146.40, 136.36, 135.15 (d, *J* = 2.6 Hz), 122.39 (d, *J* = 7.9 Hz, 2C), 122.15 (2C), 121.95, 121.12 (2C), 115.03, 115.01 (d, *J* = 22.2 Hz, 2C), 108.43, 102.97, 99.12, 68.27, 55.70, 45.94, 35.52, 31.51, 28.89, 28.71, 28.46, 28.43, 28.11, 27.96, 26.00, 25.53, 15.42 (2C).
**$^{19}$F NMR** (376 MHz, DMSO-*d*$_6$): δ -118.98 – -119.10 (m).
**Elemental analysis**: for $C_{41}H_{45}FN_6O_7$ calcld.: C, 65.41; H, 6.03; found: C, 65.50; H, 5.90.
**MS (MALDI-TOF, pos. mode):** m/z $[M+H]^+$ for $C_{41}H_{45}FN_6O_7$ calcld.: 753.34; found: 753.97.

**Synthesis of tert-butyl (2-(2-(2-(4-((2-(2,6-dioxopiperidin-3-yl)-1-oxoisoindolin-4-yl) amino)-4-oxobutanamido)ethoxy)ethoxy)ethyl)carbamate 11a**

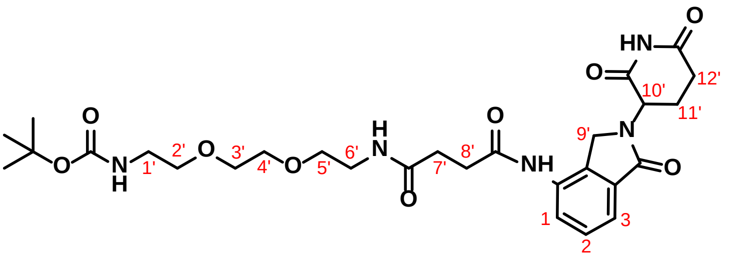

A solution of lenalidomide (1.00 equiv., 500 mg, 1.93 mmol) in DMF (10 mL) was treated with 2,2-dimethyl-4,15-dioxo-3,8,11-trioxa-5,14-diazaoctadecan-18-oic acid (**13**) (1.00 equiv., 672 mg, 1.93 mmol), DIPEA (2.50 equiv., 838 µL, 4.82 mmol), HATU (1.20 equiv., 880 mg, 2.32 mmol). Reaction was stirred at room temperature for 1 hour. The solvent was removed under reduced pressure. The lenalidomide derivative **17a** was isolated using column chromatography. Eluent: chloroform–methanol (1:0 → 35:2). Yellow solid, yield 47%.

**M.p.** = 279 – 280 °C.
**$^{1}$H NMR** (400 MHz, $CDCl_3$): δ 9.54 (s, *NH*, 1H), 9.42 (s, *NH*, 1H), 7.84 (d, *J* = 7.7 Hz, *C3-H*, 1H), 7.56 (d, *J* = 7.6 Hz, *C1-H*, 1H), 7.35 (t, *J* = 7.8 Hz, *C2-H*, 1H), 6.80-6.72 (m, *NH*, 1H), 5.24-5.16 (m, *NH*, 1H), 5.11 (dd, *J* = 12.9, 4.4 Hz, *C10'-H*, 1H), 4.35-4.21 (m, *C9'-H*, 2H), 3.58 – 3.44 (m, *C2'-H, C3'-H, C4'-H, C5'-H*, 8H), 3.41 – 3.33 (m, *C6'-H*, 2H), 3.31 – 3.21 (m, *C1'-H*, 2H), 2.76 – 2.55 (m, *C7'-H, C8'-H, C12'-H*, 6H), 2.29 – 2.12 (m, *C11'-H*, 1H), 2.10 – 1.95 (m, *C11'-H*, 1H), 1.40 (s, *tBu*, 9H).
**$^{13}$C NMR** (100 MHz, $CDCl_3$): δ 172.74, 172.37, 171.53, 170.55, 169.28, 156.19, 133.40, 133.29, 132.41, 129.07, 125.49, 120.25, 79.43, 70.21 (2C), 70.11, 69.55, 51.91, 46.48, 40.36, 39.48, 32.15, 31.56, 31.23, 28.50 (3C), 23.18.
**Elemental analysis**: for $C_{28}H_{39}N_5O_9$ calcld.: C, 57.04; H, 6.67; found: C, 57.13; H, 6.74.
**HRMS (ESI):** m/z $[M+H]^+$ for $C_{28}H_{39}N_5O_9$ calcld.: 590.2820; found: 590.2830.

**Synthesis of *N*[1]-(2-(2-(2-aminoethoxy)ethoxy)ethyl)-*N*[4]-(2-(2,6-dioxopiperidin-3-yl)-1-oxoisoindolin-4-yl)succinamide TFA salt 11b**

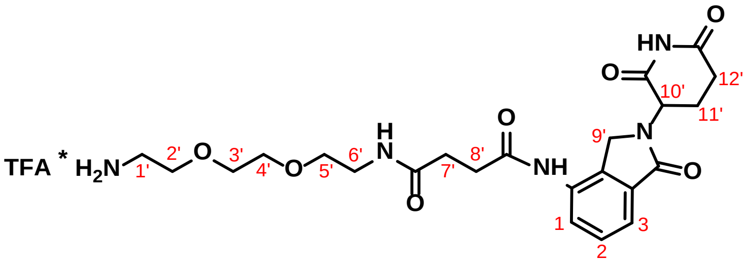


A solution of **11a** (1.00 equiv., 600 mg, 1.02 mmol) in a mixture of DCM (5 mL) and TFA (1.5 mL) was stirred at room temperature for 1 hour. The solvent was evaporated under reduced pressure. The yield of compound **11a** 90 %, yellow oil.

**M.p.** = 289 – 290 °C.
**$^1$H NMR** (400 MHz, DMSO-$d_6$): δ 11.05 (s, *NH*, 1H), 9.91 (s, *NH*, 1H), 7.99 (t, *J* = 5.5 Hz, *NH*, 1H), 7.90 – 7.79 (m, *C2-H*, *NH*, 3H), 7.53 – 7.45 (m, *C1-H*, *C3-H*, 2H), 7.27 – 7.11 (m, *NH*, 1H), 5.16 (dd, *J* = 13.3, 5.1 Hz, *C10'-H*, 1H), 4.35 (q, *J* = 17.6 Hz, *C9'-H*, 2H), 3.61 – 3.50 (m, *C3'-H*, *C4'-H*, *C5'-H*, 6H), 3.40 (t, *J* = 5.9 Hz, *C2'-H*, 2H), 3.24 – 3.18 (m, *C6'-H*, 2H), 3.01 – 2.88 (m, *C1'-H*, *C12'-H*, 3H), 2.65 – 2.57 (m, *C7'-H*, *C12'-H*, 3H), 2.43 (t, *J* = 7.1 Hz, *C8'-H*, 2H), 2.38 – 2.25 (m, *C11'-H*, 1H), 2.08 – 1.99 (m, *C11'-H*, 1H).
**$^{13}$C NMR** (100 MHz, DMSO-$d_6$): δ 173.0, 171.43, 171.18, 170.80, 167 .94, 158.47 (q, *J* = 35.9 Hz), 133.90, 133.57, 132.71, 128.71, 125.06, 118.98, 115.92 (q, *J* = 292.2 Hz), 69.71, 69.48, 69.16, 66.75, 51.57, 46.51, 38.68, 38.60, 31.28, 31.18, 30.30, 22.77.
**$^{19}$F NMR** (376 MHz, DMSO-$d_6$): δ -74.17 (s).
**Elemental analysis**: for $C_{25}H_{32}F_3N_5O_9$ calcld.: C, 49.75; H, 5.34; found: C, 49.67; H, 5.48.
**MS (MALDI-TOF, pos. mode):** m/z $[M+H]^+$ for $C_{23}H_{31}N_5O_7$ calcld.: 490.2; found: 490.3.

## Synthesis of cabozantinib-based PROTACs 15a-d, 16a, b, 17a-d

### General procedure for synthesis of PROTACs 15a-d, 16a, b, 17a-d

To the solution of corresponding E3-ligand (1.00 equiv.) in DMF (3 mL) under inert atmosphere were subsequently added corresponding cabozantinib-based acid **5a-b, 7a-b, 9a-d** (1.00 equiv.), DIPEA (2.50 equiv.) and HATU (1.20 equiv.). The reaction mixture was stirred at room temperature for 1.5 hour with subsequent removal of solvent under reduced pressure. The crude residue was purified using column chromatography to obtain corresponding product.

***N*-(4-fluorophenyl)-*N*-(4-((7-(((*S*)-13-((2*S*,4*R*)-4-hydroxy-2-((4-(4-methylthiazol-5-yl)benzyl)carbamoyl)pyrrolidine-1-carbonyl)-14,14-dimethyl-11-oxo-3,6,9-trioxa-12-azapentadecyl)oxy)-6-methoxyquinolin-4-yl)oxy)phenyl)cyclopropane-1,1-dicarboxamide 15a**

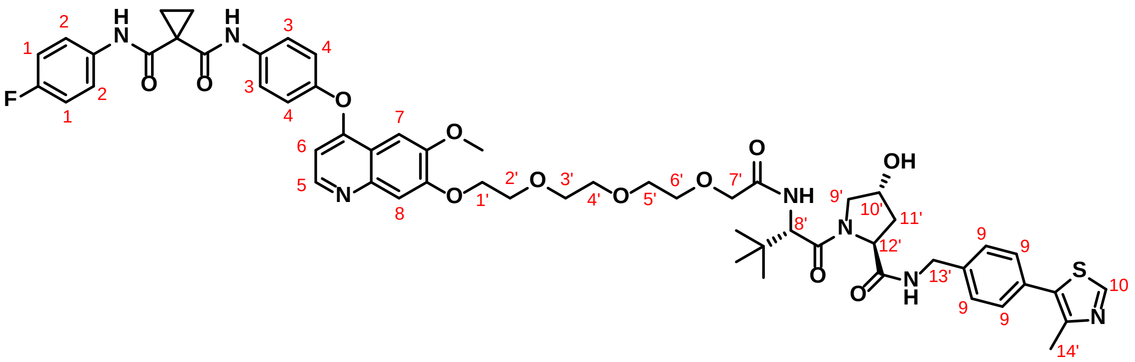

Eluent: chloroform-methanol (29:1 → 19:1). Pale-beige solid, yield 37%.

**M.p.** = 191 – 192 °C.
**$^{1}$H NMR** (400 MHz, $CDCl_3$): δ 9.62 (s, *NH*, 1H), 9.11 (s, *NH*, 1H), 8.65 (s, *C10-H*, 1H), 8.36 (d, *J* = 5.2 Hz, *C5-H*, 1H), 7.98-7.92 (m, *NH*, 1H), 7.60 (d, *J* = 8.8 Hz, *C3-H*, 2H), 7.53 – 7.41 (m, *C2-H*, *C7-H*, *C8-H*, 4H), 7.34-7.28 (m, *C9-H*, *NH*, 5H), 7.12 (d, *J* = 8.8 Hz, *C4-H*, 2H), 7.06-6.96 (m, *C1-H*, 2H), 6.40 (d, *J* = 5.3 Hz, *C6 -H*, 1H), 4.76 (t, *J* = 7.8 Hz, *C12'-H*, 1H), 4.57 – 4.45 (m, *C8'-H*, *C10'-H*, *C13'-H*, 3H), 4.43 – 4.22 (m, *C13'-H*, *C1'-H*, 3H), 4.06 – 3.87 (m, *OMe*, *C6'-H*, *C7'-H*, *C9'-H*, 8H), 3.79 – 3.53 (m, *C2'-H*, *C3'-H*, *C4'-H*, *C5'-H*, *C9'-H*, 9H), 2.47 (s, *C14'-H*, 3H), 2.43 – 2.37 (m, *C11'-H*, 1H), 2.16 – 2.08 (m, *C11'-H*, 1H), 1.73 – 1.59 (m, $C_{cyclopropane}$-*H*, 4H), 0.96 (s, *tBu*, 9H).
**$^{13}$C NMR** (100 MHz, $CDCl_3$): δ 171.42, 171.04, 170.36, 169.51, 169.10, 161.01, 159.79 (d, *J* = 244.7 Hz), 152.47, 150.78, 150.44, 149.92, 148.44, 148.26, 146.28, 138.37, 135.14, 133.36 (d, J = 3.1 Hz), 131.77, 130.82, 129.47 (2C), 128.13 (2C), 122.90 (d, *J* = 8.1 Hz, 2C), 122.62 (2C), 121.69 (2C), 116.21, 115.80 (d, *J* = 22.6 Hz, 2C), 108.27, 103.36, 99.67, 71.17, 70.79, 70.70, 70.48, 70.38, 70.19, 69.37, 68.39, 58.77, 57.05, 56.95, 56.16, 43.23, 36.60, 35.42, 29.33, 26.50 (3C), 17.67, 16.14 (2C).
**$^{19}$F NMR** (376 MHz, $CDCl_3$): -121.03 – -121.16 (m).
**Elemental analysis**: for $C_{57}H_{64}FN_7O_{12}S$ calcld.: C, 62.80; H, 5.92; found: C, 62.92; H, 6.03.
**HRMS (ESI):** m/z $[M+H]^+$ for $C_{57}H_{64}FN_7O_{12}S$ calcld.: 1090.4390; found: 1090.4264.

***N*-(4-fluorophenyl)-*N*-(4-((7-(((*S*)-3-((2*S*,4*R*)-4-hydroxy-2-((4-(4-methylthiazol-5-yl)benzyl)carbamoyl)pyrrolidine-1-carbonyl)-2,2-dimethyl-5,8,19-trioxo-12,15-dioxa-4,9,18-triazatricosan-23-yl)oxy)-6-methoxyquinolin-4-yl)oxy)phenyl)cyclopropane-1,1-dicarboxamide 15b**

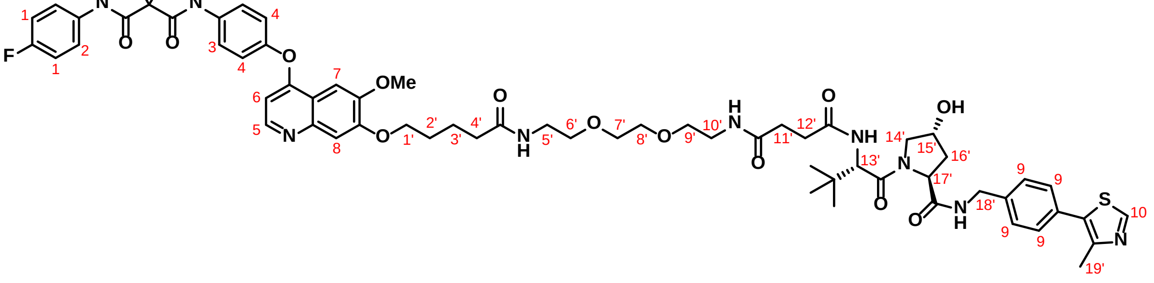

Eluent: ethyl acetate-methanol (9:1 → 1:1). Pale-yellow powder, yield 43%.

**M.p.** = 183 – 184 °C.

**$^{1}$H NMR** (400 MHz, DMSO-*d*$_6$): δ 10.17 (s, *NH*, 1H), 10.05 (s, *N*H, 1H), 8.98 (s, *C10-H*, 1H), 8.55 (t, *J* = 6.1 Hz, *NH*, 1H), 8.45 (d, *J* = 5.2 Hz, *C5-H*, 1H), 7.92-7.84 (m, *NH*, 3H), 7.76 (d, *J* = 9.0 Hz, *C3-H*, 2H), 7.67 – 7.61 (m, *C2*-H, 2H), 7.50 (s, *C7*-H, 1H), 7.43 – 7.33 (m, *C8-H*, *C9-H*, 5H), 7.22 (d, *J* = 9.0 Hz, *C4*-H, 2H), 7.19 – 7.11 (m, *C1*-H, 2H), 6.42 (d, *J* = 5.2 Hz, *C6 -H*, 1H), 5.11 (d, *J* = 3.3 Hz, *OH*, 1H), 4.51 (d, *J* = 9.4 Hz, *C13'*-H, 1H), 4.46 – 4.38 (m, *C17'-H*, *C18'-H*, 2H), 4.36-4.31 (m, *C15'-H*, 1H), 4.22 (dd, *J* = 15.9, 5.5 Hz, *C18'-H*, 1H), 4.14 (t, *J* = 6.3 Hz, *C1'-H*, 2H), 3.93 (s, *OMe*, 3H), 3.69 – 3.59 (m, *C14'-H*, 2H), 3.53-3.46 (m, *C7'-H*, *C8'-H*, 4H), 3.44 – 3.35 (m, *C6'-H*, *C9'-H*, 4H), 3.25 – 3.14 (m, *C5'-H*, *C10'-H*, 4H), 2.48-2.45 (m, *C12'-H*, 2H), 2.44 (s, *C19'-H*, 3H), 2.40 – 2.25 (m, *C11'-H*, 2H), 2.18 (t, *J* = 7.2 Hz, *C4'-H*, 2H), 2.07 – 1.99 (m, *C16'-H*, 1H), 1.93 – 1.85 (m, *C16'-H*, 1H), 1.84 – 1.74 (m, *C2'-H*, 2H), 1.74 – 1.64 (m, m, *C3'-H*, 2H), 1.47 (s, C$_{cyclopropane}$-*H*, 4H), 0.92 (s, *tBu*, 9H).
**$^{13}$C NMR** (100 MHz, DMSO-d$_6$): δ 172.04, 171.90, 171.47, 171.24, 169.55, 168.14, 168.10, 159.45, 158.26 (d, *J* = 240.1 Hz), 151.85, 151.41, 149.48, 149.40, 148.75, 147.70, 146.41, 139.48, 136.37, 135.16 (d, *J* = 2.6 Hz), 131.14, 129.63 (2C), 128.62 (2C), 127.41, 122.40 (d, *J* = 7.8 Hz, 2C), 122.16 (2), 121.14 (2C), 115.04, 115.01 (d, *J* = 22.2 Hz, 2C), 108.45, 102.98, 99.13, 69.51, 69.17, 69.10, 68.87, 67.97, 58.69, 56.40, 56.29, 55.71, 54.90, 41.65, 38.55, 38.47, 37.92, 35.31, 34.87, 31.54, 30.89, 30.52, 27.99, 26.33 (3C), 21.97, 15.93, 15.39 (2C).
**$^{19}$F NMR** (376 MHz, DMSO-*d*$_6$): -118.99 – -119.09 (m).
**Elemental analysis**: for $C_{64}H_{76}FN_9O_{13}S$ calcld.: C, 62.47; H, 6.23; found: C, 62.40; H, 6.13.
**HRMS (ESI):** m/z [M+H]$^+$ for $C_{64}H_{76}FN_9O_{13}S$ calcld.: 1230.5340; found: 1230.5320.

***N*-(4-fluorophenyl)-*N*-(4-((7-(3-(3-(3-(((*S*)-1-((2*S*,4*R*)-4-hydroxy-2-((4-(4-methylthiazol-5-yl)benzyl)carbamoyl)pyrrolidin-1-yl)-3,3-dimethyl-1-oxobutan-2-yl)amino)-3-oxopropoxy)propoxy)propoxy)-6-methoxyquinolin-4-yl)oxy)phenyl)cyclopropane-1,1-dicarboxamide 15c**

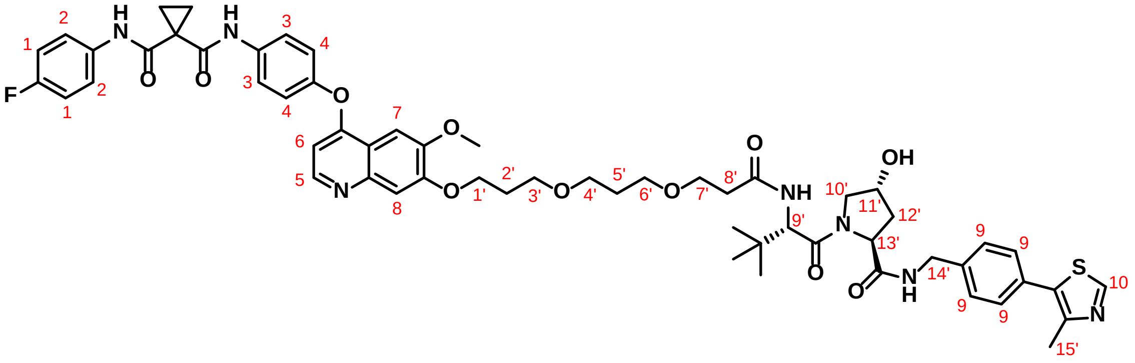

Eluent: chloroform-methanol (29:1 → 19:1). Pale-beige solid, yield 42%.

**M.p.** = 187 – 188 °C.
**$^{1}$H NMR** (400 MHz, CDCl$_3$): δ 9.62 (s, *NH*, 1H), 9.13 (s, *NH*, 1H), 8.65 (s, *C10-H*, 1H), 8.42 (d, *J* = 5.2 Hz, *C5-H*, 1H), 7.64-7.54 (m, *C3-H*, *NH*, 3H), 7.52 – 7.44 (m, *C2-H*, *C7-H*, 3H), 7.41 (s, *C8-H*, 1H), 7.35 – 7.27 (m, *C9-H*, 4H), 7.18 (d, *J* = 8.2 Hz, *NH*, 1H), 7.13 (d, *J* = 8.8 Hz, *C4-H*, 2H), 7.02 (t, J = 8.6 Hz, *C1-H*, 2H), 6.42 (d, *J* = 5.2 Hz, *C6 -H*, 1H), 4.73 (t, *J* = 7.8 Hz, *C13'-H*, 1H), 4.55 – 4.42 (m, *C9'-H*, *C11'-H*, *C14'-H*, 3H), 4.34 – 4.21 (m, *C1'-H*, *C14'-H*, 3H), 4.08 – 4.02 (m, *C10'-H*, 1H), 3.99 (s, *OMe*, 3H), 3.64 – 3.44 (m, *C3'-H*, *C4'-H*, *C6'-H*, *C7'-H*, *C10'-H*, 9H), 2.50 – 2.36 (m, *C8'-H*, *C12'-H*, *C15'-H*, 6H), 2.19 – 2.05 (m, *C2'-H*, *C12'-H*, 3H), 1.88 – 1.79 (m, *C5'-H*, 2H), 1.72 – 1.62 (m, *C$_{cyclopropane}$-H*, 4H), 0.92 (s, *tBu*, 9H)

**$^{13}$C NMR** (100 MHz, $CDCl_3$): δ 172.27, 171.82, 171.07, 169.47, 169.11, 160.83, 159.83 (d, *J* = 244.7 Hz), 152.51, 151.00, 150.42, 149.94, 148.66, 148.51, 146.73, 138.26, 135.03, 133.35 (d, *J* = 3.1 Hz), 131.74, 130.96, 129.57 (2C), 128.16 (2C), 122.90 (d, *J* = 8.1 Hz, 2C), 122.64 (2C), 121.66 (2C), 116.11, 115.83 (d, *J* = 22.5 Hz, 2C), 108.49, 103.50, 99.69, 70.11, 68.41, 67.67, 67.28, 66.77, 66.16, 58.57, 57 .78, 56.71, 56.25, 43.29, 36.76, 36.17, 35.02, 29.81, 29.39, 29.31, 26.53 (3C), 17 .70, 16.15 (2C).
**$^{19}$F NMR** (376 MHz, $CDCl_3$): -121.07 – -121.18 (m).
**Elemental analysis**: for $C_{58}H_{66}FN_7O_{11}S$ calcld.: C, 64.01; H, 6.11; found: C, 64.10; H, 6.21.
**HRMS (ESI):** m/z $[M+H]^+$ for $C_{58}H_{66}FN_7O_{11}S$ calcld.: 1088.4598; found: 1088.4491.

***N*-(4-fluorophenyl)-*N*-(4-((7-((11-(((*S*)-1-((2*S*,4*R*)-4-hydroxy-2-((4-(4-methylthiazol-5-yl)benzyl)carbamoyl)pyrrolidin-1-yl)-3,3-dimethyl-1-oxobutan-2-yl)amino)-11-oxoundecyl)oxy)-6-methoxyquinolin-4-yl)oxy)phenyl)cyclopropane-1,1-dicarboxamide 15d**

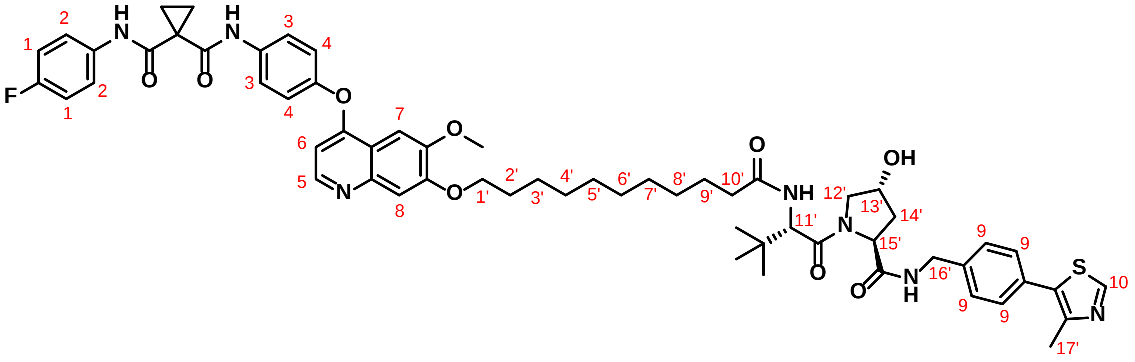

Eluent: chloroform-methanol (29:1 → 19:1). Pale-yellow solid, yield 61%.

**M.p.** = 176 – 177 °C.
**$^1$H NMR** (400 MHz, $CDCl_3$): δ 9.66 (s, *NH*, 1H), 9.20 (s, *NH*, 1H), 8.65 (s, *C10-H*, 1H), 8.24 (d, *J* = 5.2 Hz, *C5-H*, 1H), 7.61 (d, *J* = 8.8 Hz, *C3-H*, 2H), 7.51 (s, *C7-H*, 1H), 7.46 (dd, *J* = 8.9, 4.8 Hz, *C2-H*, 2H), 7.40 (t, *J* = 5.7 Hz, *NH*, 1H), 7.37-7.28 (m, *C8-H*, *C9-H*, 5H), 7.13 (d, *J* = 8.8 Hz, *C4-H*, 2H), 7.01 (t, *J* = 8.7 Hz, *C1-H*, 2H), 6.41 (d, *J* = 5.2 Hz, *C6 -H*, 1H), 6.23 (d, *J* = 8.7 Hz, *NH*, 1H), 4.69 (t, *J* = 7.8 Hz, *C15'-H*, 1H), 4.56 – 4.43 (m, *C11'-H*, *C13'-H*, *C16'-H*, 3H), 4.31 (dd, *J* = 15.0, 5.2 Hz, *C15'-H*, 1H), 4.14 (t, *J* = 6.7 Hz, *C1'-H*, 2H), 4.04 – 3.94 (m, *C12'-H*, *OMe*, 4H), 3.64 – 3.56 (m, *C12'-H*, 1H), 2.49 (s, *C17'-H*, 3H), 2.46 – 2.41 (m, *C14'-H*, 1H), 2.18 – 2.06 (m, *C14'-H*, *C10'-H*, 3H), 1.96 – 1.83 (m, *C2'-H*, 2H), 1.72 – 1.62 (m, *C3'-H*, 2H), 1.60 – 1.50 (m, *C4'-H*, 2H), 1.50 – 1.41 (m, *C9'-H*, 2H), 1.37-1.16 ( m, *C5'-H*, *C6'-H*, *C7'-H*, *C8'-H*, $C_{cyclopropane}$-*H*, 12H), 0.92 (s, *tBu*, 9H).
**$^{13}$C NMR** (100 MHz, $CDCl_3$): δ 173.81, 171.91, 170.97, 169.50, 169.10, 160.73, 159.80 (d, *J* = 244.6 Hz), 152.49, 151.02, 150.46, 149.92, 148.72, 148.50, 146.89, 138.14, 134.97, 133.34 (d, *J* = 3.0 Hz), 131.69, 131.01, 129.59 (2C), 128.14 (2C), 122.91 (d, *J* = 8.1 Hz, 2C), 122.61 (2C), 121.68 (2C), 115.99, 115.81 (d, *J* = 22.5 Hz, 2C), 108.44, 103.36, 99.60, 70.04, 69.08, 58.74, 57.49, 56.79, 56.25, 43.31, 36.62, 36.13, 35.20, 29.47, 29.39, 29.36 (2C), 29.29, 29.24, 28.84, 26.52 (3C), 25.98, 25.70, 17.61, 16.14 (2C).
**$^{19}$F NMR** (376 MHz, $CDCl_3$): -121.05 – -121.17 (m).
**Elemental analysis**: for $C_{60}H_{70}FN_7O_9S$ calcld.: C, 66.46; H, 6.51; found: C, 66.54; H, 6.60.
**HRMS (ESI):** m/z $[M+H]^+$ for $C_{60}H_{70}FN_7O_9S$ calcld.: 1084.5013; found: 1084.4855.

**N-(4-((7-((1-((2-(2,6-dioxopiperidin-3-yl)-1-oxoisoindolin-4-yl)amino)-1,4,15-trioxo-8,11-dioxa-5,14-diazanonadecan-19-yl)oxy)-6-methoxyquinolin-4-yl)oxy)phenyl)-N-(4-fluorophenyl)cyclopropane-1,1-dicarboxamide 16a**

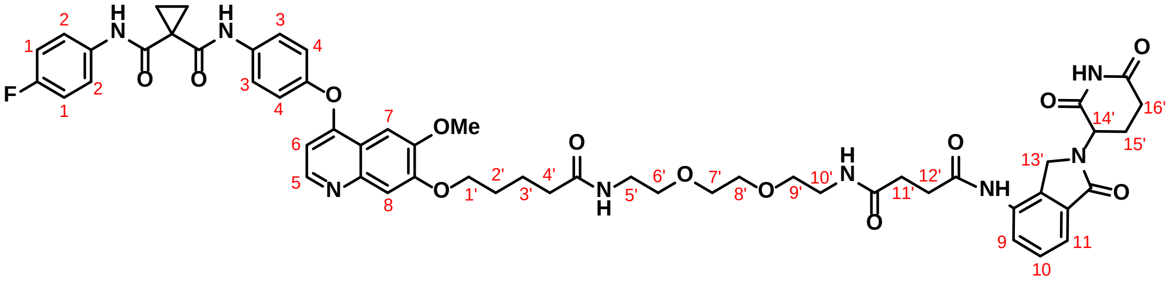

Eluent: ethyl acetate-methanol (9:1 → 7:3). White powder, yield 36%.

**M.p.** = 190 – 191 °C.
**$^1$H NMR** (400 MHz, DMSO-$d_6$): δ 11.02 (s, *NH*, 1H), 10.19 (s, *NH*, 1H), 10.07 (s, *NH*, 1H), 9.88 (s, *NH*, 1H), 8.45 (d, *J* = 5.2 Hz, *C5-H*, 1H), 7.95 (t, *J* = 5.5 Hz, *NH*, 1H), 7.90 (t, *J* = 5.5 Hz, *NH*, 1H), 7.82 (dd, *J* = 6.8, 2.1 Hz, *C10-H*, 1H), 7.76 (d, *J* = 8.9 Hz, *C3-H*, 2H), 7.67-7.61 (m, *C2-H*, 2H), 7.51 – 7.45 (m, *C7-H*, *C9-H*, *C11-H*, 3H), 7.38 (s, *C8 –H*, 1H), 7.22 (d, *J* = 9.0 Hz, *C4-H*, 2H), 7.18 – 7.11 (m, *C1-H*, 2H), 6.42 (d, *J* = 5.2 Hz, *C6 -H*, 1H), 5.14 (dd, *J* = 13.3, 5.1 Hz, *C14'-H*, 1H), 4.35 (q, *J* = 17.6 Hz, *C13'-H*, 2H), 4.14 (t, *J* = 6.3 Hz, *C1'-H*, 2H), 3.93 (s, *OMe*, 3 H), 3.51-3.47 (m, *C7'-H*, *C8'-H*, 4H), 3.44-3.37 (m, *C6'-H*, *C9'-H*, 4H), 3.20 (p, *J* = 5.7 Hz, *C10'-H*, *C5'-H*, 4H), 2.97 – 2.86 (m, *C16'-H*, 1H), 2.66 – 2.55 (m, *C11'-H*, *C16'-H*, 3H), 2.43 (t, *J* = 7.1 Hz, *C12'-H*, 2H), 2.38 – 2.24 (m, *C15'-H*, 1H), 2.18 (t, *J* = 7.2 Hz, *C4'-H*, 2H), 2.06 – 1.99 (m, *C15'-H*, 1H), 1.84 – 1.74 (m, *C2'-H*, 2H), 1.74 – 1.64 (m, *C3'-H*, 2H), 1.48 (s, $C_{cyclopropane}$-*H*, 4H).
**$^{13}$C NMR** (101 MHz, DMSO-$d_6$): δ 172.83, 172.05, 171.26, 171.03, 170.68, 168.17, 168.12, 167.83, 159.92, 158.26 (d, *J* = 240.1 Hz), 151.85, 149.49, 149.40, 148.77, 146.43, 136.38, 135.17 (d, *J* = 2.6 Hz), 133.82, 133.49, 132.62, 128.59, 124.97, 122.41 (d, *J* = 7.9 Hz, 2C), 122.16 (2C), 121.14 (2C), 118.84, 115.04, 115.01 (d, *J* = 22.2 Hz, 2C), 108.47, 102.98, 99.13, 71.76, 69.51, 69.16, 69.11, 67.97, 55.72, 51.50, 46.43, 38.58, 38.47, 34.87, 31.57, 31.19, 30.69, 30.31, 27.99, 22.68, 21.97, 15.39 (2C).
**$^{19}$F NMR** (376 MHz, DMSO-$d_6$): -119.00 – -119.09 (m).
**Elemental analysis**: for $C_{55}H_{59}FN_8O_{13}$ calcld.: C, 62.37; H, 5.62; found: C, 62.30; H, 5.70.
**HRMS (ESI):** m/z $[M+H]^+$ for $C_{55}H_{59}FN_8O_{13}$ calcld.: 1059.4258; found: 1059.4221.

***N*-(4-((7-((5-((6-((2-(2,6-dioxopiperidin-3-yl)-1-oxoisoindolin-4-yl)amino)-6-oxohexyl)amino)-5-oxopentyl)oxy)-6-methoxyquinolin-4-yl)oxy)phenyl)-*N*-(4-fluorophenyl)cyclopropane-1,1-dicarboxamide 16b**

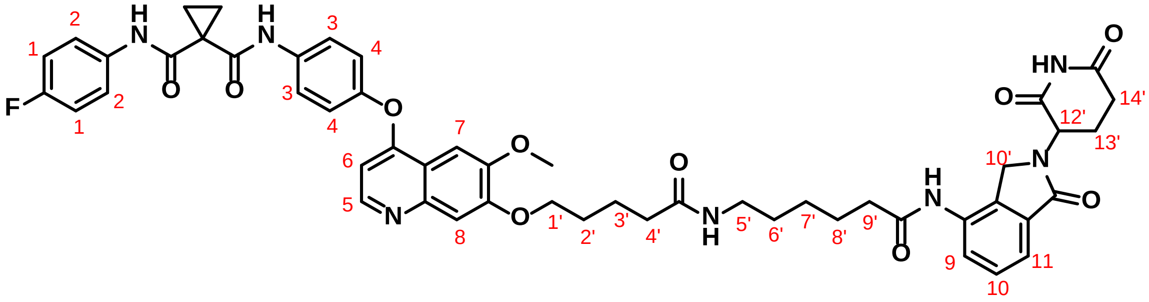

Eluent: ethyl acetate-methanol (1:1 → 7:3). Pale-beige solid, yield 57%.

**M.p.** = 183 – 184 °C.
**$^1$H NMR** (400 MHz, DMSO-*d$_6$*): δ 11.01 (s, *NH*, 1H), 10.18 (s, *NH*, 1H), 10.05 (s, *NH*, 1H), 9.78 (s, *NH*, 1H), 8.45 (d, *J* = 5.2 Hz, *C5-H*, 1H), 7.84 – 7.79 (m, *C10-H*, *NH*, 2H), 7.76 (d, *J* = 9.0 Hz, *C3-H*, 2H), 7.67-7.61 (m, *C2-H*, 2H), 7.51 – 7.46 (m, *C7-H*, *C9-H*, *C11-H*, 3H), 7.37 (s, *C8 –H*, 1H) 7.22 (d, *J* = 9.0 Hz, *C4-H*, 2H), 7.18 – 7.12 (m, *C1-H*, 2H), 6.42 (d, *J* = 5.2 Hz, *C6 -H*, 1H), 5.13 (dd, *J* = 13.1, 5.0 Hz, *C12'-H*, 1H), 4.36 (q, *J* = 17.4 Hz, *C10'-H*, 2H), 4.13 (t, *J* = 6.3 Hz, *C1'-H*, 2H), 3.93 (s, *OMe*, 3 H), 3.05 (dd, *J* = 13.0, 6.8 Hz, *C5'-H*, 2H), 2.96 – 2.85 (m, *C14'-H*, 1H), 2.64 – 2.56 (m, *C14'-H*, 1H), 2.39 – 2.32 (m, *C9'-H*, *C13'-H*, 3H), 2.15 (t, *J* = 7.3 Hz, *C4'-H*, 2H), 2.06 – 1.99 (m, *C13'-H*, 1H), 1.83-1.74 (m, *C2'-H*, 2H), 1.69 (dd, *J* = 14.9, 7.5 Hz, *C3'-H*, 2H), 1.65-1.56 (m, *C8'-H*, 2H), 1.48 (s, $C_{cyclopropane}$-*H*, 4H), 1.46 – 1.40 (m, *C6'-H*, 2H), 1.35-1.28 (m, *C7'-H*, 2H).
**$^{13}$C NMR** (101 MHz, DMSO-*d$_6$*): δ 172.84, 171.70, 171.30, 171.05, 168.16, 168.12, 167.81, 159.93, 158.26 (d, *J* = 240.0 Hz), 151.84, 149.49, 149.40, 148.78, 146.43, 136.38, 135.17 (d, *J* = 2.7 Hz), 133.80, 133.67, 132.64, 128.58, 125.22, 122.41 (d, *J* = 7.7 Hz, 2C), 122.16 (2C), 121.14 (2C), 118.93, 115.04, 115.01 (d, *J* = 22.2 Hz, 2C), 108.48, 102.98, 99.14, 67.96, 55.72, 51.54, 46.50, 38.34, 35.74, 35.01, 31.56, 31.20, 29.01, 28.04, 26.12, 24.83, 22.02, 20.76, 15.39 (2C).
**$^{19}$F NMR** (376 MHz, DMSO-*d$_6$*): -119.01 – -119.10 (m).
**Elemental analysis**: for $C_{51}H_{52}FN_7O_{10}$ calcld.: C, 65.03; H, 5.56; found: C, 65.13; H, 5.43.
**HRMS (ESI):** m/z $[M+H]^+$ for $C_{51}H_{52}FN_7O_{10}$ calcld.: 942.3832; found: 942.3470.

**N-(4-((7-((1-(3-((6-((2-(2,6-dioxopiperidin-3-yl)-1-oxoisoindolin-4-yl)amino)-6-oxohexyl)amino)-3-oxopropyl)-1H-1,2,3-triazol-4-yl)methoxy)-6-methoxyquinolin-4-yl)oxy)phenyl)-N-(4-fluorophenyl)cyclopropane-1,1-dicarboxamide 17a**

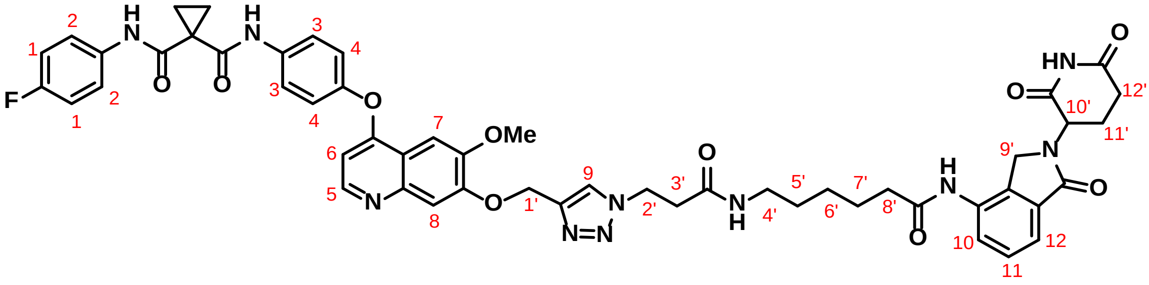

Eluent: ethyl acetate-methanol (9:1 → 7:3). White solid, yield 48%.

**M.p.** = 173 – 174 °C.
**$^1$H NMR** (400 MHz, DMSO-*d$_6$*): δ 11.01 (s, *NH*, 1H), 10.20 (s, *NH*, 1H), 10.08 (s, *NH*, 1H), 9.83 (s, *NH*, 1H), 8.47 (d, *J* = 5.2 Hz, *C5-H*, 1H), 8.28 (s, *C9-H*, 1H), 8.04 – 7.97 (m, *NH*, 1H), 7.83-7.79 (m, *C11-H*, 1H), 7.76 (d, *J* = 9.0 Hz, *C3-H*, 2H), 7.67 – 7.61 (m, *C2-H*, *C8-H*, 3H), 7.52-7.44 (*C7-H*, *C10-H*, *C12-H*, 3H), 7.22 (d, *J* = 8.9 Hz, *C4-H*, 2H), 7.18 – 7.11 (m, *C1-H*, 2H), 6.43 (d, *J* = 5.2 Hz, *C6 -H*, 1H), 5.31 (s, *C1'-H*, 2H ), 5.13 (dd, *J* = 13.3, 5.1 Hz, *C10'-H*, 1H), 4.59 (t, *J* = 6.7 Hz, *C2'-H*, 2H), 4.36 (q, *J* = 17.5 Hz, *C9'-H*, 2H), 3.90 (s, *OMe*, 3H), 3.02 (dd, *J* = 12.7, 6.6 Hz, *C4'-H*, 2H), 2.96 – 2.85 (m, *C12'-H*, 1H), 2.72 (t, *J* = 6.8 Hz, *C3'-H*, 2H), 2.64 – 2.54 (m, *C12'-H*, 1H), 2.40 – 2.30 (m, *C8'-H*, *C11'-H*, 3H), 2.05 – 2.00 (m, *C11'-H*, 1H), 1.63 – 1.53 (m, *C6'-H*, 2H), 1.48 (s, $C_{cyclopropane}$-*H*, 4H), 1.43 – 1.34 (m, *C5'-H*, 2H), 1.31 – 1.21 (m, *C7'-H*, 2H).
**$^{13}$C NMR** (101 MHz, DMSO-*d$_6$*): δ 172.88, 171.35, 171.06, 168.72, 168.21, 168.16, 167.85, 159.96, 158.28 (d, *J* = 240.2 Hz), 151.20, 149.48, 149.34, 148.87, 146.28, 141.87, 136.41, 135.18 (d, *J* = 2.7 Hz), 133.82, 133.70, 132.65, 128.59, 125.29, 125.12, , 122.44 (d, *J* = 7.9 Hz, 2C),

122.20 (2C), 121.17 (2C), 118.95, 115.34, 115.03 (d, *J* = 22.2 Hz, 2C), 109.13, 103.13, 99.26, 66.36, 55.68, 51.56, 46.56, 45.96, 38.43, 35.73, 35.48, 31.59, 31.22, 28.79, 26.04, 24.79, 22.62, 15.41 (2C).
**$^{19}$F NMR** (376 MHz, DMSO-*d6*): δ -118.79 – -118.91 (m).
**Elemental analysis**: for $C_{52}H_{51}FN_{10}O_{10}$ calcld.: C, 62.77; H, 5.17; found: C, C, 62.84; H, 5.10.
**HRMS (ESI):** m/z [M+H]$^+$ for $C_{52}H_{51}FN_{10}O_{10}$ calcld.: 995.3846; found: 995.3833.

***N*-(4-((7-(3-(1-(3-((6-((2-(2,6-dioxopiperidin-3-yl)-1-oxoisoindolin-4-yl)amino)-6-oxohexyl)amino)-3-oxopropyl)-1H-1,2,3-triazol-4-yl)propoxy)-6-methoxyquinolin-4-yl)oxy)phenyl)-*N*-(4-fluorophenyl)cyclopropane-1,1-dicarboxamide 17b**

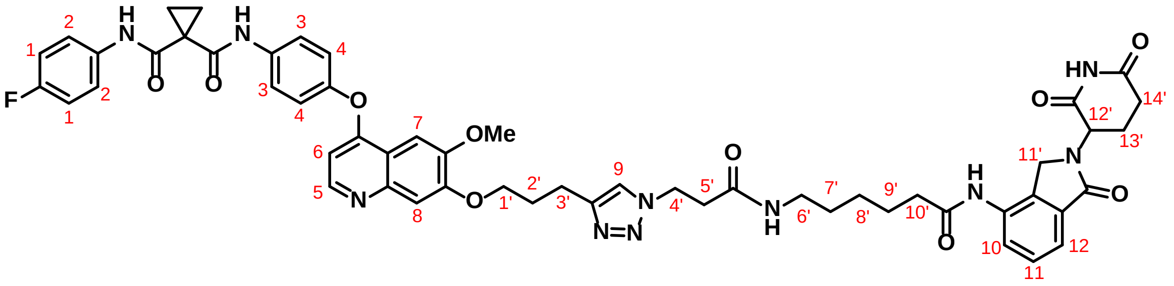


Eluent: ethyl acetate-methanol (9:1 → 7:3). White powder, yield 45%.

**M.p.** = 175 – 176 °C.
**$^1$H NMR** (400 MHz, DMSO-*d6*): δ 11.02 (s, *NH*, 1H), 10.20 (s, *NH*, 1H), 10.07 (s, *NH*, 1H), 9.81 (s, *NH*, 1H), 8.45 (d, *J* = 5.2 Hz, *C5-H*, 1H), 7.97 (t, *J* = 6.6 Hz, *NH*, 1H), 7.84 – 7.79 (m, *C9-H*, *C11-H*, 2H) 7.76 (d, *J* = 9.0 Hz, *C3-H*, 2H), 7.67 – 7.62 (m, *C2-H*, 2H), 7.52-7.47 (*C7-H*, *C10-H*, *C12-H*, 3H), 7.37 (s, *C8-H*, 1H), 7.24-7.20 (m, *C4-H*, 2H), 7.18 – 7.11 (m, *C1-H*, 2H), 6.42 (d, *J* = 5.2 Hz, *C6 -H*, 1H), 5.13 (dd, *J* = 13.3, 5.1 Hz, *C12'-H*, 1H), 4.51 (t, *J* = 6.9 Hz, *C4'-H*, 2H), 4.36 (q, *J* = 17.5 Hz, *C11'-H*, 2H), 4.19 (t, *J* = 6.3 Hz, *C1'-H*, 2H), 3.94 (s, *OMe*, 3H), 3.01 (dd, *J* = 12.6, 6.7 Hz, *C6'-H*, 2H), 2.96 – 2.85 (m, , *C14'-H*, 1H), 2.82 (t, *J* = 7.5 Hz, *C3'-H*, 2H), 2.67 (t, *J* = 6.9 Hz, *C5'-H*, 2H), 2.64 – 2.56 (m, *C14'-H*, 1H), 2.38 – 2.30 (m, *C10'-H*, *C13'-H*, 3H), 2.18 – 2.09 (m, *C2'-H*, 2H), 2.05 – 2.00 (m, *C13'-H*, 1H), 1.62 – 1.52 (m, *C8'-H* 2H), 1.48 (s, $C_{cyclopropane}$-*H*, 4H), 1.37 (dt, *J* = 14.3, 7.1 Hz, *C7'-H*, 2H), 1.25 (dd, *J* = 15.1, 8.1 Hz, *C9'-H*, 2H).
**$^{13}$C NMR** (101 MHz, DMSO-*d6*): δ 172.84, 171.30, 171.05, 168.76, 168.17, 168.13, 167.82, 159.93, 158.26 (d, *J* = 240.2 Hz), 151.79, 149.48, 149.41, 148.79, 146.40, 145.87, 136.39, 135.17 (d, *J* = 2.5 Hz), 133.81, 133.68, 132.64, 128.56, 125.23, 122.40 (d, *J* = 7.8 Hz, 2C), 122.16 (2C), 121.14 (2C), 118.92, 118.85, 115.10, 115.01 (d, *J* = 22.2 Hz, 2C), 108.55, 103.01, 99.19, 67.50, 55.74, 51.53, 46.52, 45.78, 38.38, 35.69, 31.57, 31.20, 28.81, 28.33, 25.99, 24.76, 22.62 (2C), 21.62, 15.39 (2C).
**$^{19}$F NMR** (376 MHz, DMSO-*d6*): δ -119.00 – -119.10 (m).
**Elemental analysis**: for $C_{54}H_{55}FN_{10}O_{10}$ calcld.: C, 63.40; H, 5.42; found: C, 63.45; H, 5.36.
**HRMS (ESI):** m/z [M+H]$^+$ for $C_{54}H_{55}FN_{10}O_{10}$ calcld.: 1023.4159; found: 1023.4166.

**N-(4-((7-(4-(1-(3-((6-((2-(2,6-dioxopiperidin-3-yl)-1-oxoisoindolin-4-yl)amino)-6-oxohexyl)amino)-3-oxopropyl)-1H-1,2,3-triazol-4-yl)butoxy)-6-methoxyquinolin-4-yl)oxy)phenyl)-N-(4-fluorophenyl)cyclopropane-1,1-dicarboxamide 17c**

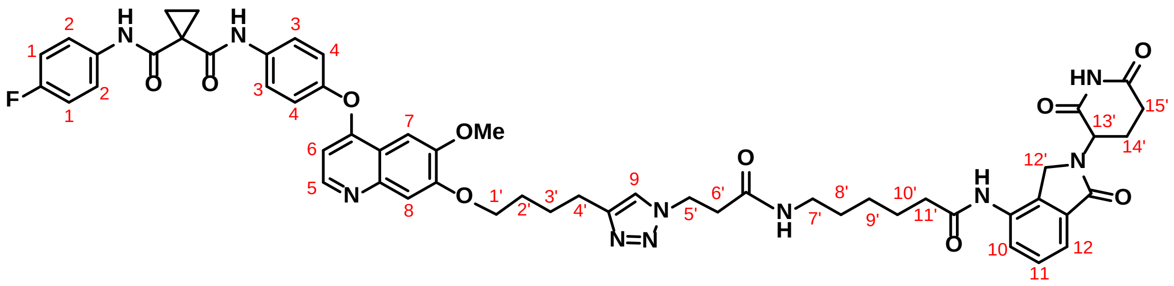

Eluent: ethyl acetate-methanol (9:1 → 7:3). Pale-yellow solid, yield 87%.

**M.p.** = 179 – 180 °C.
**$^{1}$H NMR** (400 MHz, DMSO-*d$_6$*): δ 11.02 (s, *NH*, 1H), 10.18 (s, *NH*, 1H), 10.05 (s, *NH*, 1H), 9.77 (s, *NH*, 1H), 8.45 (d, *J* = 5.2 Hz, *C5-H*, 1H), 7.95 (t, *J* = 5.5 Hz, *NH*, 1H), 7.82 – 7.74 (m, *C3-H*, *C9-H*, *C11-H*, 4H), 7.67 – 7.62 (m, *C2-H*, 2H), 7.51-7.44 (m, *C7-H*, *C10-H*, *C12-H*, 3H), 7.38 (s, *C8-H*, 1H), 7.22 (d, *J* = 9.0 Hz, *C4-H*, 2H), 7.19– 7.11 (m, *C1-H*, 2H), 6.41 (d, *J* = 5.2 Hz, *C6 -H*, 1H), 5.14 (dd, *J* = 13.4, 5.1 Hz, *C13'-H*, 1H), 4.50 (t, *J* = 6.8 Hz, C5*'-H*, 2H), 4.36 (q, *J* = 17.5 Hz, *C12'-H*, 2H), 4.16 (t, *J* = 6.0 Hz, *C1'-H*, 2H), 3.94 (s, *OMe*, 3H), 3.03 (dd, *J* = 12.7, 6.6 Hz, *C7'-H*, 2H), 2.96 – 2.85 (m, *C15'-H*, 1H), 2.73-2.64 (m, *C4'-H*, *C6'-H*, 4H), 2.64 – 2.56 (m, *C15'-H*, 1H), 2.38 – 2.30 (m, *C11'-H*, *C14'-H*, 3H), 2.06 – 2.01 (m, *C14'-H*, 1H), 1.88 – 1.75 (m, *C2'-H*, *C3'-H*, 4H), 1.62 – 1.53 (m, *C9'-H*, 2H), 1.48 (s, C$_{cyclopropane}$*-H*, 4H), 1.42 – 1.33 (m, *C8'-H*, 2H), 1.30 – 1.22 (m, *C10'-H*, 2H).
**$^{13}$C NMR** (101 MHz, DMSO-*d$_6$*): δ 172.83, 171.26, 171.05, 168.77, 168.15, 168.10, 167.80, 159.92, 158.25 (d, *J* =240.1 Hz), 151.85, 149.48, 149.39, 148.77, 146.42, 146.38, 136.37, 135.16 (d, *J* = 2.6 Hz), 133.78, 133.67, 132.63, 128.56, 125.22, 122.40 (d, *J* = 7.8 Hz, 2C), 122.15 (2C), 122.00, 121.14 (2C), 118.93, 115.04, 115.01 (d, *J* = 22.2 Hz, 2C), 108.47, 102.98, 99.12, 68.02, 55.71, 51.52, 46.48, 38.37, 35.70 (2C), 31.55, 31.20, 28.82, 27.97, 25.99, 25.60, 24.76, 24.63, 22.62 (2C), 15.38 (2C).
**$^{19}$F NMR** (376 MHz, DMSO-*d$_6$*): δ -119.00 – -119.11 (m).
**Elemental analysis**: for $C_{55}H_{57}FN_{10}O_{10}$ calcld.: C, 63.70; H, 5.54; found: C, 63.65; H, 5.62.
**HRMS (ESI):** m/z $[M+H]^+$ for $C_{55}H_{57}FN_{10}O_{10}$ calcld.: 1037.4316; found: 1037.4320.

**N-(4-((7-((9-(1-(3-((6-((2-(2,6-dioxopiperidin-3-yl)-1-oxoisoindolin-4-yl)amino)-6-oxohexyl)amino)-3-oxopropyl)-1H-1,2,3-triazol-4-yl)nonyl)oxy)-6-methoxyquinolin-4-yl)oxy)phenyl)-N-(4-fluorophenyl)cyclopropane-1,1-dicarboxamide 17d**

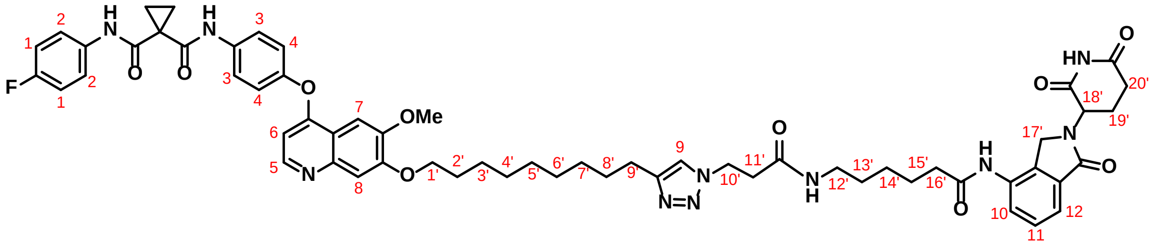

Eluent: ethyl acetate-methanol (9:1 → 7:3). Pale-yellow powder, yield 42%.

**M.p.** = 185 – 186 °C.
**$^{1}$H NMR** (400 MHz, DMSO-*d$_6$*): δ 11.02 (s, *NH*, 1H), 10.20 (s, *NH*, 1H), 10.08 (s, *NH*, 1H), 9.83 (s, *NH*, 1H), 8.45 (d, *J* = 5.2 Hz, *C5-H*, 1H), 7.97 (t, *J* = 6.5 Hz, *NH*, 1H), 7.82 (dd, *J* = 7.1, 1.8 Hz, *C11*, 1H), 7.76 (d, *J* = 9.0 Hz, *C3-H*, 2H), 7.70 (s, *C9-H*, 1H), 7.67 – 7.62 (m, *C2-H*, 2H),

7.51-7.46 (*C7-H*, *C10-H*, *C12-H*, 3H), 7.37 (s, *C8-H*, 1H), 7.22 (d, *J* = 9.0 Hz, *C4-H*, 2H), 7.18 – 7.12 (m, *C1-H*, 2H), 6.41 (d, *J* = 5.2 Hz, *C6 -H*, 1H), 5.14 (dd, *J* = 13.3, 5.1 Hz, *C18'-H*, 1H), 4.49 (t, *J* = 6.8 Hz, *C10'-H*, 2H), 4.37 (q, *J* = 17.6 Hz, *C17'-H*, 2H), 4.12 (t, *J* = 6.4 Hz, *C1'-H*, 2H), 3.92 (s, *OMe*, 3H), 3.02 (dd, *J* = 12.6, 6.7 Hz, *C12'-H*, 2H), 2.96-2.85 (m, *C20'-H*, 1H ), 2.68 – 2.61 (m, *J* = 13.9, 7.0 Hz, *C11'-H, C20'-H*, 3H), 2.59 – 2.54 (m, *C9'-H*, 2H), 2.38 – 2.31 (m, *C16'-H*, *C19'-H*, 3H), 2.05 – 1.99 (m, *C19'-H*, 1H), 1.83 – 1.77 (m, *C2'-H*, 2H), 1.62 – 1.53 (m, *C8'-H, C15'-H*, 4H), 1.50-1.24 (m, *C3'-H, C4'-H, C5'-H, C6'-H, C7'-H, C13'-H, C14'-H*, $C_{cyclopropane}$-*H*, 18H).

**$^{13}$C NMR** (101 MHz, DMSO-$d_6$): δ 172.84, 171.29, 171.04, 168.78, 168.18, 168.14, 167.82, 159.92, 158.25 (d, J = 239.9 Hz), 151.90, 149.48, 149.43, 148.75, 146.65, 146.45, 136.39, 135.18 (d, J = 2.4 Hz), 133.83, 133.64, 132.64, 128.55, 125.19, 122.40 (d, *J* = 7.7 Hz, 2C), 122.16 (2C), 121.79, 121.13 (2C), 118.90, 115.02, 115.01 (d, *J* = 22.1 Hz, 2C), 108.46, 102.98, 99.13, 68.27, 55.71, 51.53, 46.53, 45.68, 38.36, 35.69 (2C), 31.57, 31.20, 29.02, 28.94, 28.82, 28.76 (2C), 28.58, 28.46, 26.00, 24.97, 24.78, 22.63 (2C), 15.38 (2C).

**$^{19}$F NMR** (376 MHz, DMSO-$d_6$): -119.02 – -119.13 (m).

**Elemental analysis**: for $C_{60}H_{67}FN_{10}O_{10}$ calcld.: C, 65.09; H, 6.10; found: C, 64.98; H, 6.15.

**HRMS (ESI):** m/z $[M+H]^+$ for $C_{60}H_{67}FN_{10}O_{10}$ calcld.: 1107.5098; found: 1107.5064.

# $^{1}H$ and $^{13}C$ NMR spectra

Compound **4a**

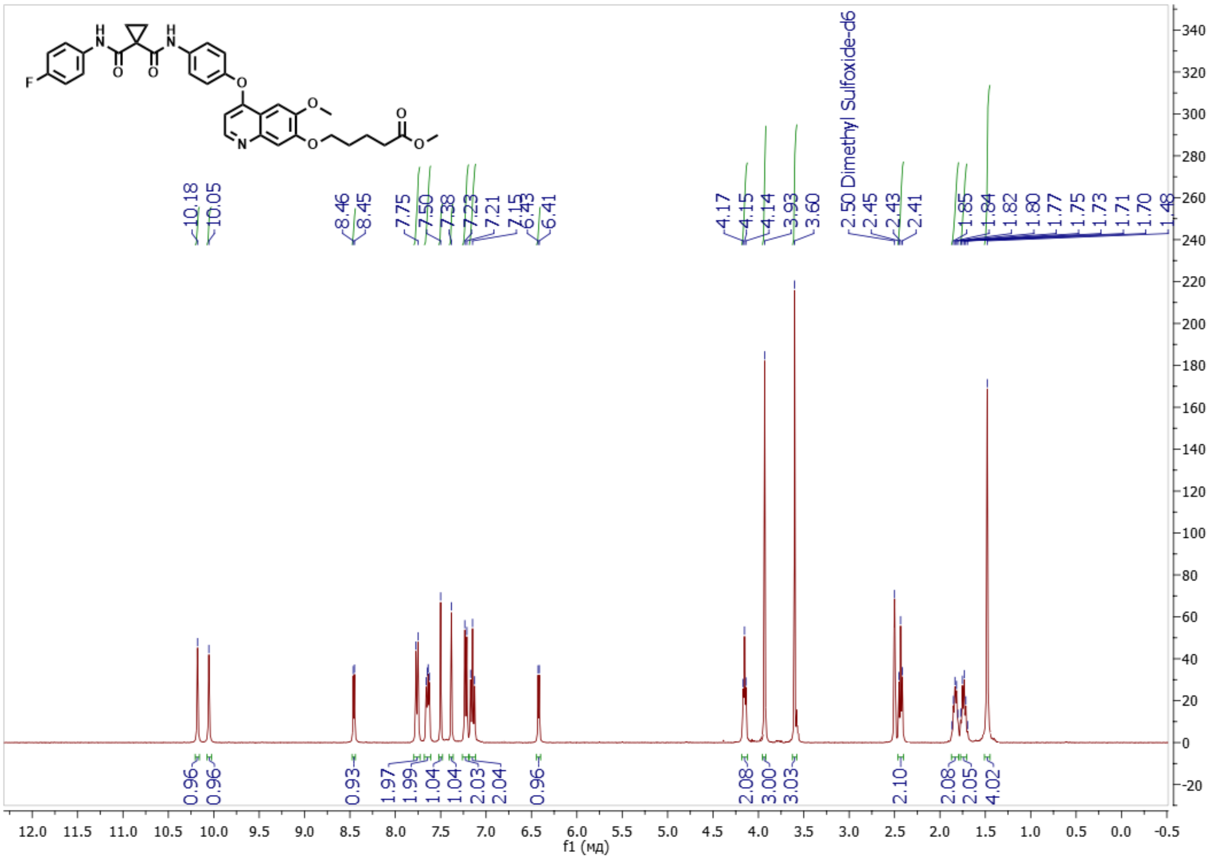

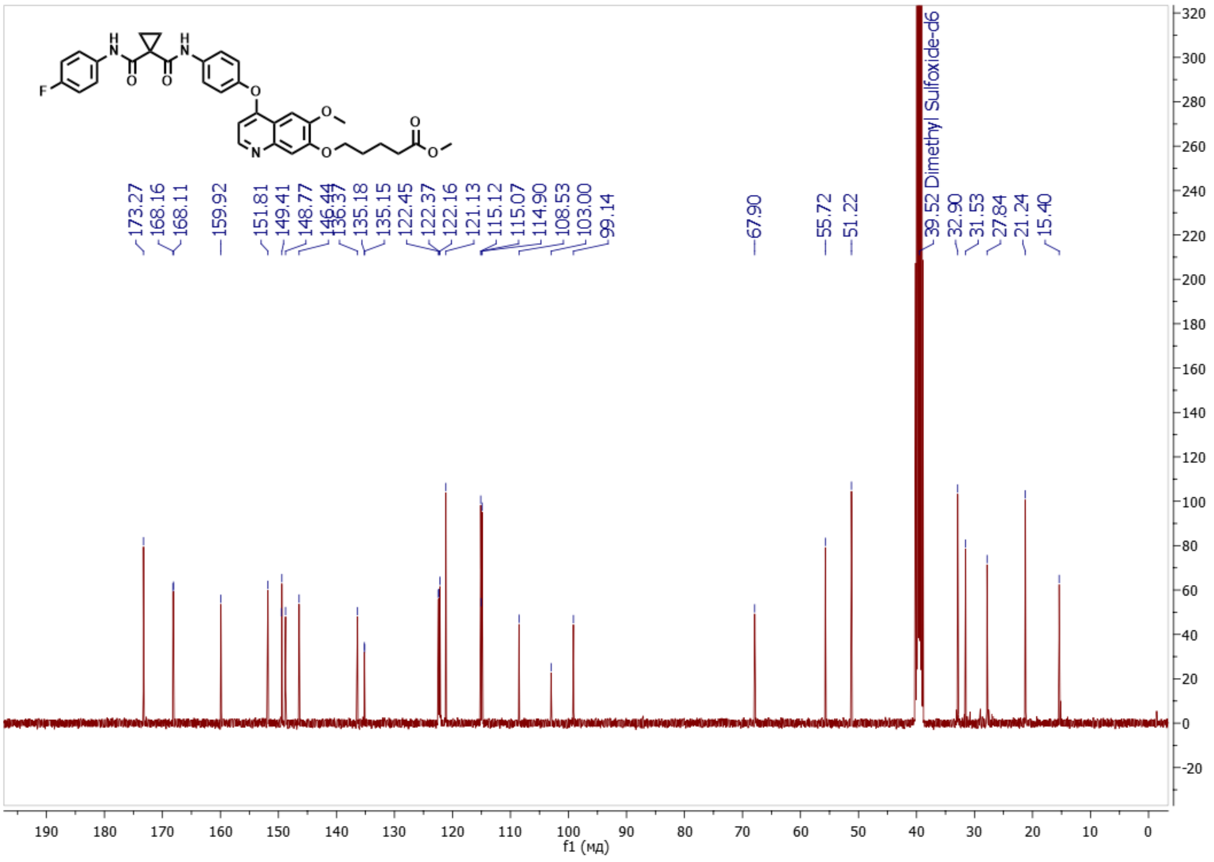

Compound **4b**

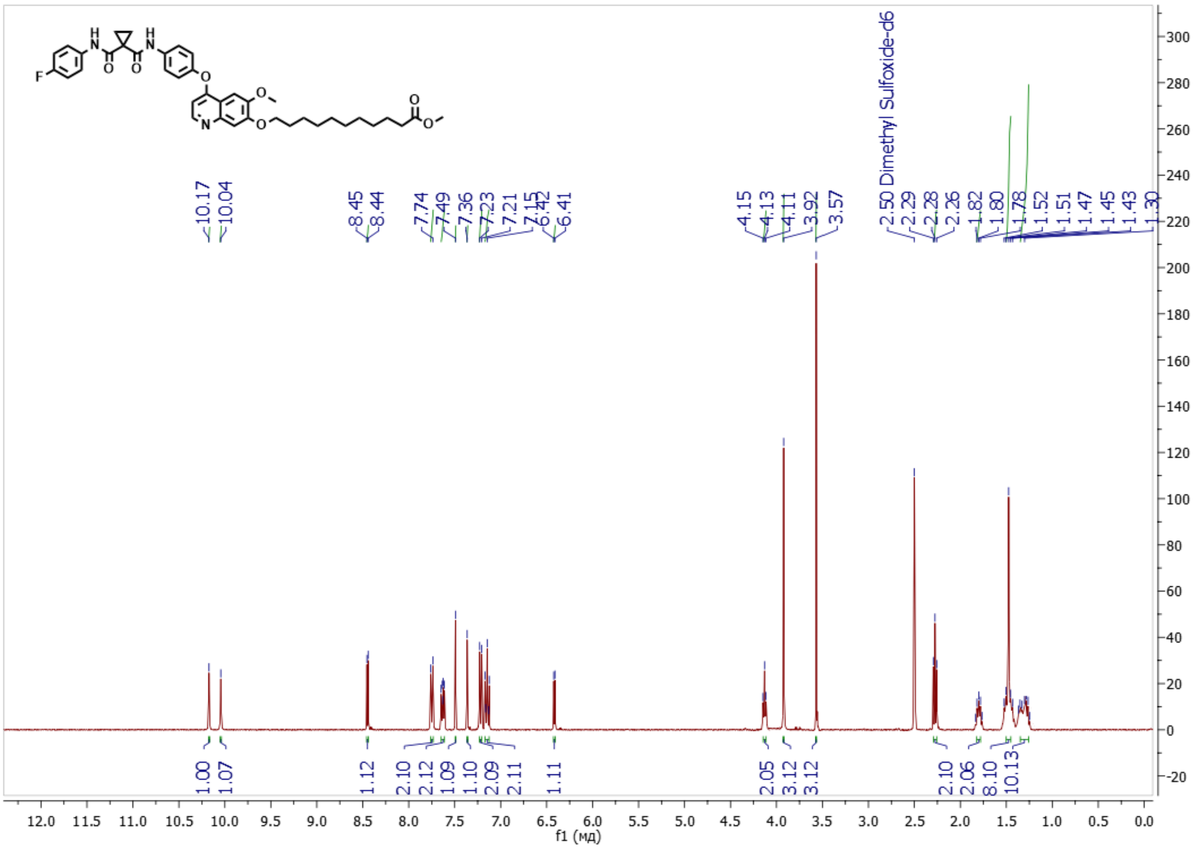
10.17
10.04
8.45
8.44
7.74
7.49
7.36
7.23
7.21
7.15
6.42
6.41
4.15
4.13
4.11
3.92
3.57
2.50 Dimethyl Sulfoxide-d6
2.29
2.28
2.26
1.82
1.80
1.78
1.52
1.51
1.47
1.45
1.43
1.30
1.00
1.07
1.12
2.10
2.12
1.09
1.10
2.09
2.11
1.11
2.05
3.12
3.12
2.10
2.06
8.10
10.13
f1 (мд)

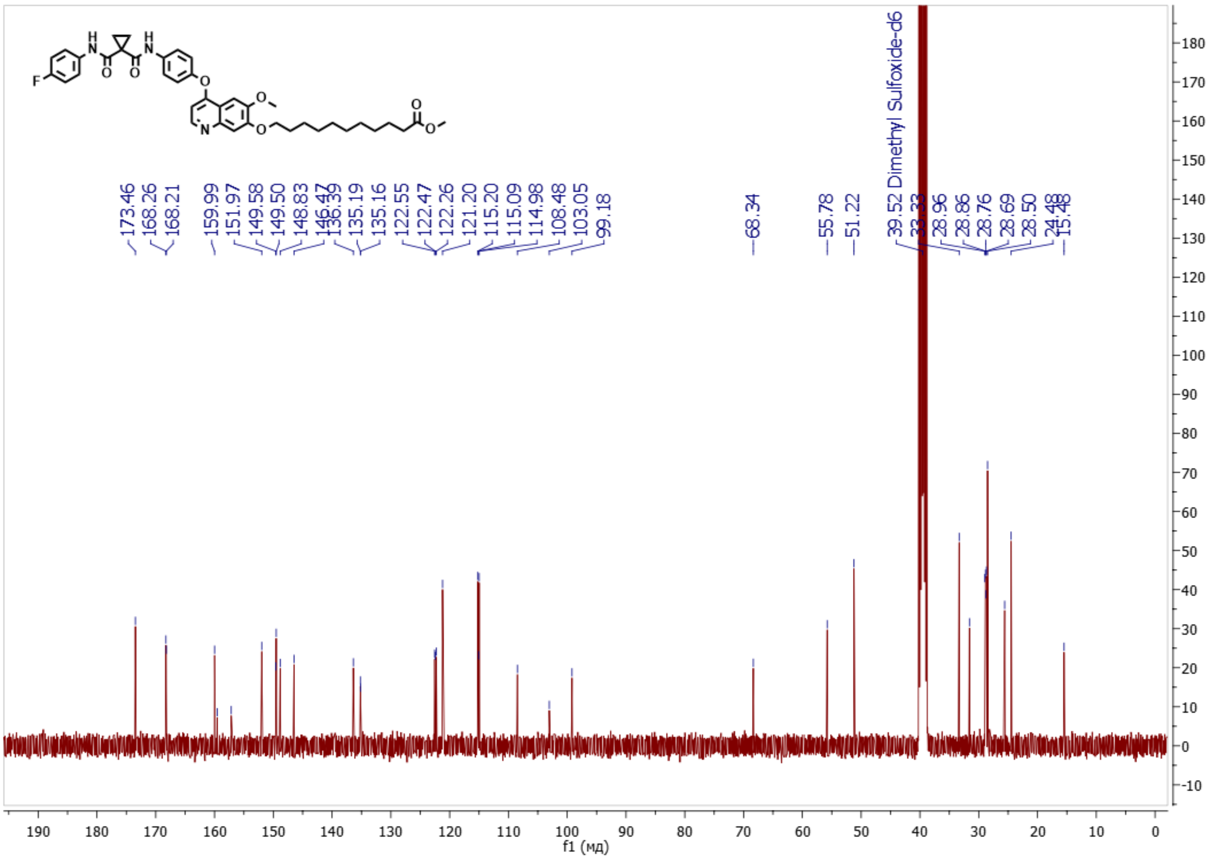
173.46
168.26
168.21
159.99
151.97
149.58
149.50
148.83
146.47
136.39
135.19
135.16
122.55
122.47
122.26
121.20
115.20
115.09
114.98
108.48
103.05
99.18
68.34
55.78
51.22
39.52 Dimethyl Sulfoxide-d6
33.33
28.96
28.86
28.76
28.69
28.50
24.48
15.48
f1 (мд)

Compound **5a**

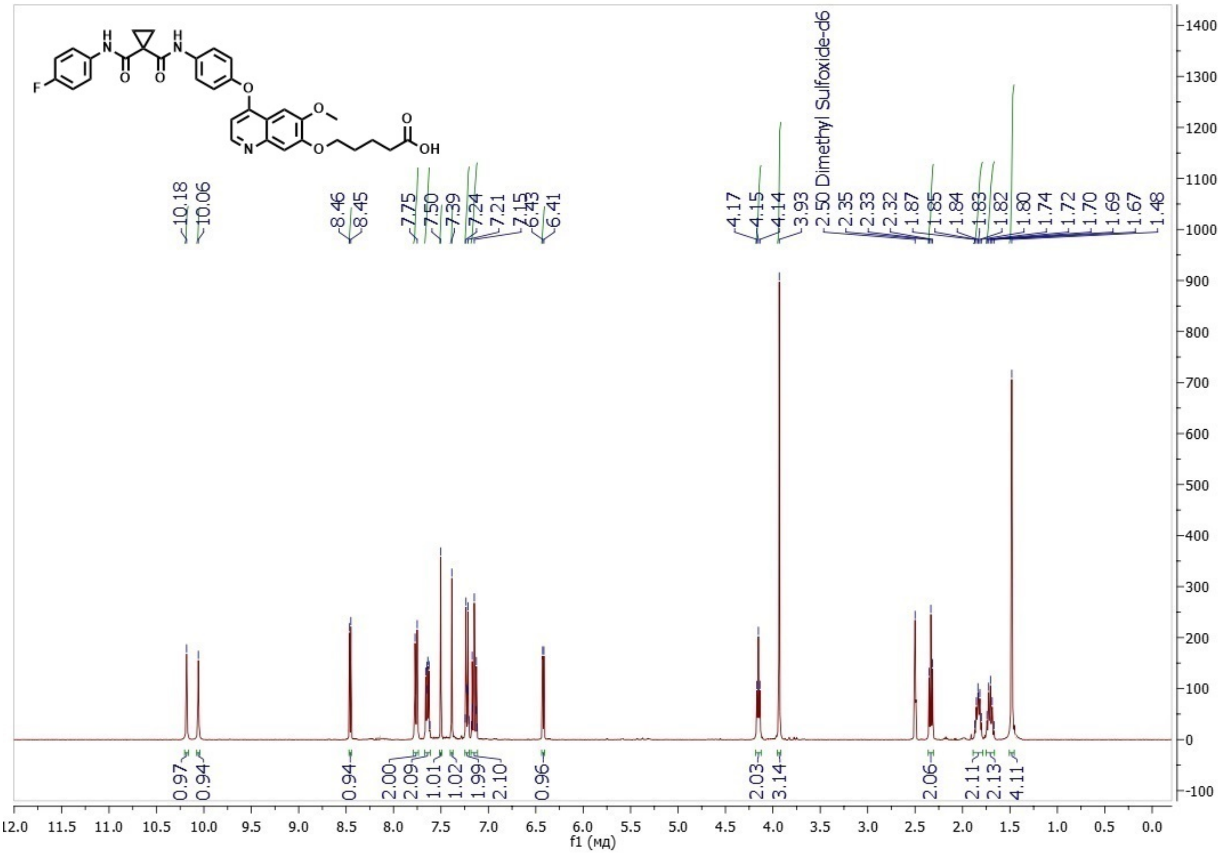
10.18
10.06
8.46
8.45
7.75
7.50
7.39
7.24
7.21
7.15
7.13
6.43
6.41
4.17
4.15
4.14
3.93
2.50 Dimethyl Sulfoxide-d6
2.35
2.33
2.32
1.87
1.85
1.84
1.83
1.82
1.80
1.74
1.72
1.70
1.69
1.67
1.48
0.97
0.94
0.94
2.00
2.09
1.01
1.02
1.99
2.10
0.96
2.03
3.14
2.06
2.11
2.13
4.11
f1 (мд)

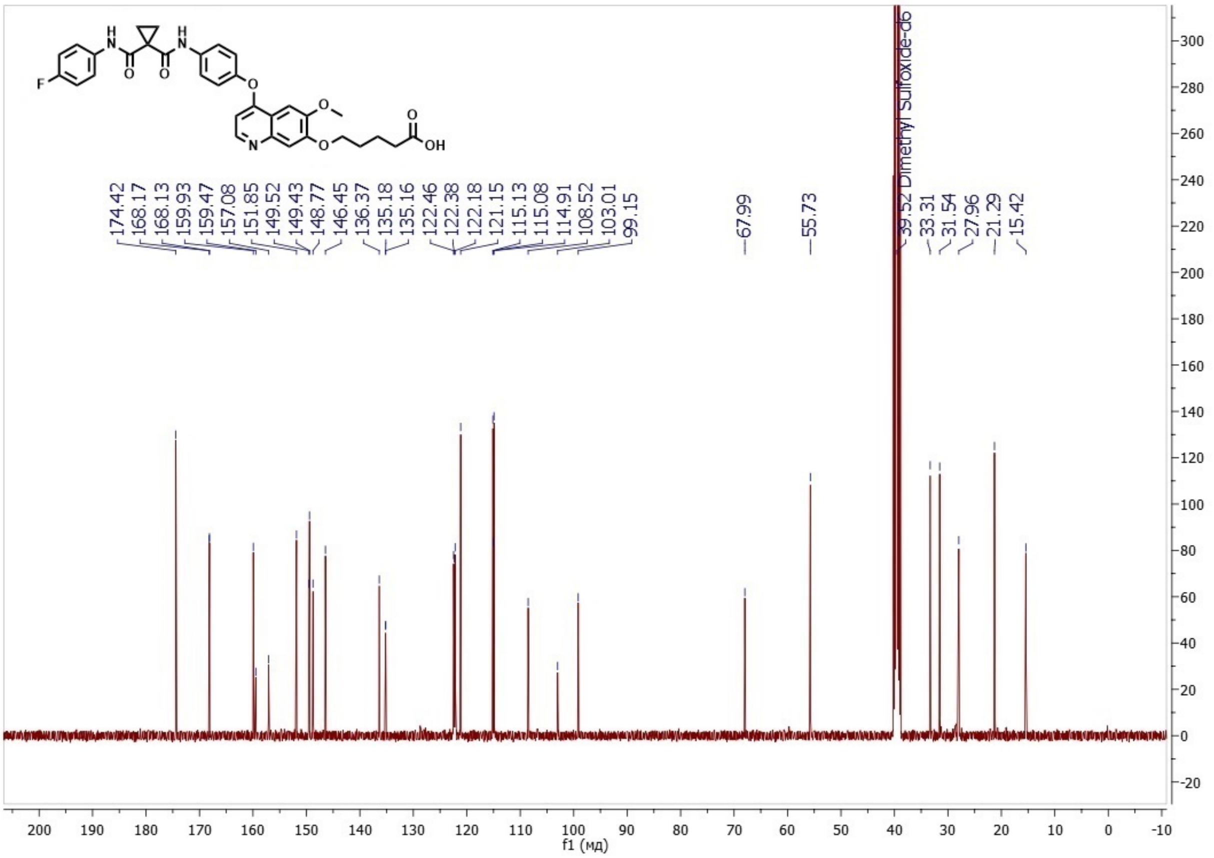
174.42
168.17
168.13
159.93
159.47
157.08
151.85
149.52
149.43
148.77
146.45
136.37
135.18
135.16
122.46
122.38
122.18
121.15
115.13
115.08
114.91
108.52
103.01
99.15
67.99
55.73
39.52 Dimethyl Sulfoxide-d6
33.31
31.54
27.96
21.29
15.42
f1 (мд)

Compound **5b**

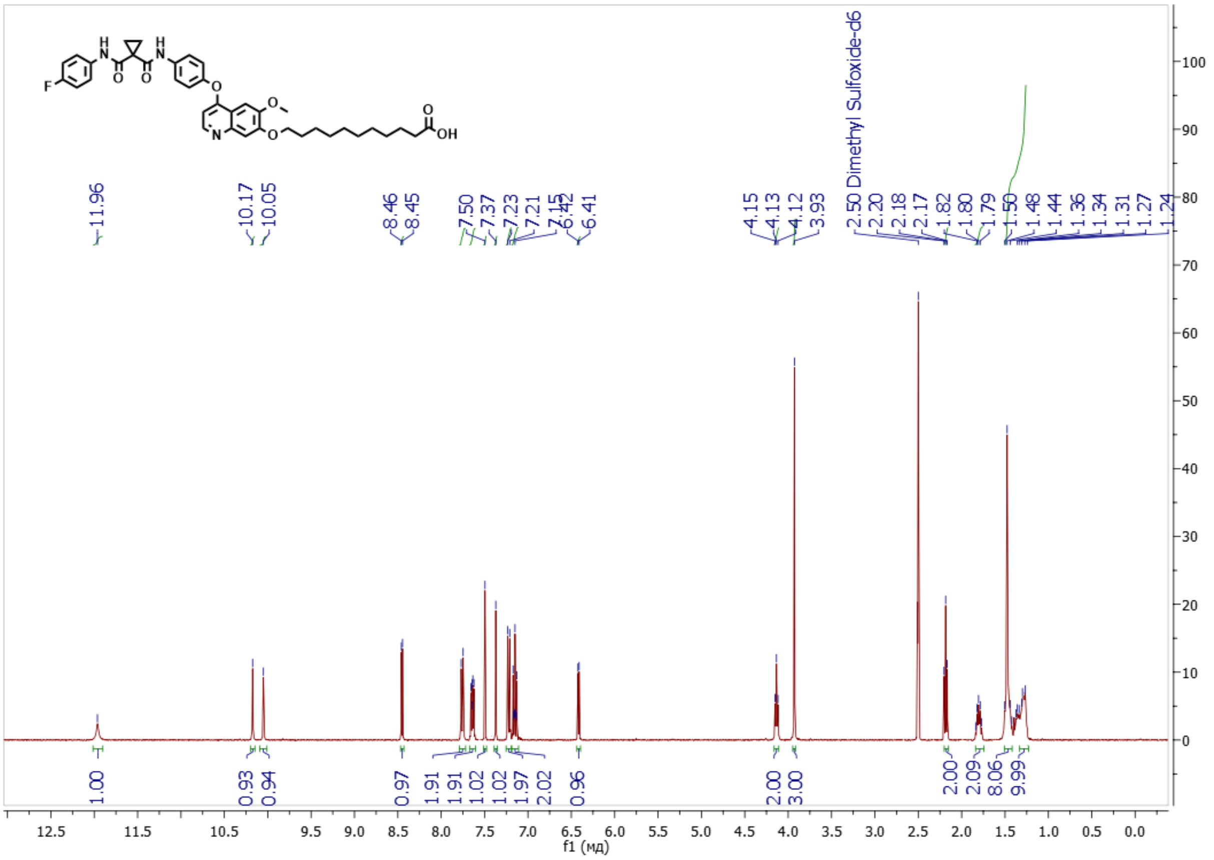

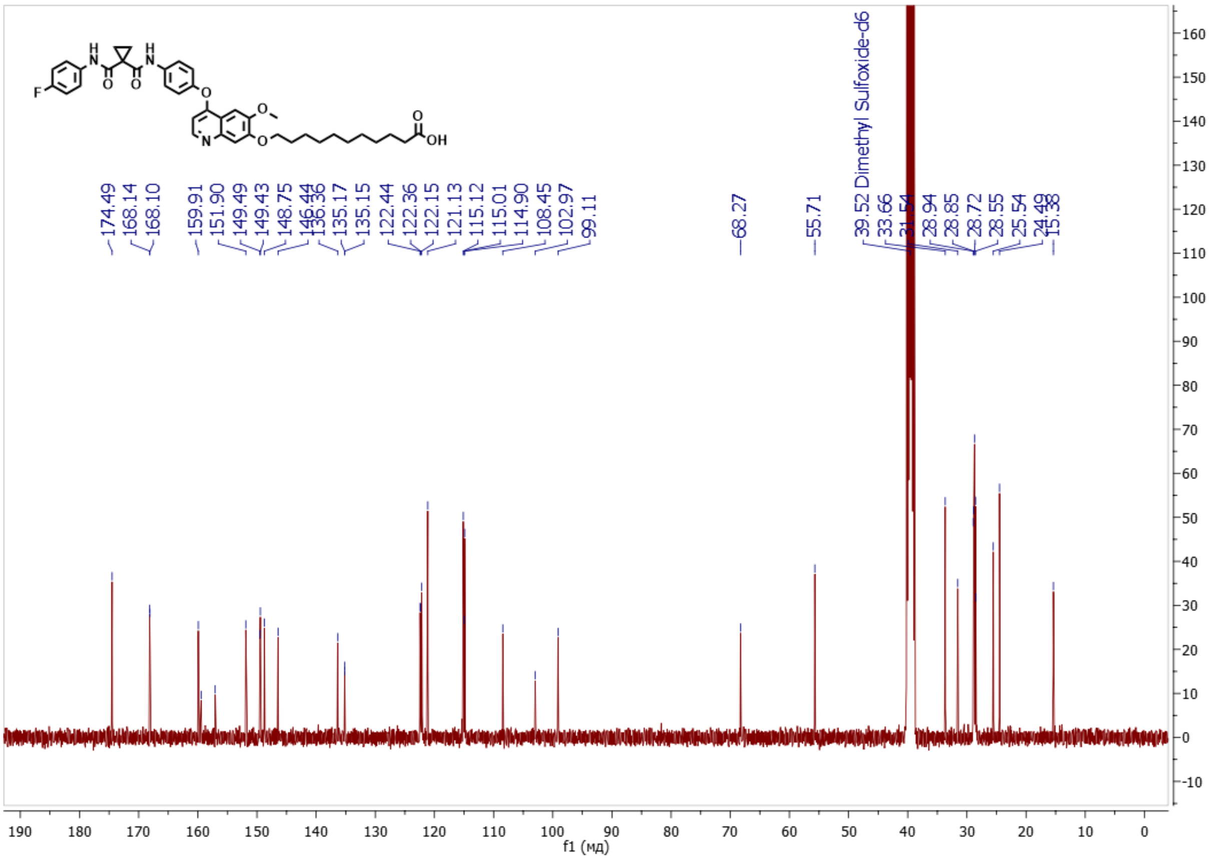

Compound **6a**

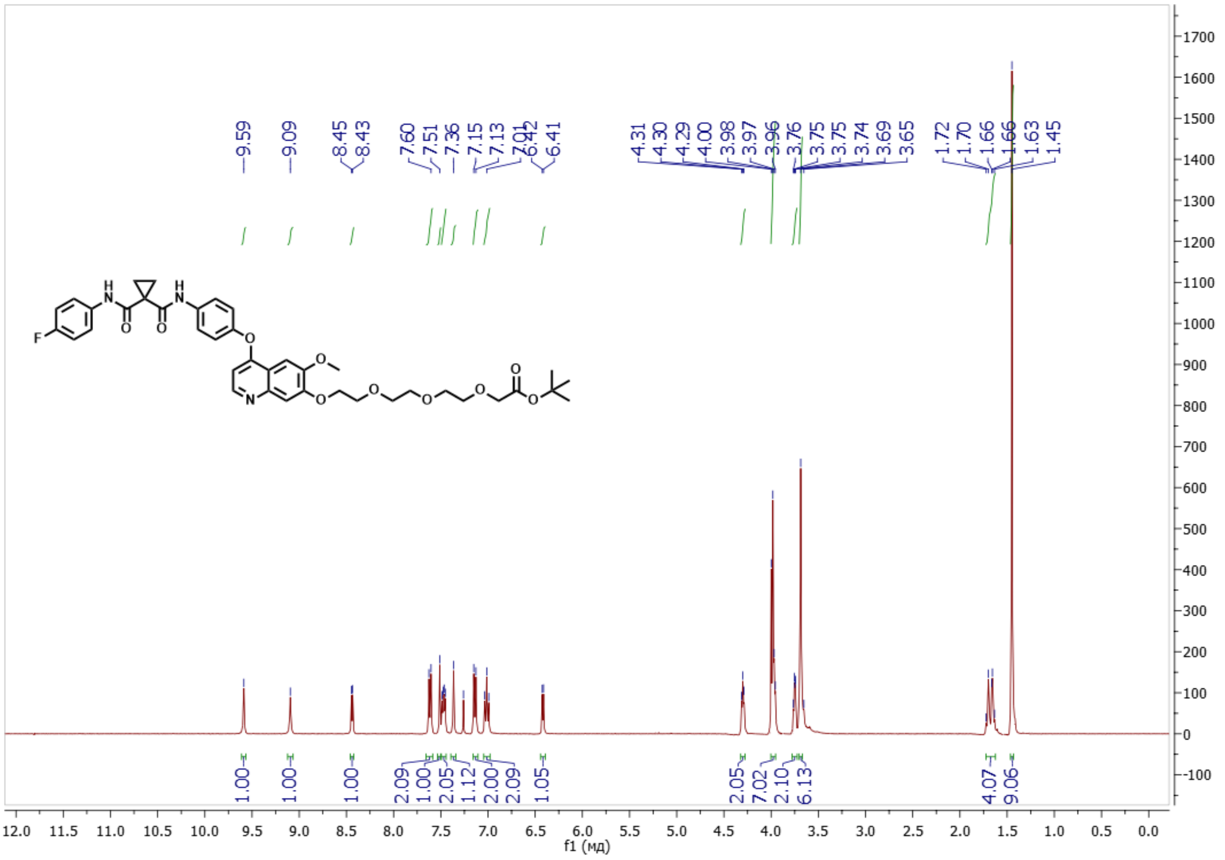

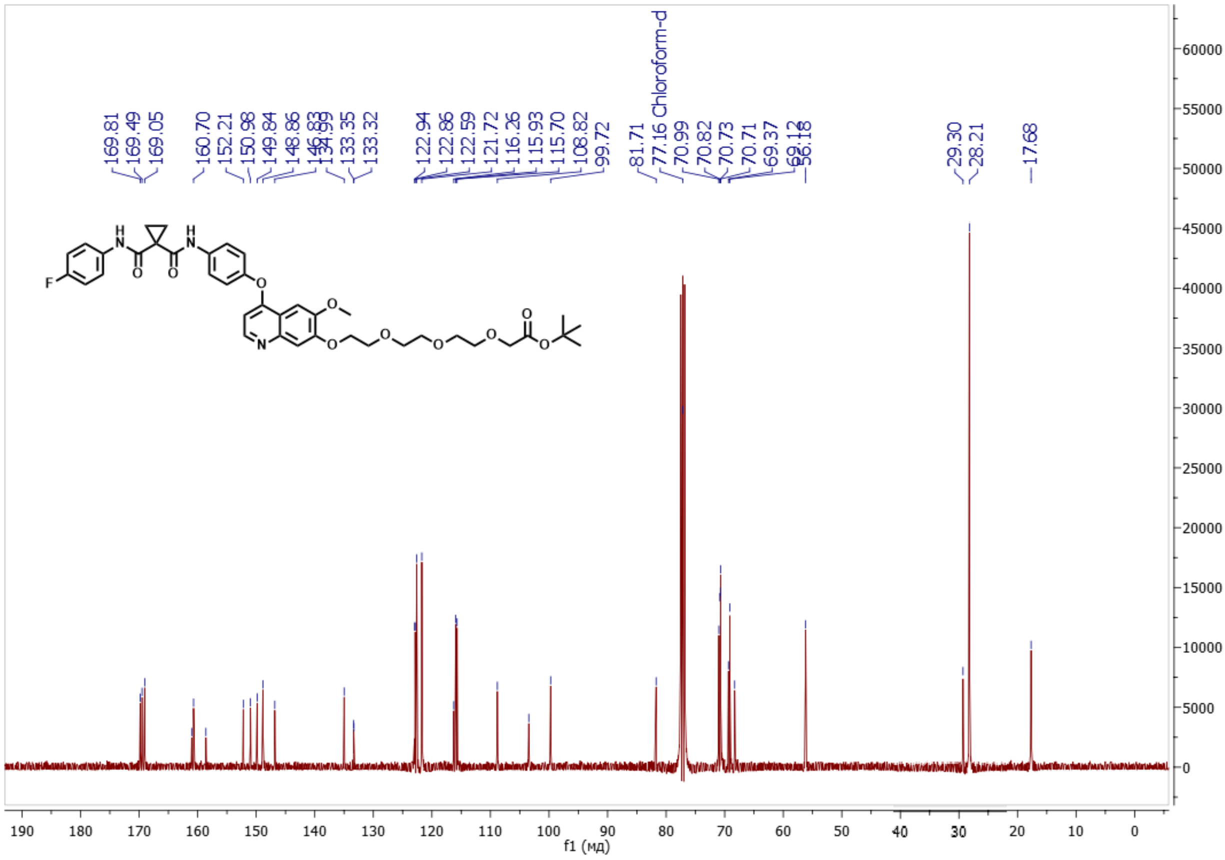

Compound **6b**

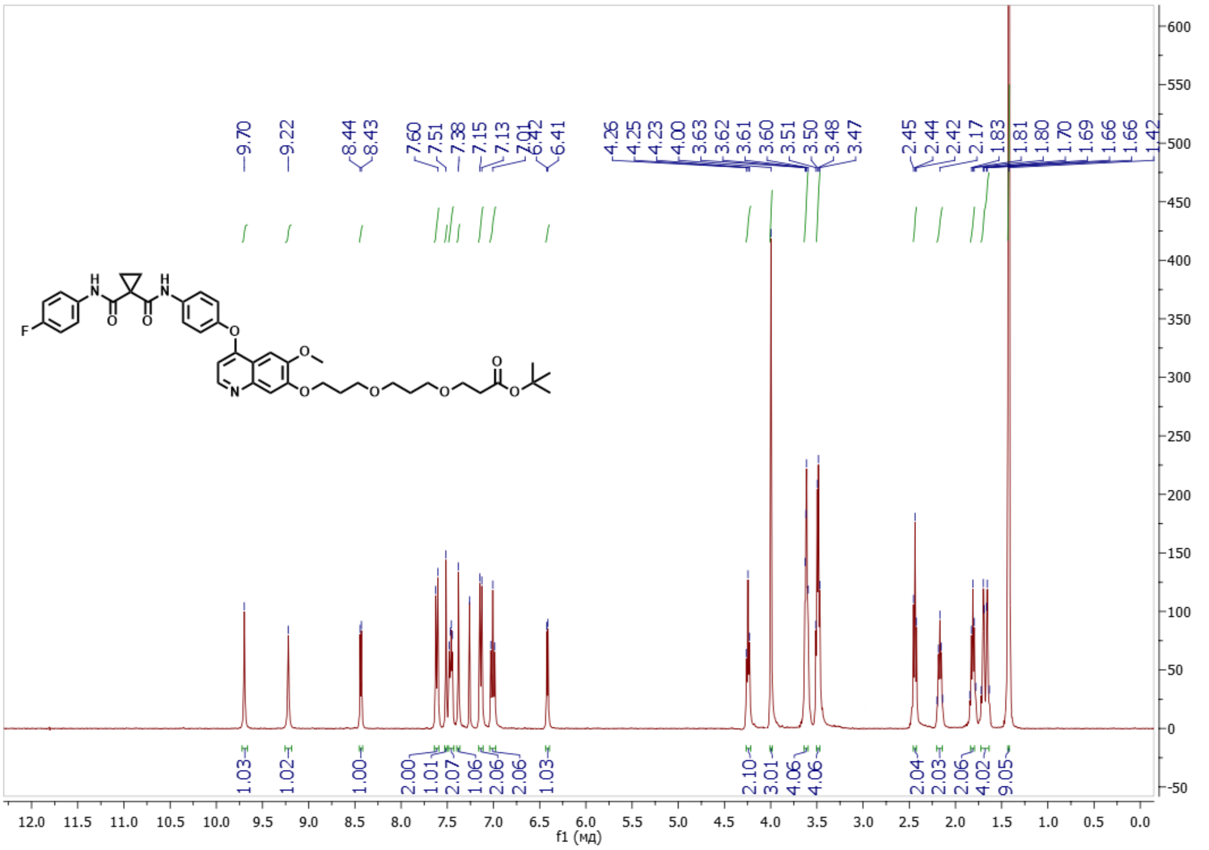

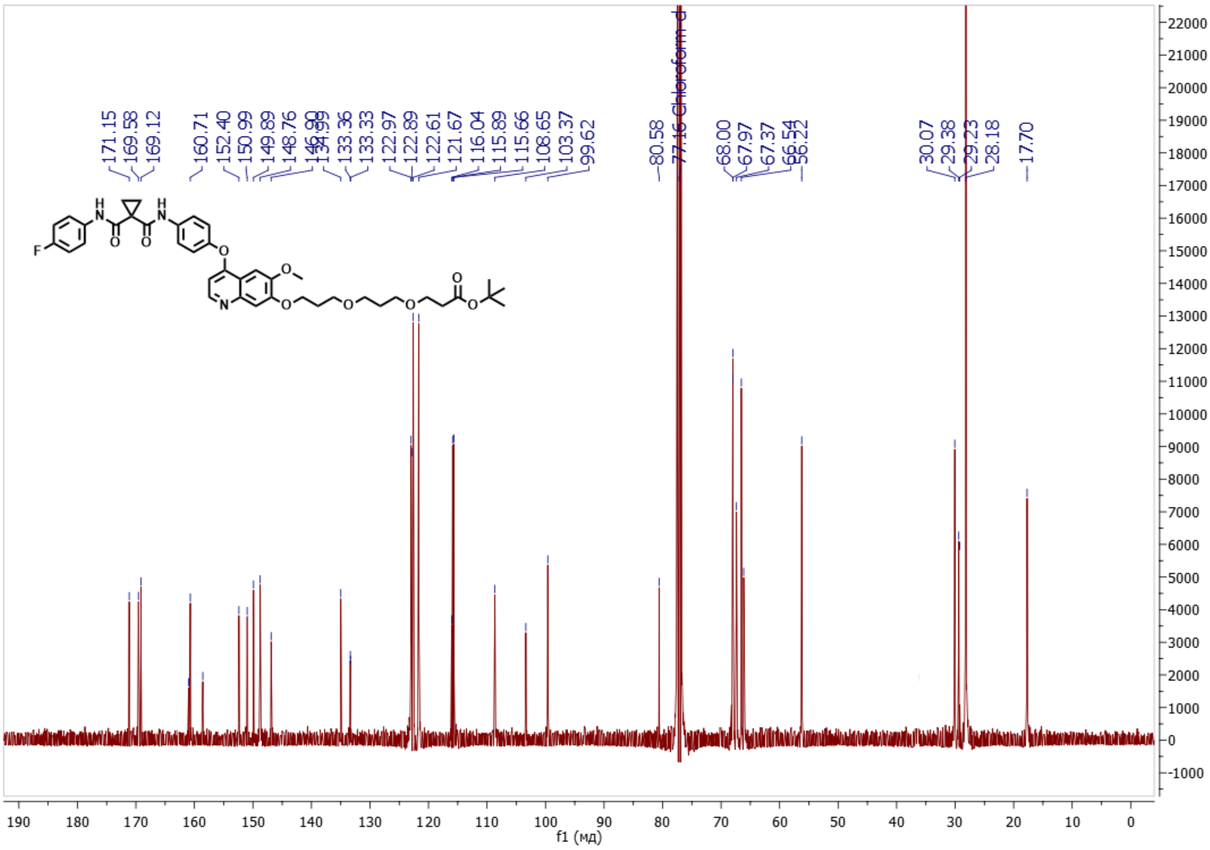

Compound **7a**

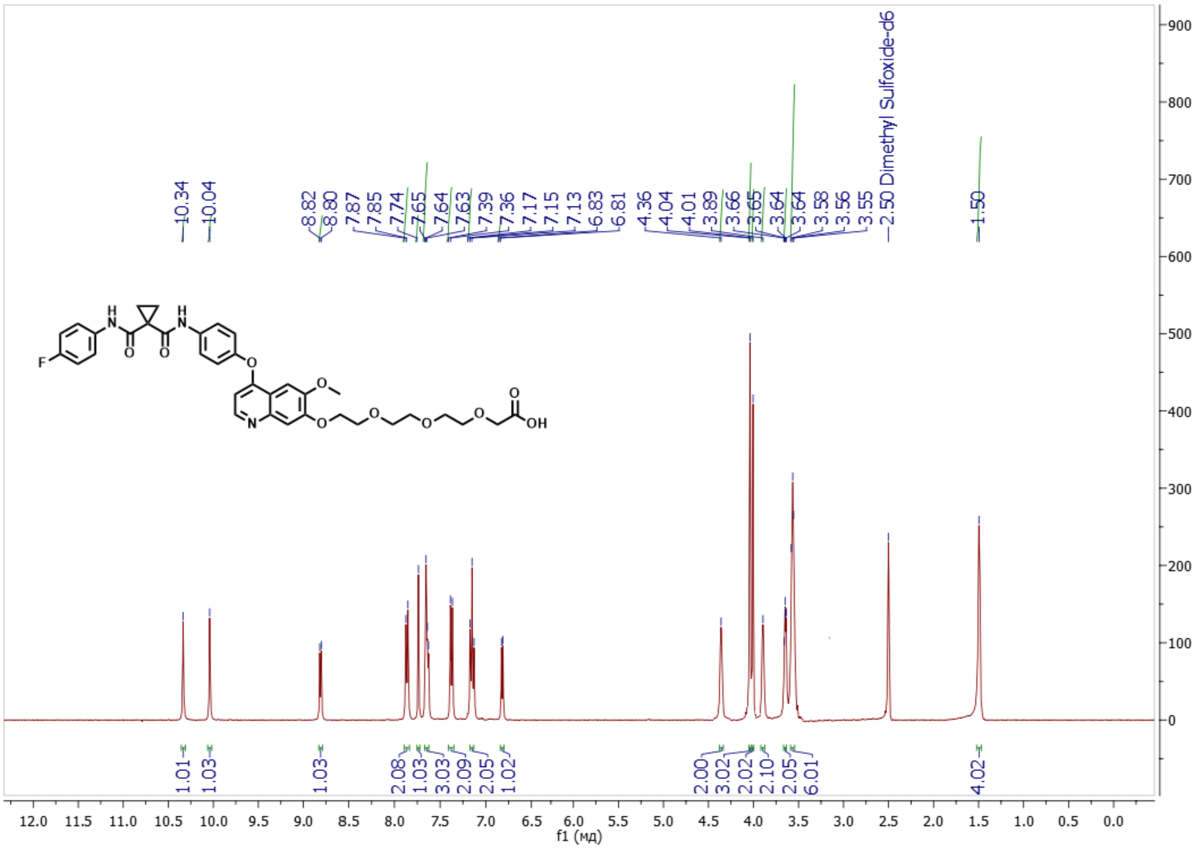

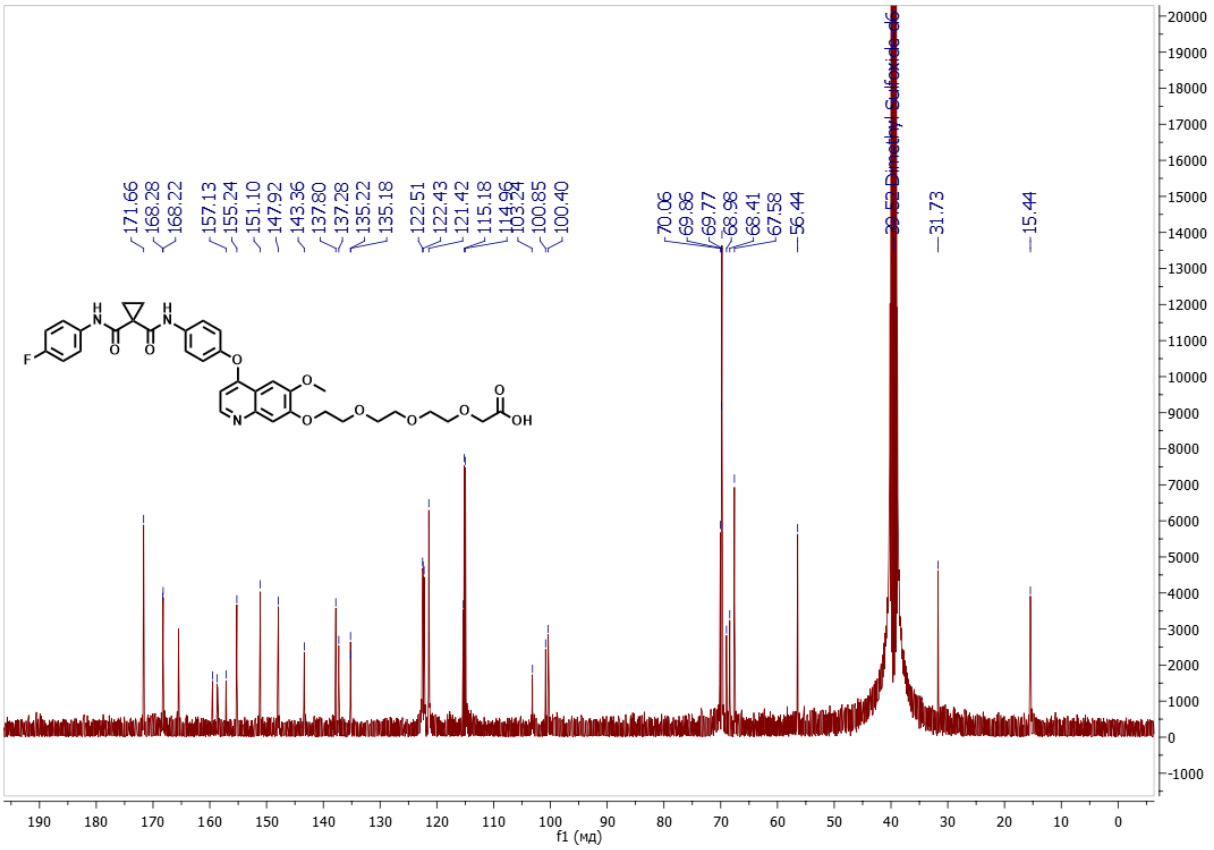

Compound **7b**

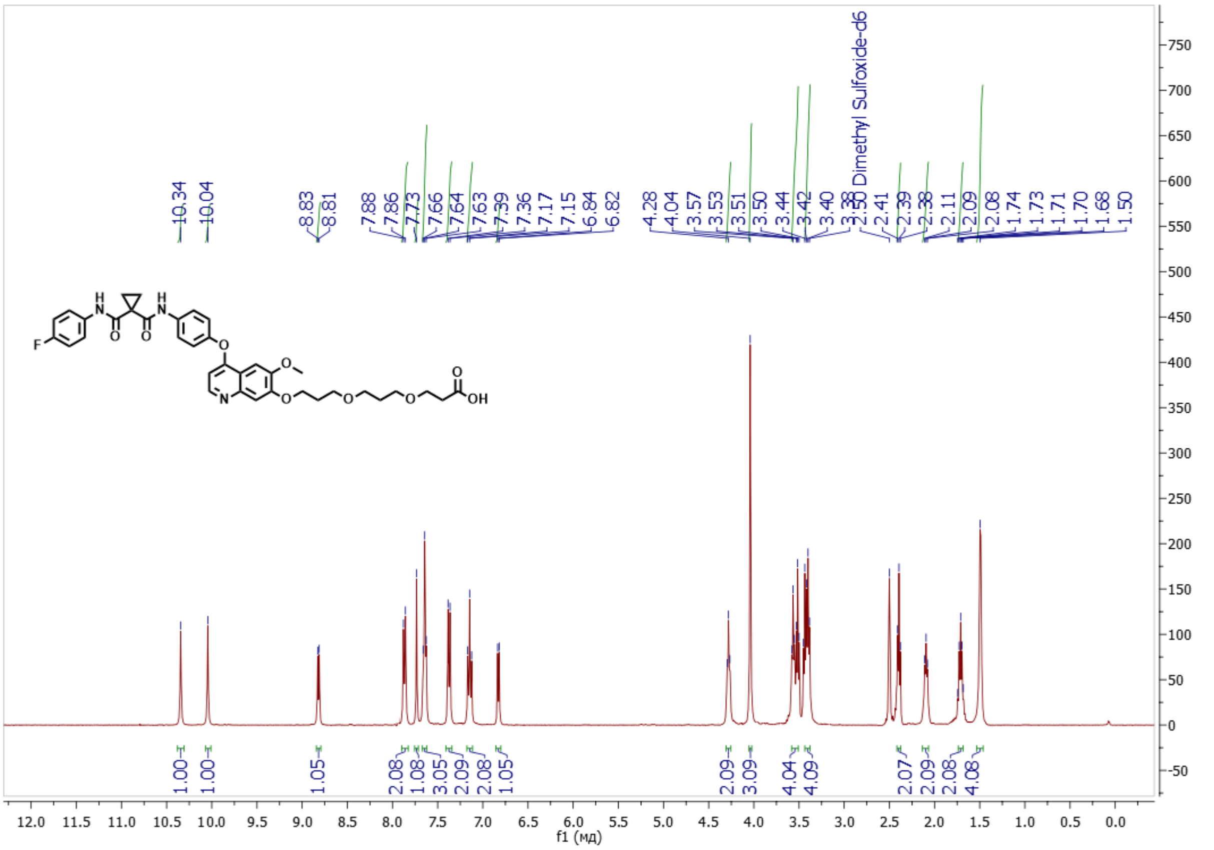

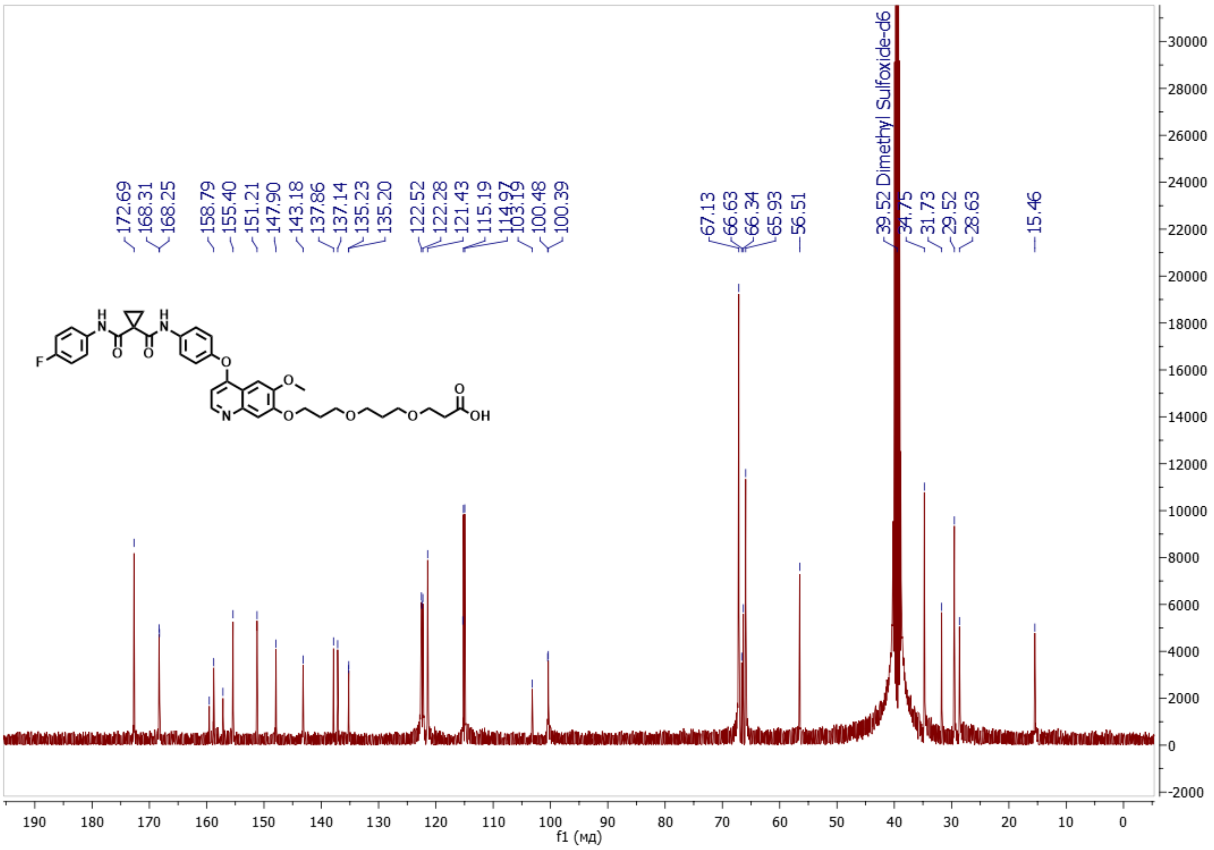

Compound **8a**

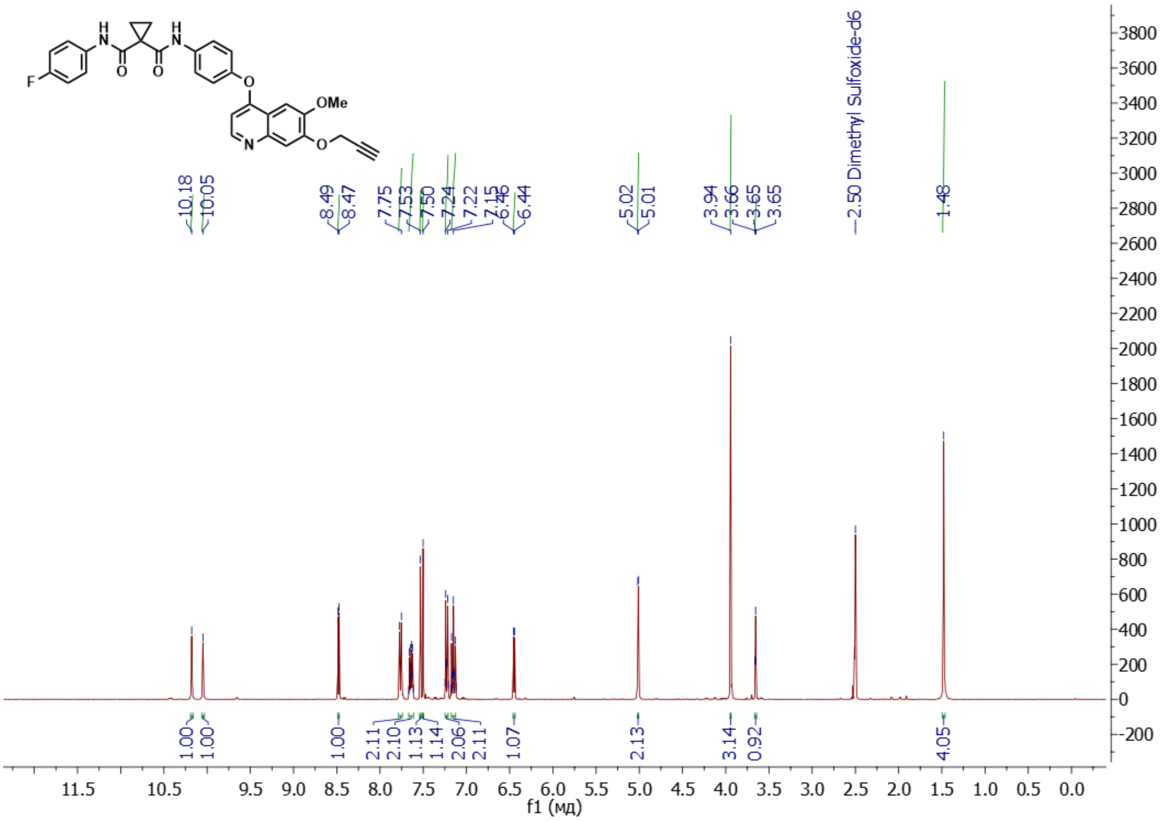

F
H N
O
OMe
N
10.18
10.05
8.49
8.47
7.75
7.53
7.50
7.24
7.22
7.15
6.44
5.02
5.01
3.94
3.66
3.65
3.65
2.50 Dimethyl Sulfoxide-d6
1.48
1.00
1.00
1.00
2.11
2.10
1.13
1.14
2.06
2.11
1.07
2.13
3.14
0.92
4.05
f1 (мд)


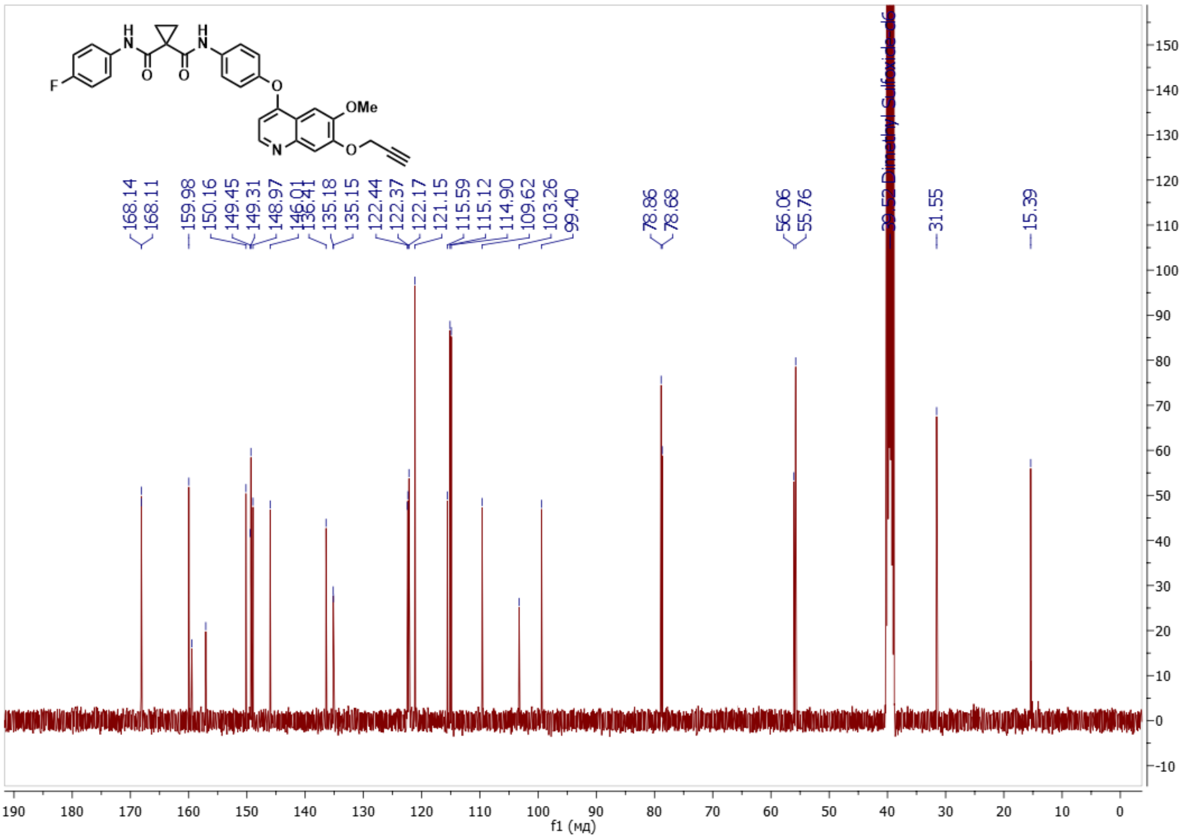

F
H N
O
OMe
N
168.14
168.11
159.98
150.16
149.45
149.31
148.97
135.18
135.15
122.44
122.37
122.17
121.15
115.59
115.12
114.90
109.62
103.26
99.40
78.86
78.68
56.06
55.76
Dimethyl Sulfoxide-d6
31.55
15.39
f1 (мд)

Compound **8b**

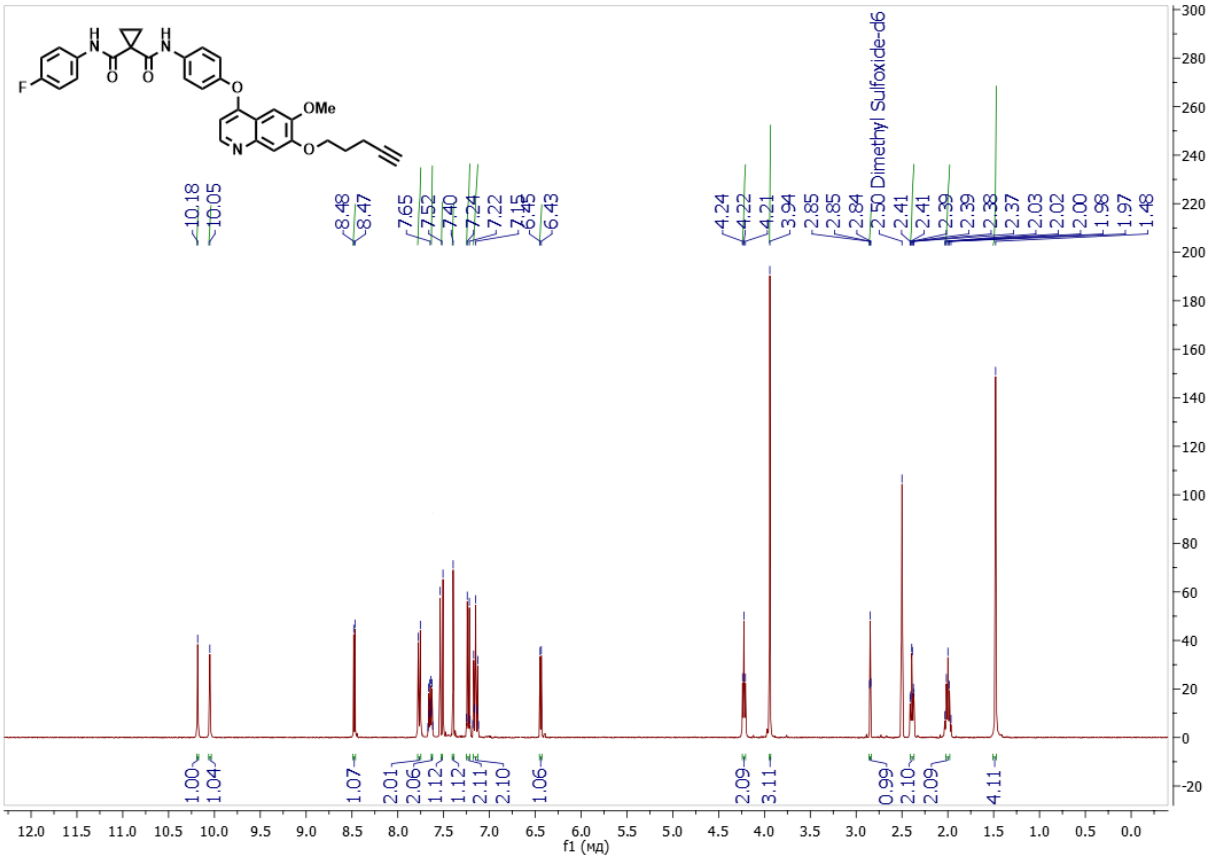

F
H N
O
OMe
N
10.18
10.05
8.48
8.47
7.65
7.52
7.40
7.24
7.22
6.43
4.24
4.22
4.21
3.94
2.85
2.85
2.84
2.50 Dimethyl Sulfoxide-d6
2.41
2.41
2.39
2.39
2.38
2.37
2.03
2.02
2.00
1.98
1.97
1.48
1.00
1.04
1.07
2.01
2.06
1.12
1.12
2.11
2.10
1.06
2.09
3.11
0.99
2.10
2.09
4.11
f1 (мд)


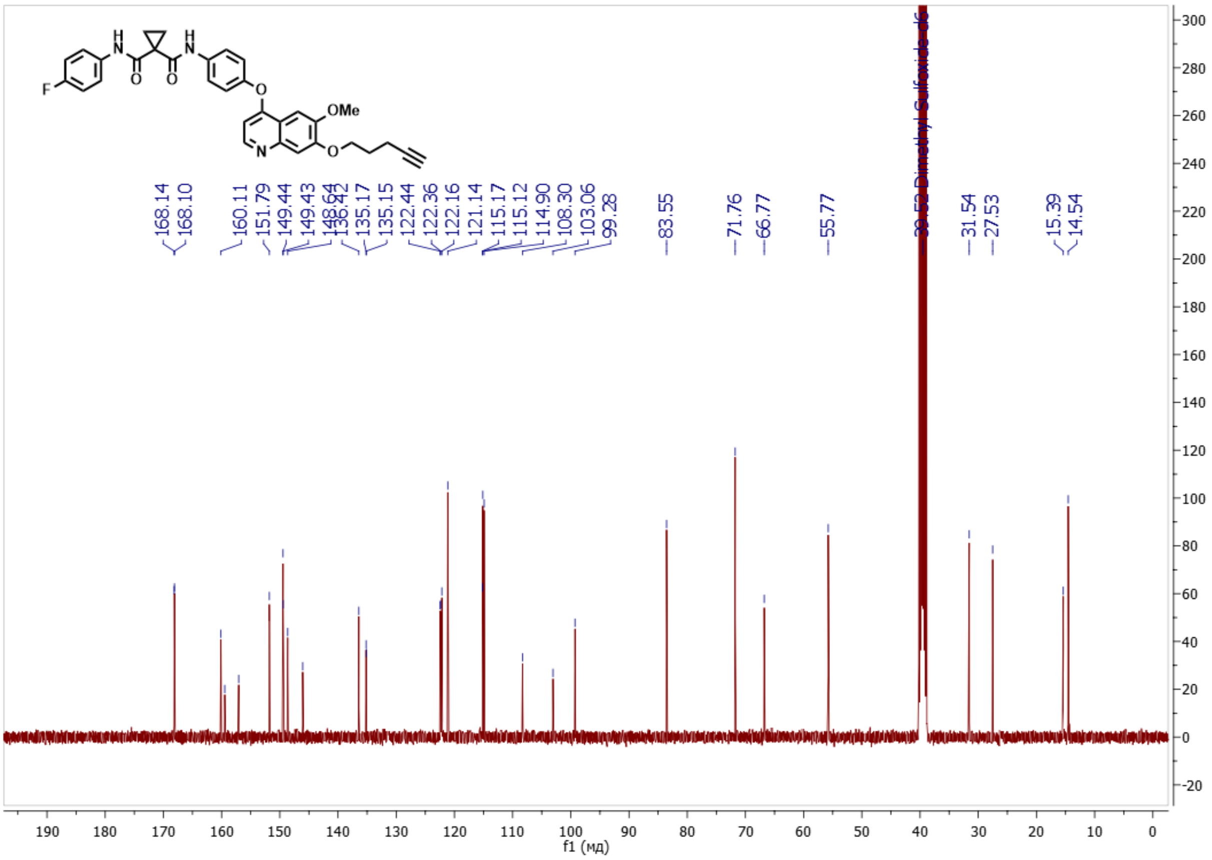

F
H N
O
OMe
N
168.14
168.10
160.11
151.79
149.44
149.43
135.17
135.15
122.44
122.36
122.16
121.14
115.17
115.12
114.90
108.30
103.06
99.28
83.55
71.76
66.77
55.77
31.54
27.53
15.39
14.54
f1 (мд)

Compound **8c**

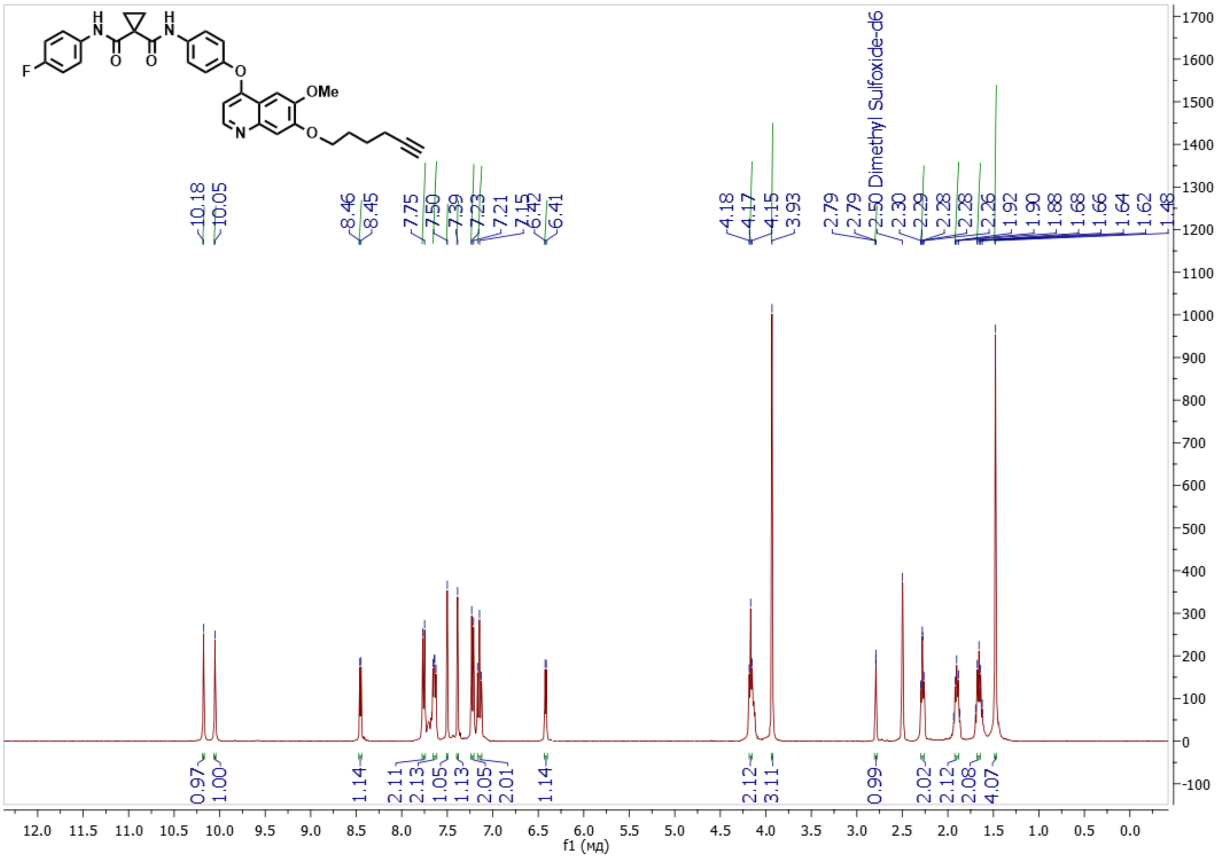

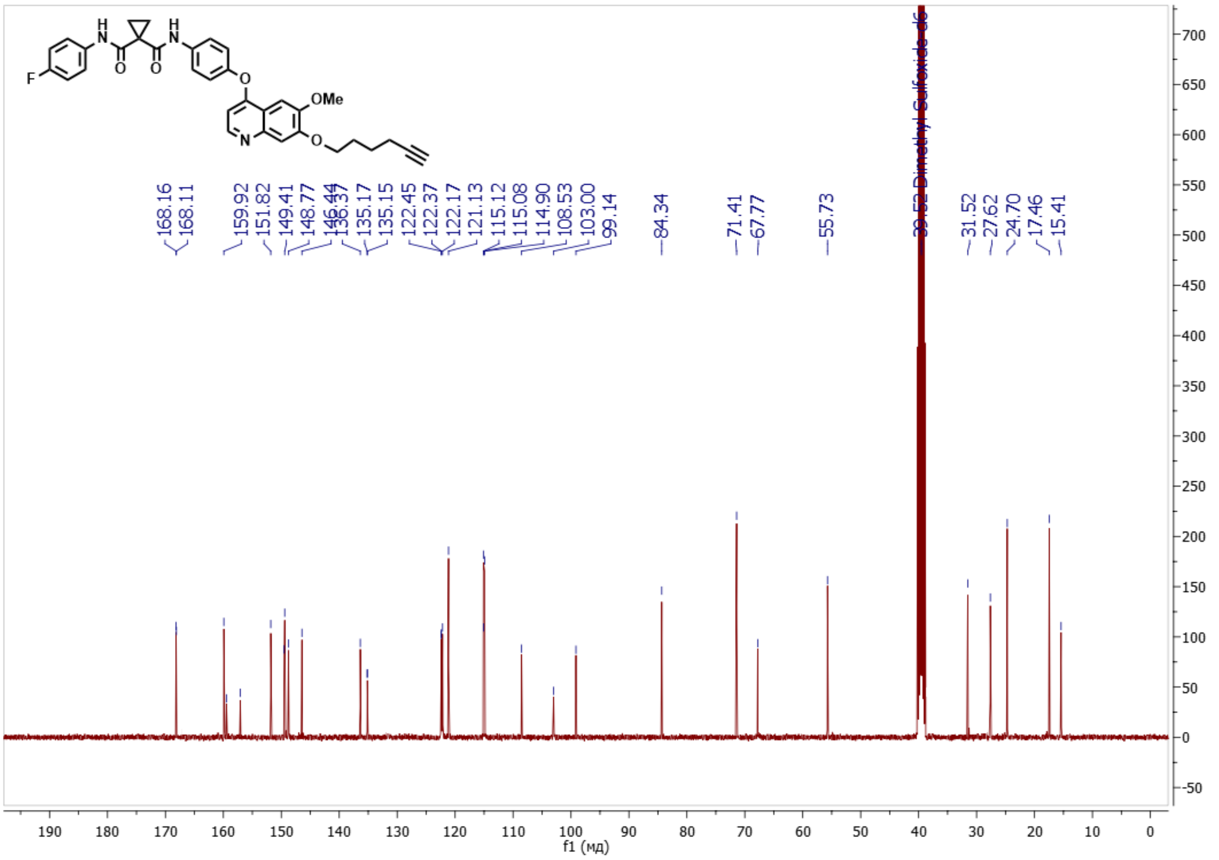

Compound **8d**

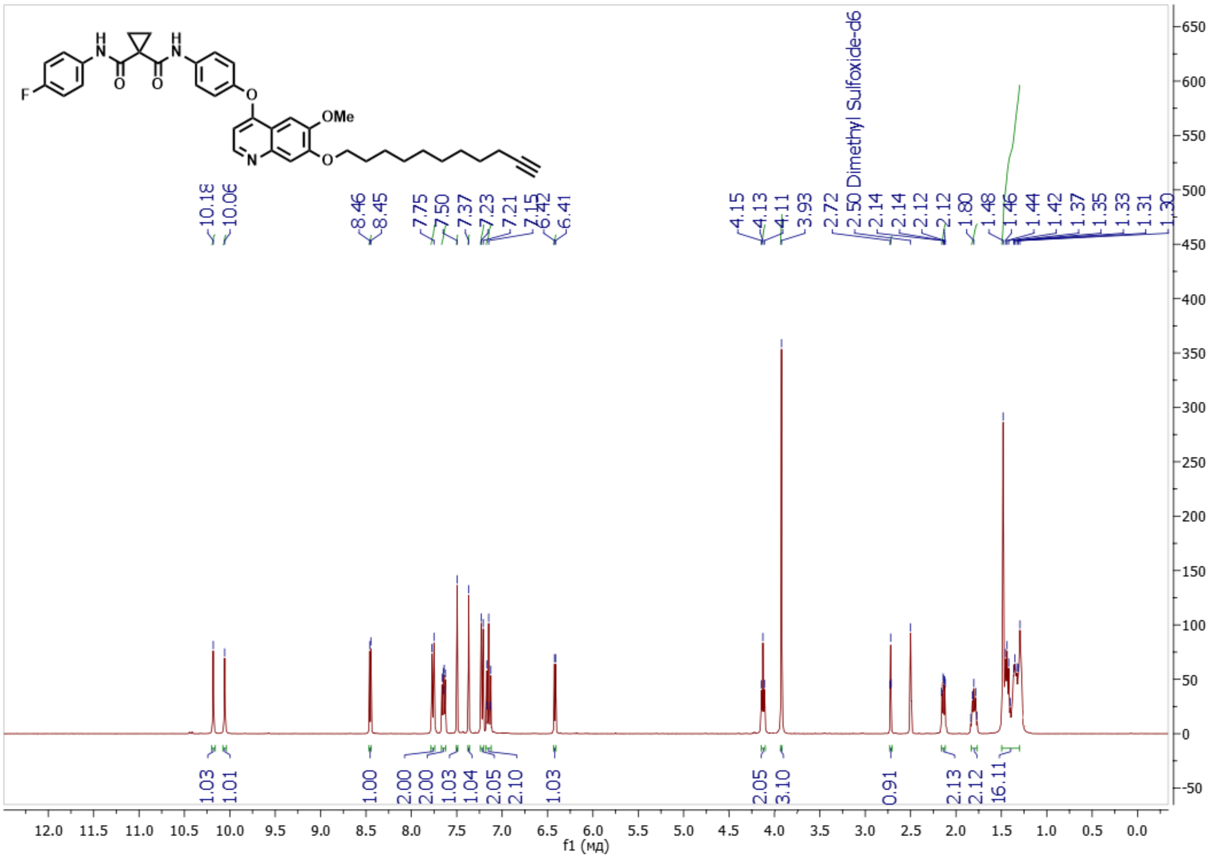

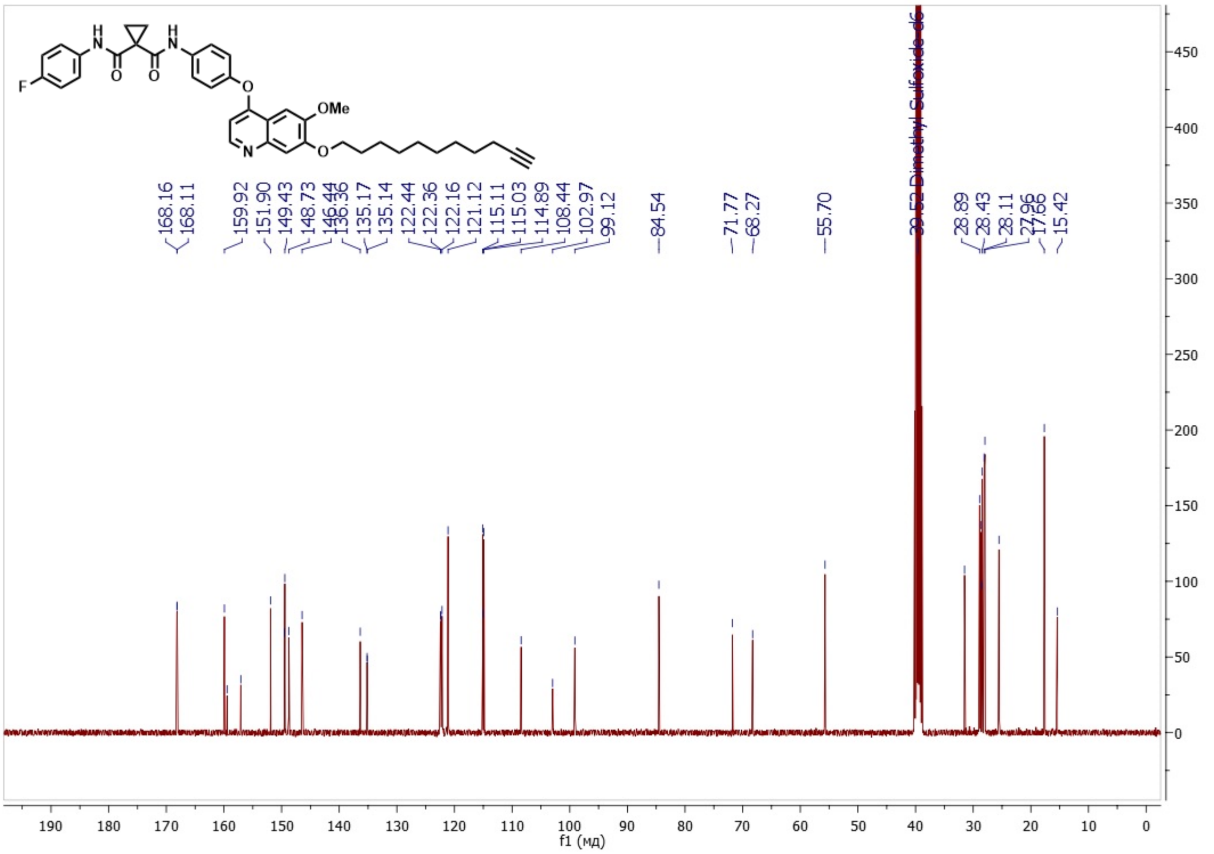

Compound **9a**

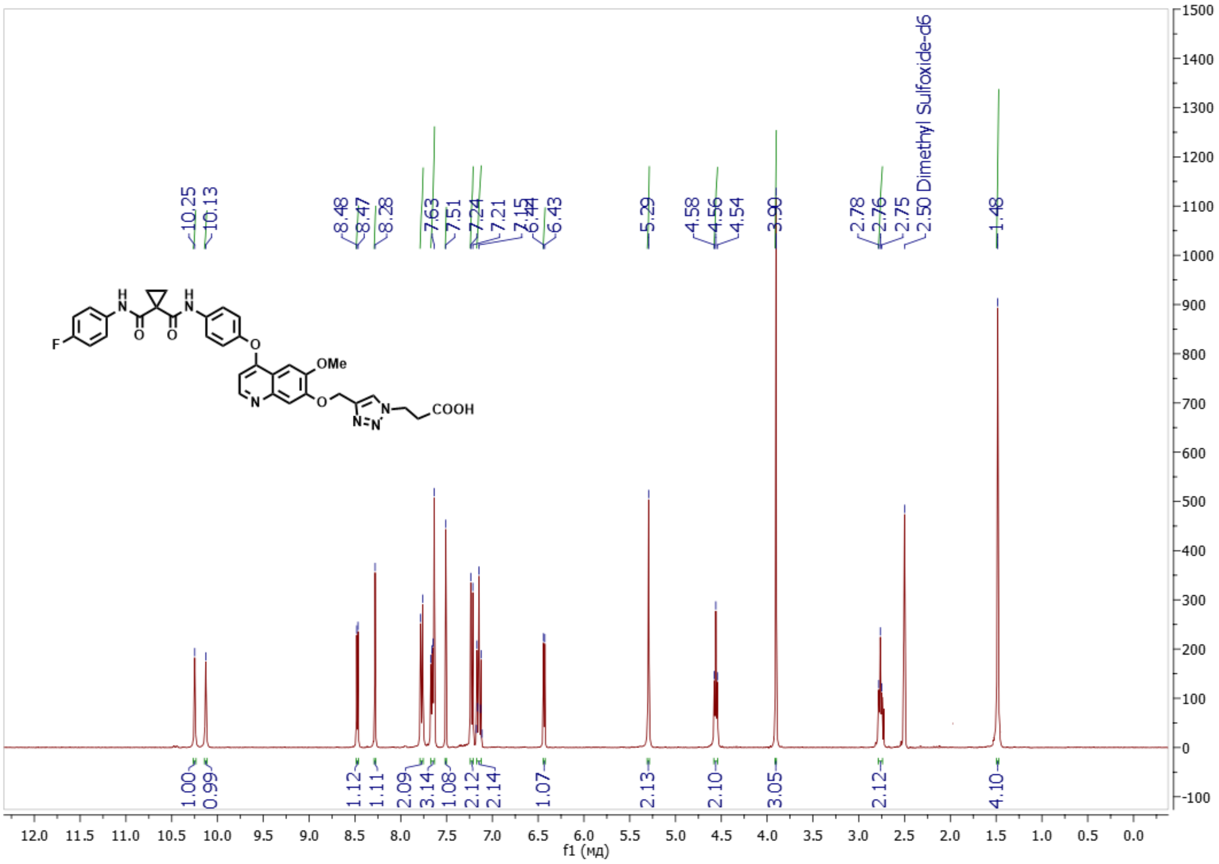

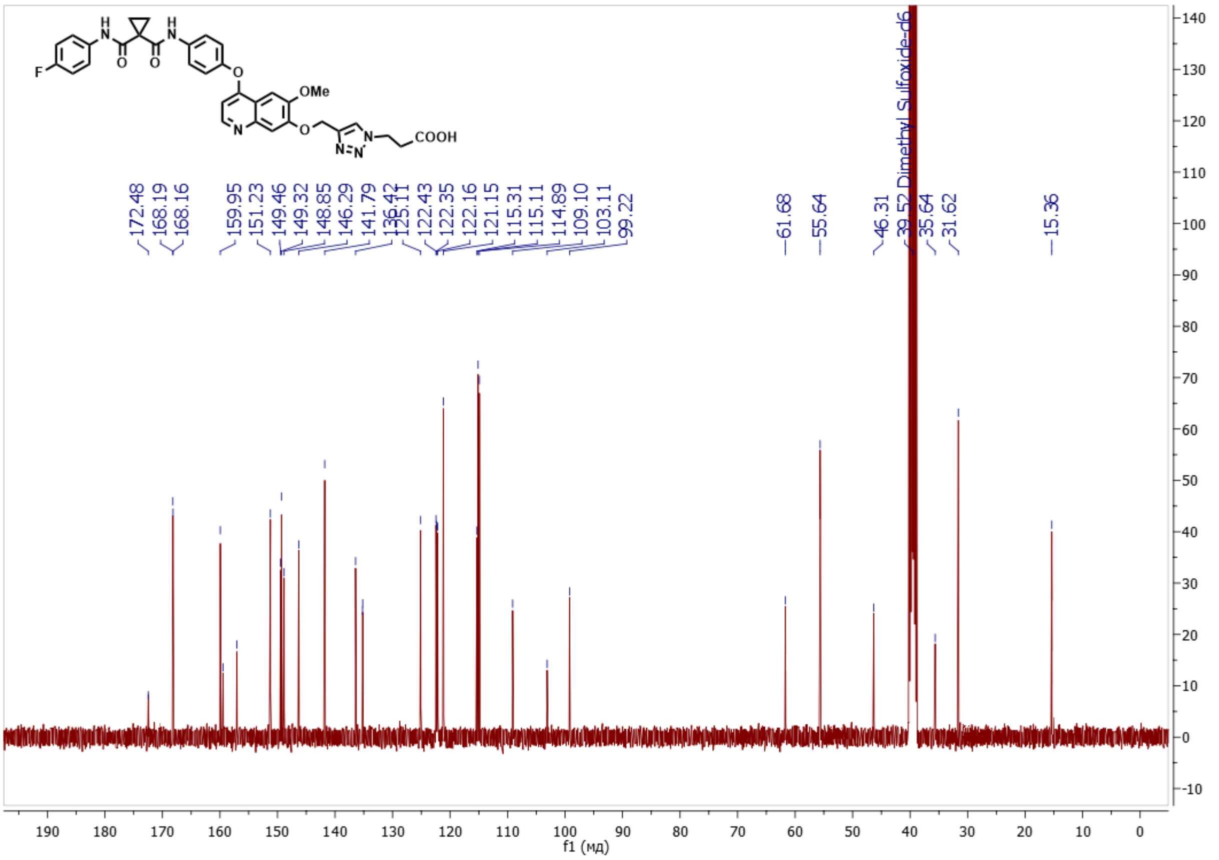

Compound **9b**

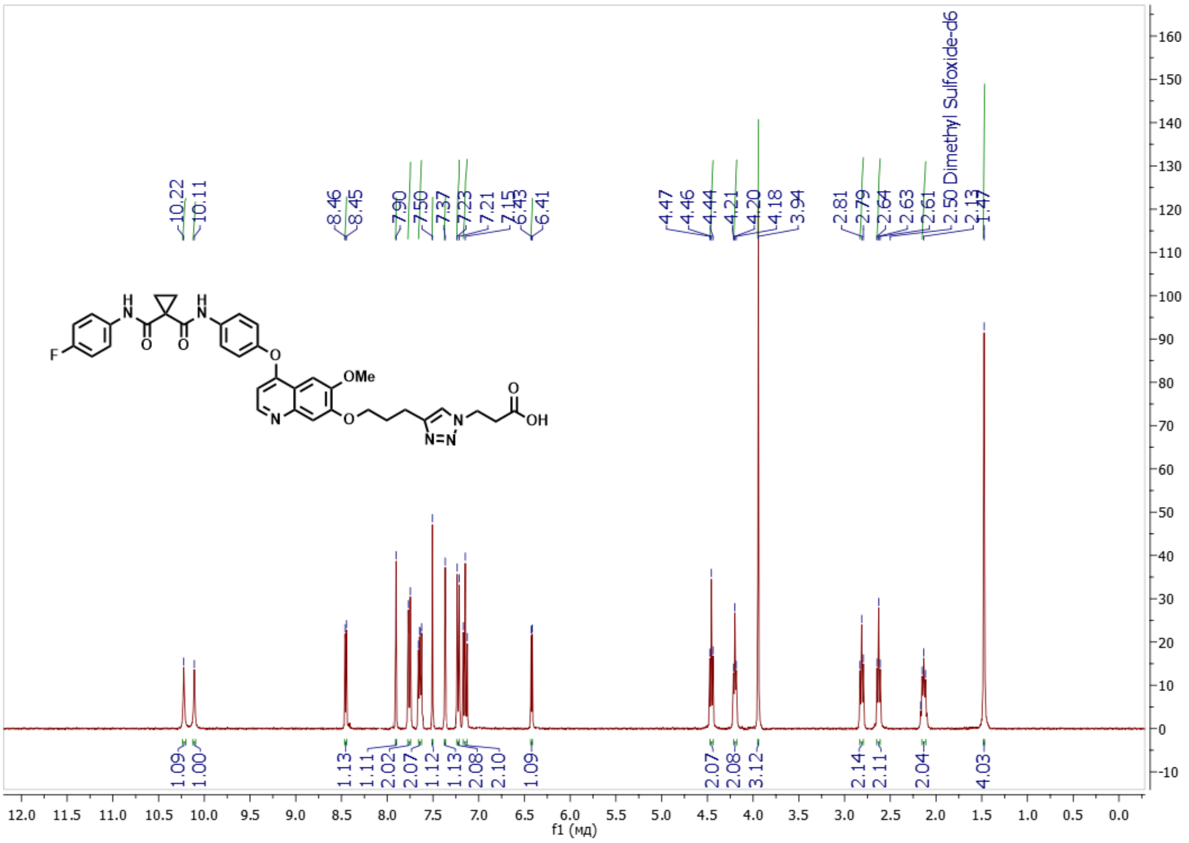

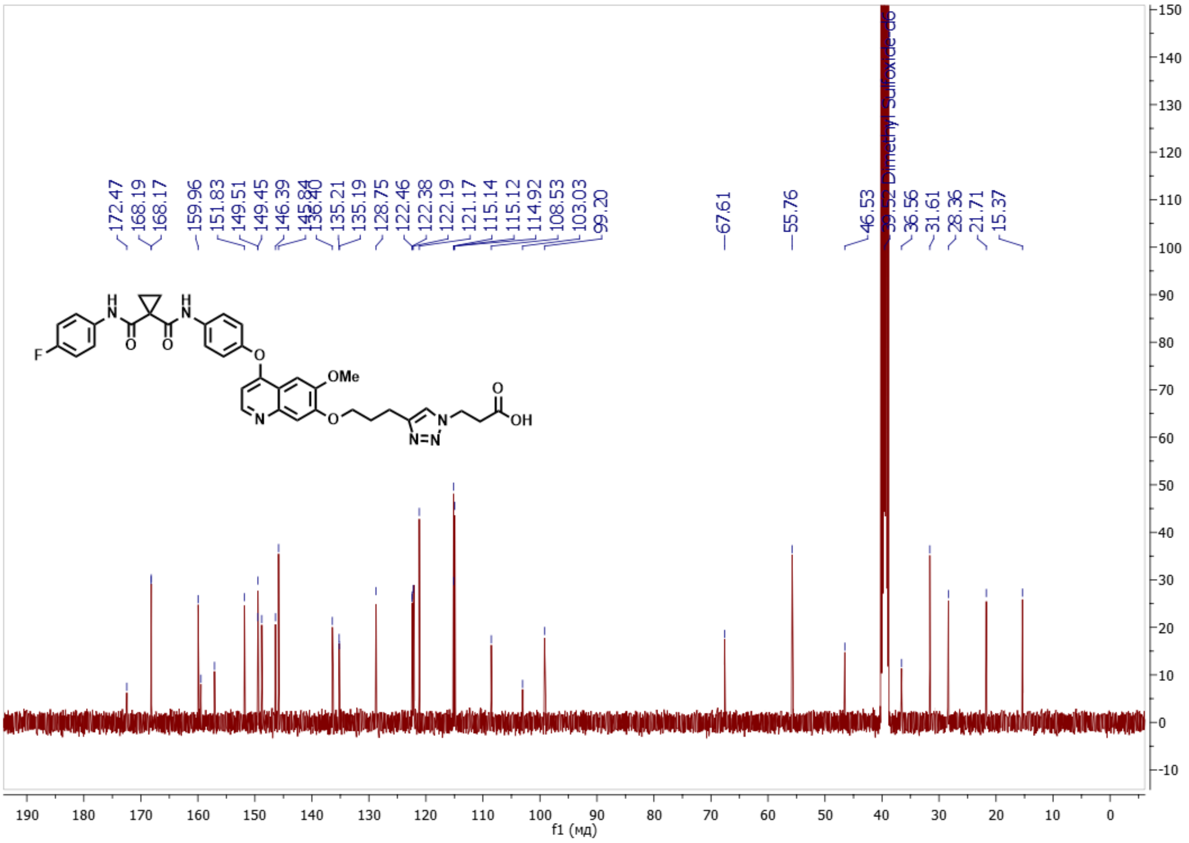

Compound **9c**

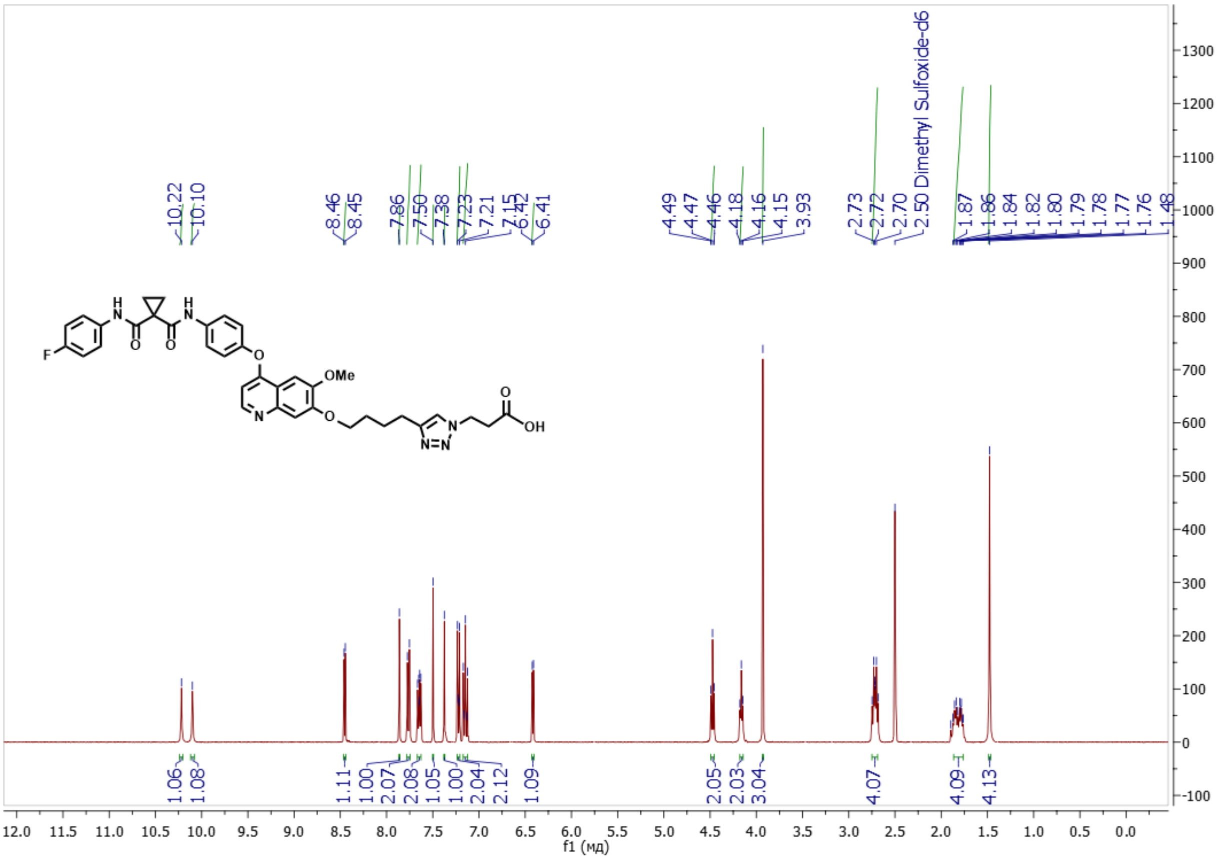

Dimethyl Sulfoxide-d6
10.22
10.10
8.46
8.45
7.86
7.50
7.38
7.23
7.21
7.15
6.42
6.41
4.49
4.47
4.46
4.18
4.16
4.15
3.93
2.73
2.72
2.70
2.50 Dimethyl Sulfoxide-d6
1.87
1.86
1.84
1.82
1.80
1.79
1.78
1.77
1.76
1.48
1.06
1.08
1.11
1.00
2.07
2.08
1.05
1.00
2.04
2.12
1.09
2.05
2.03
3.04
4.07
4.09
4.13
f1 (мд)


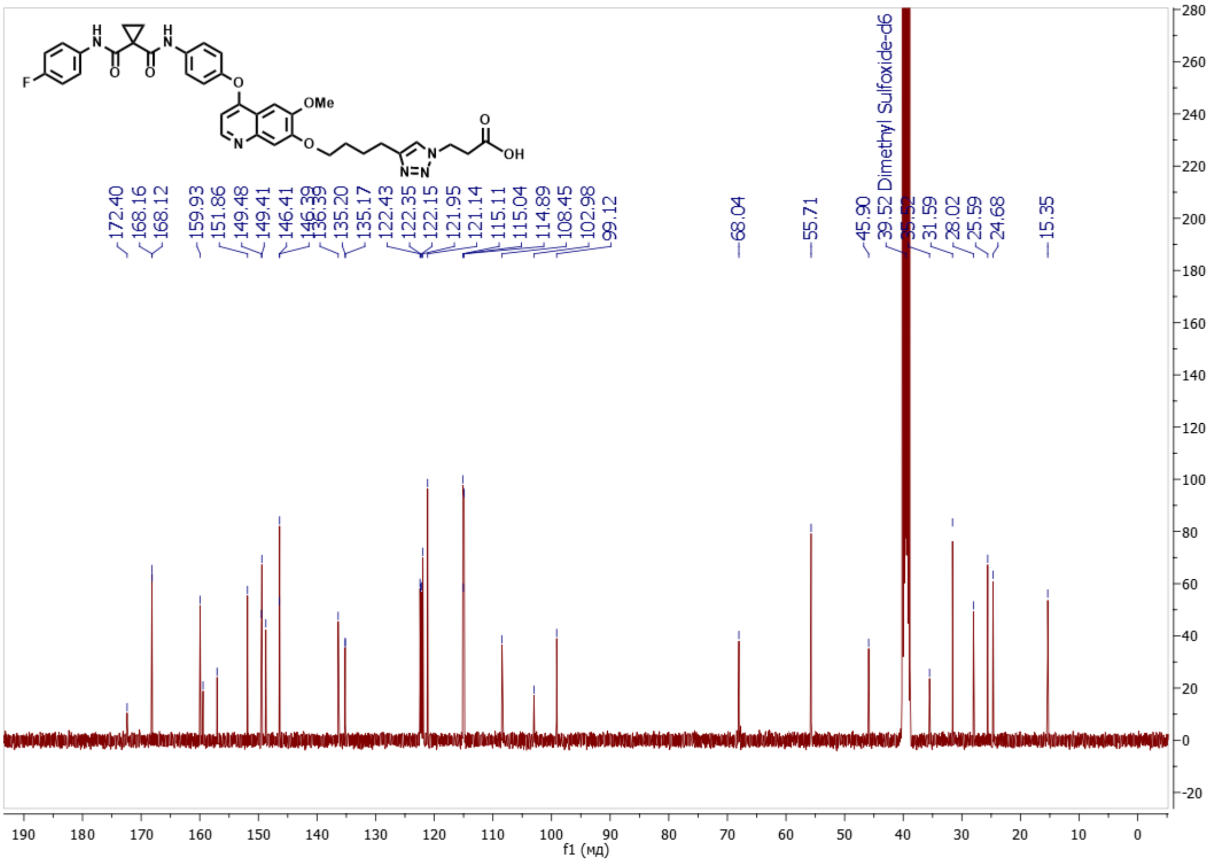

172.40
168.16
168.12
159.93
151.86
149.48
149.41
146.41
146.39
136.39
135.20
135.17
122.43
122.35
122.15
121.95
121.14
115.11
115.04
114.89
108.45
102.98
99.12
68.04
55.71
45.90
39.52 Dimethyl Sulfoxide-d6
35.92
31.59
28.02
25.59
24.68
15.35
f1 (мд)

Compound **9d**

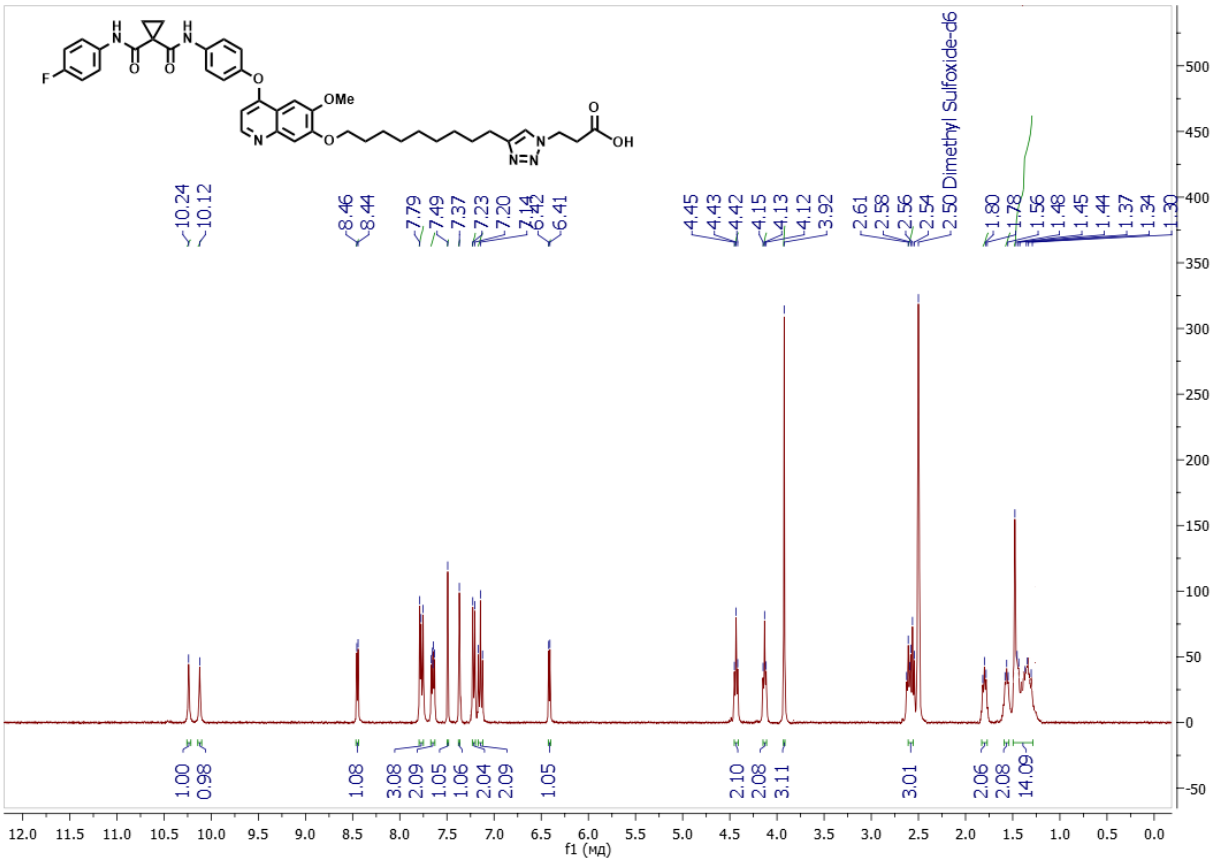


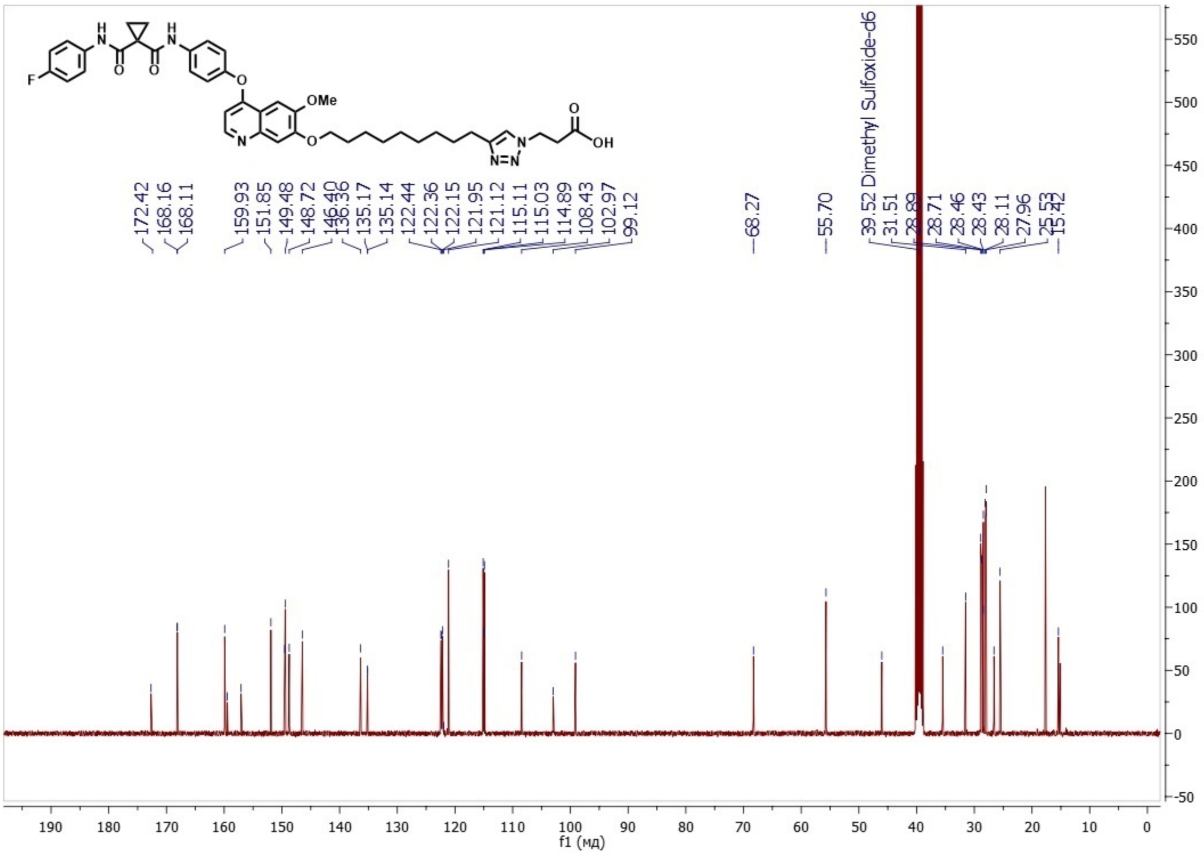

Compound **11a**

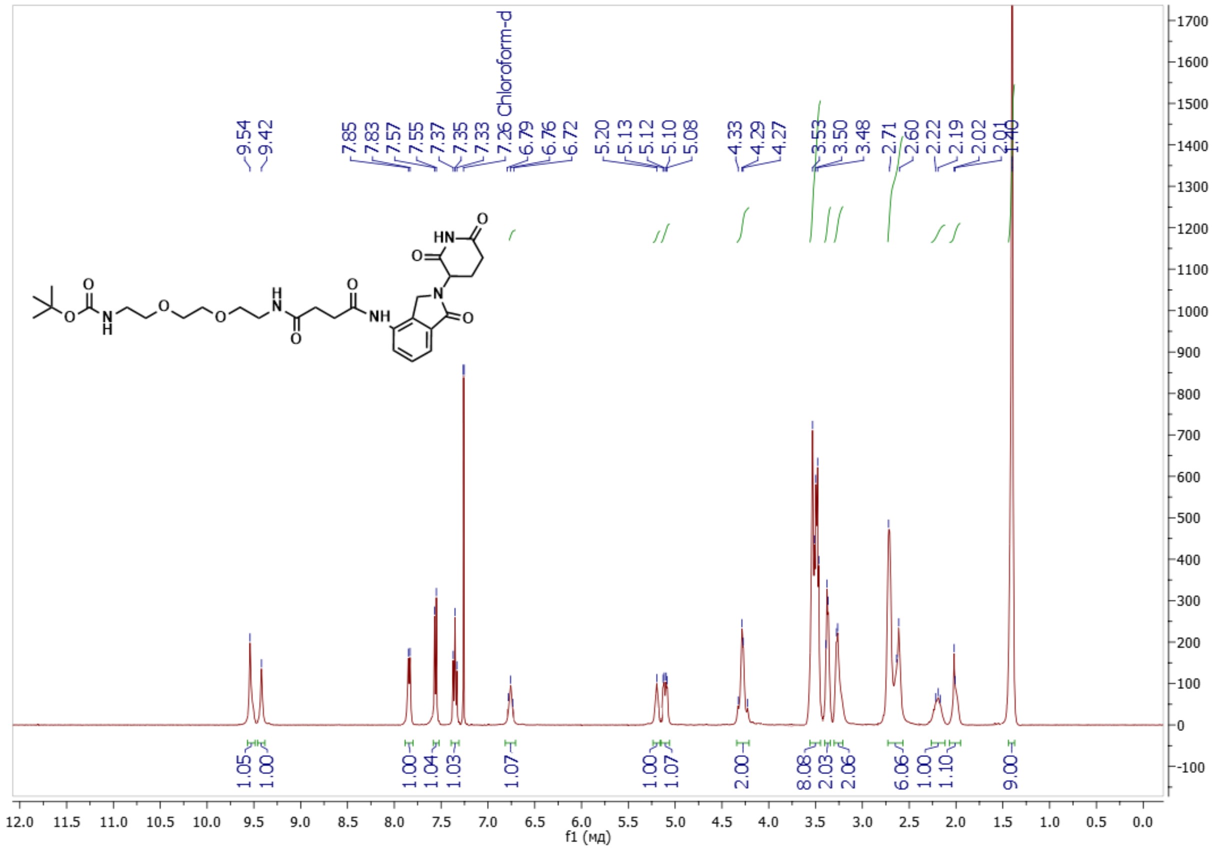

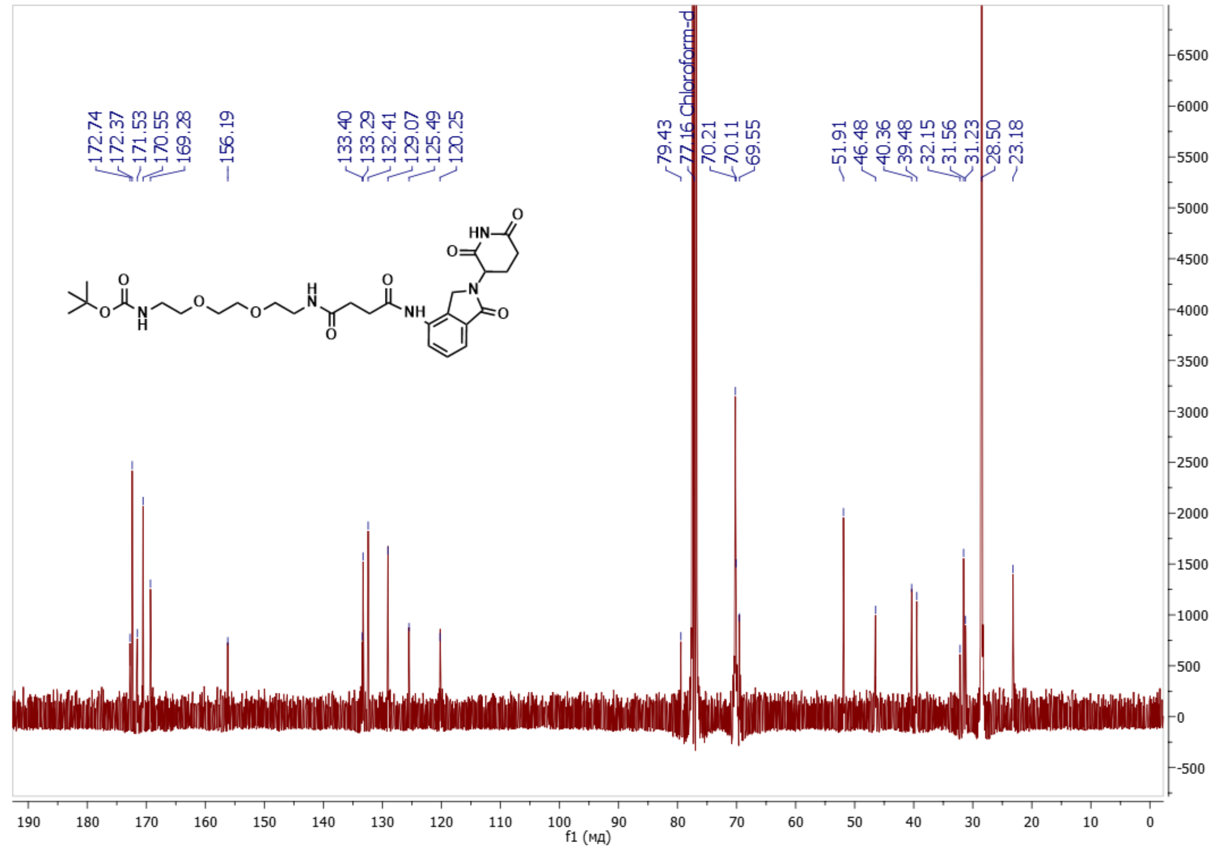

Compound **11b**

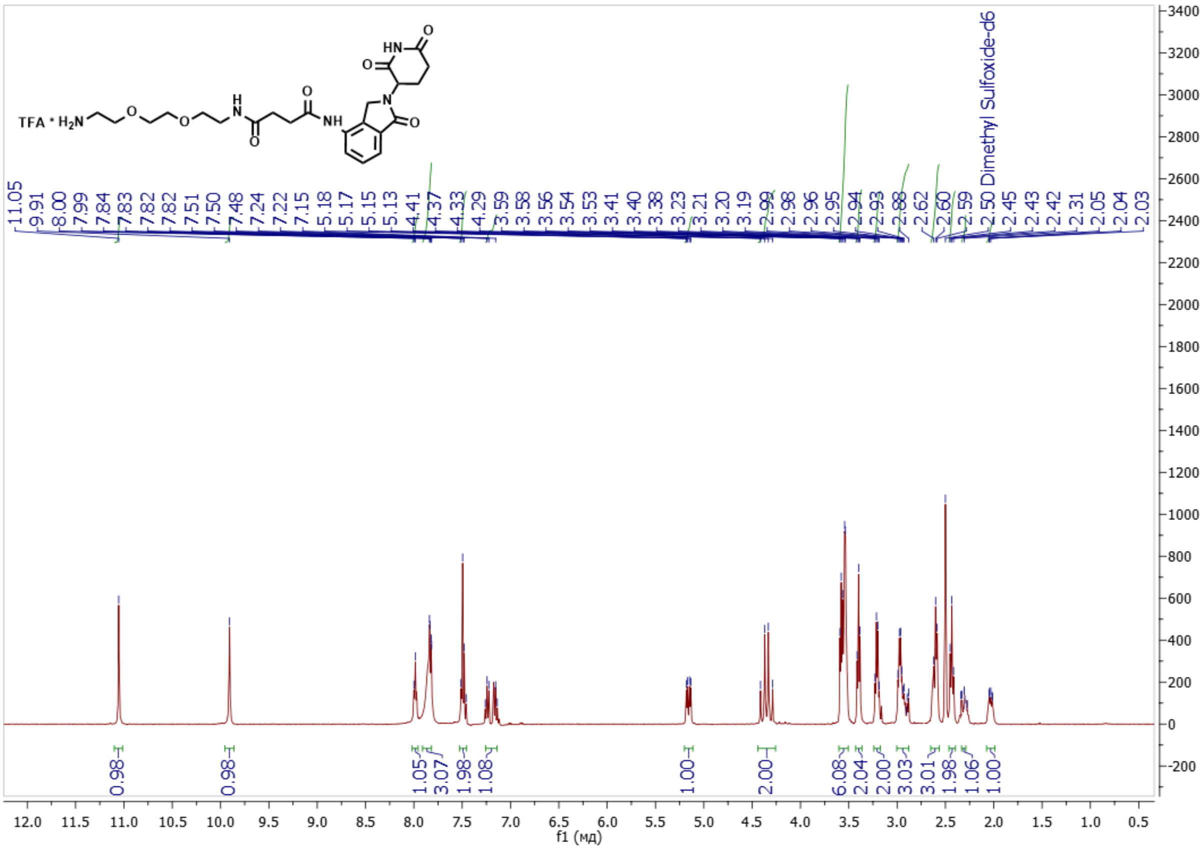


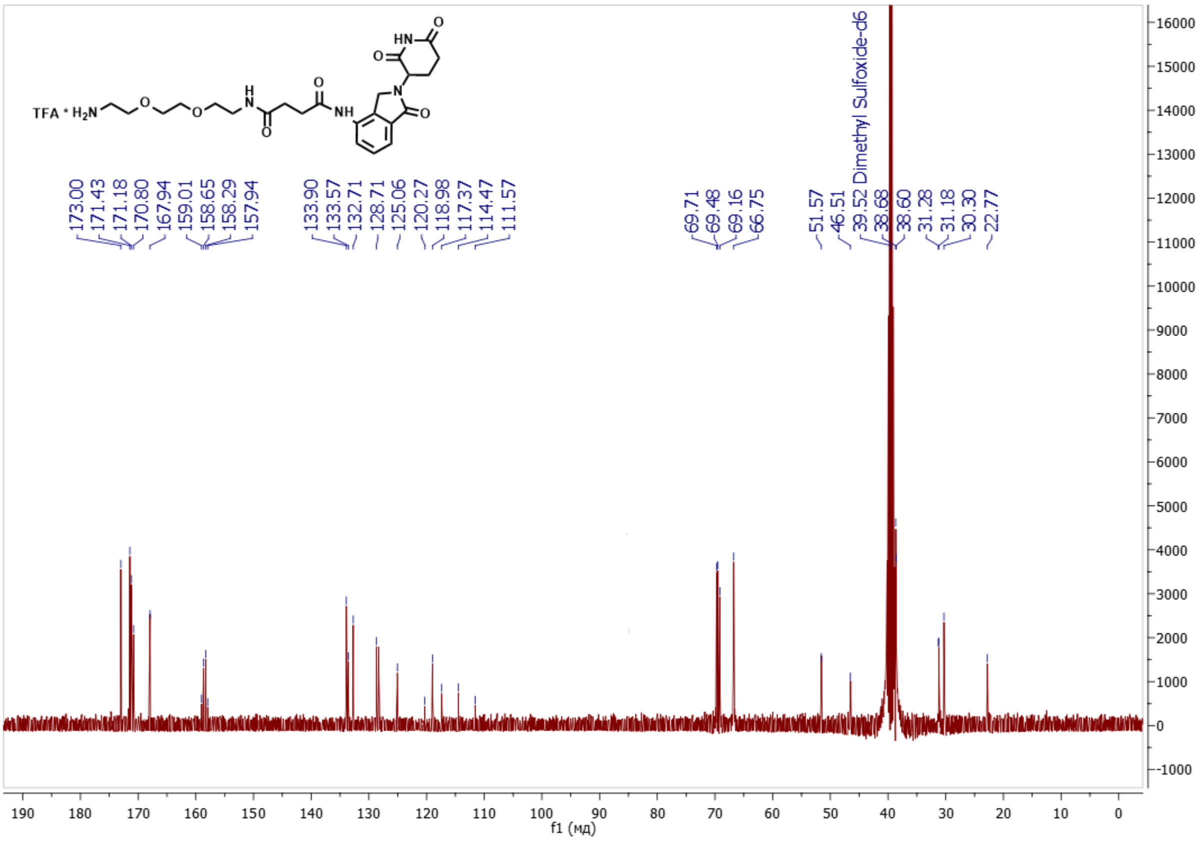

Compound **15a**

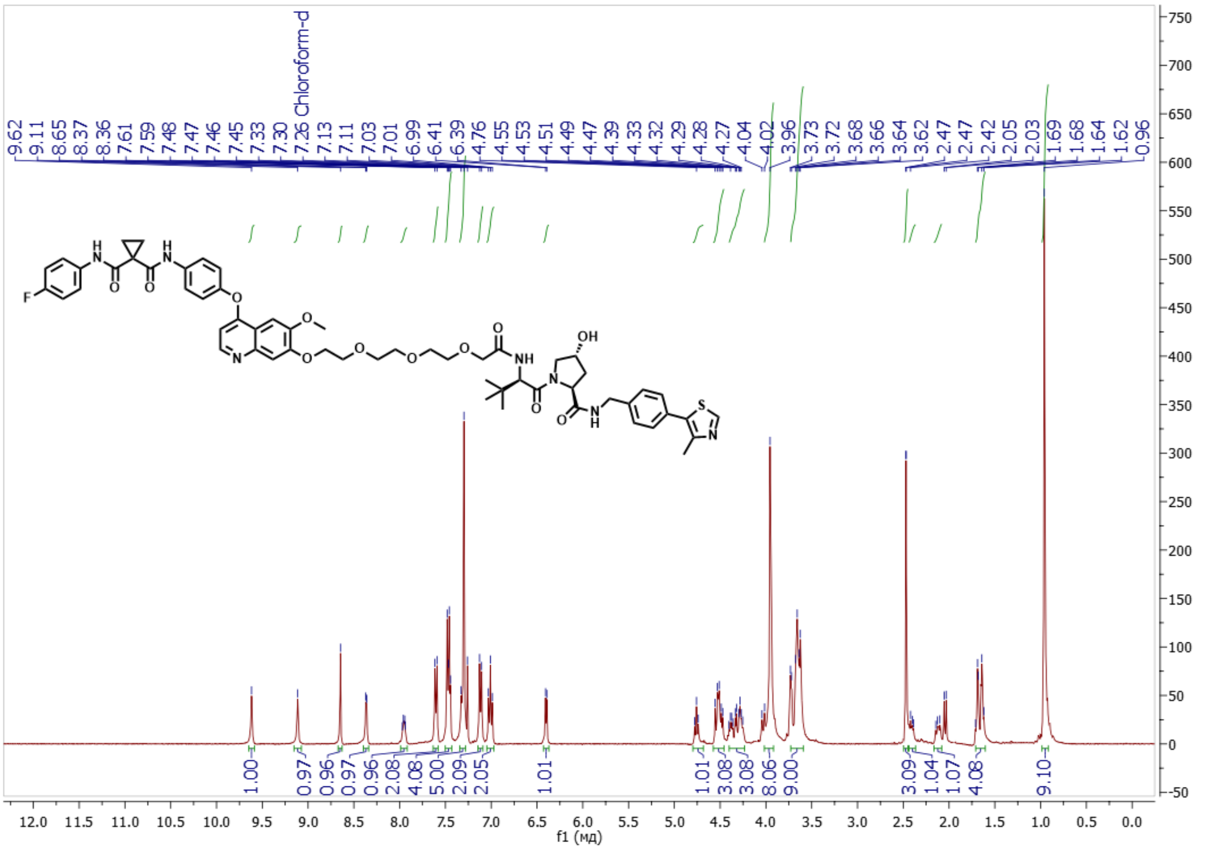

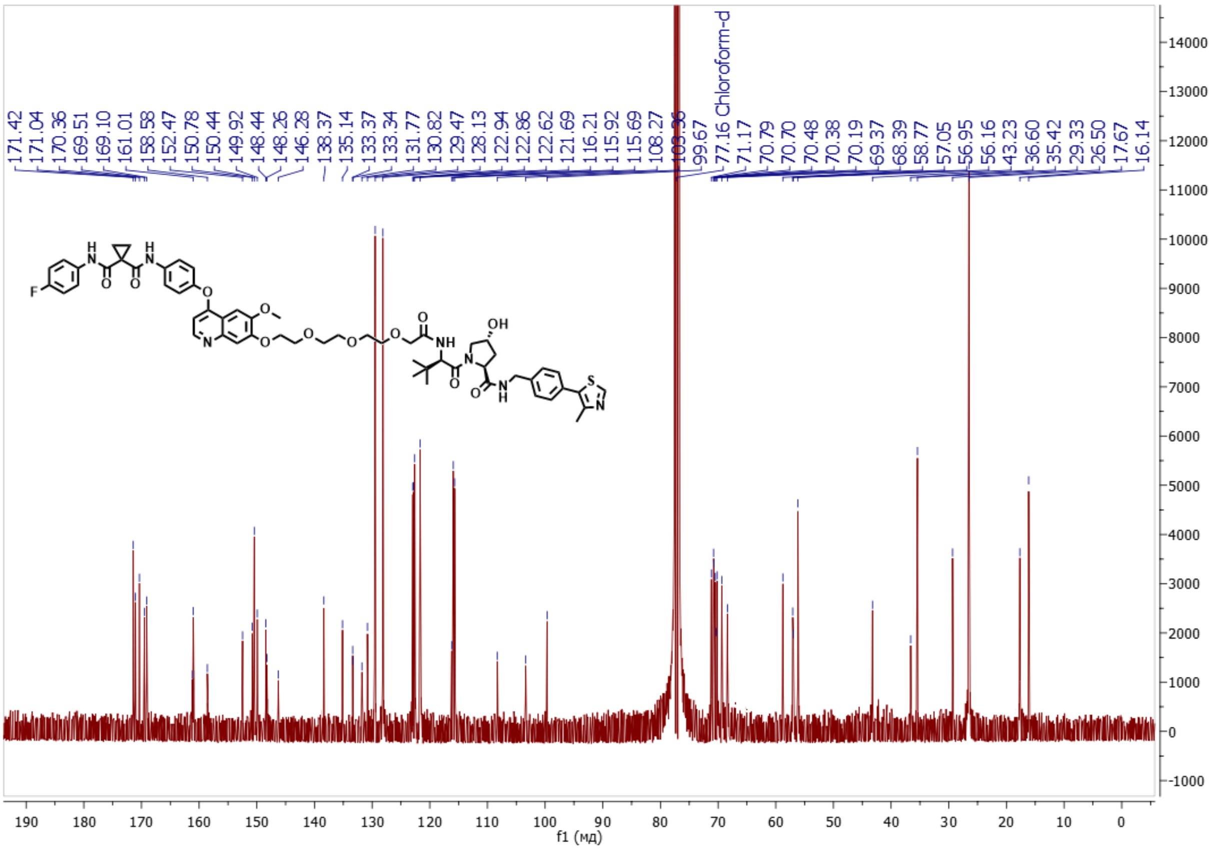

Compound **15b**

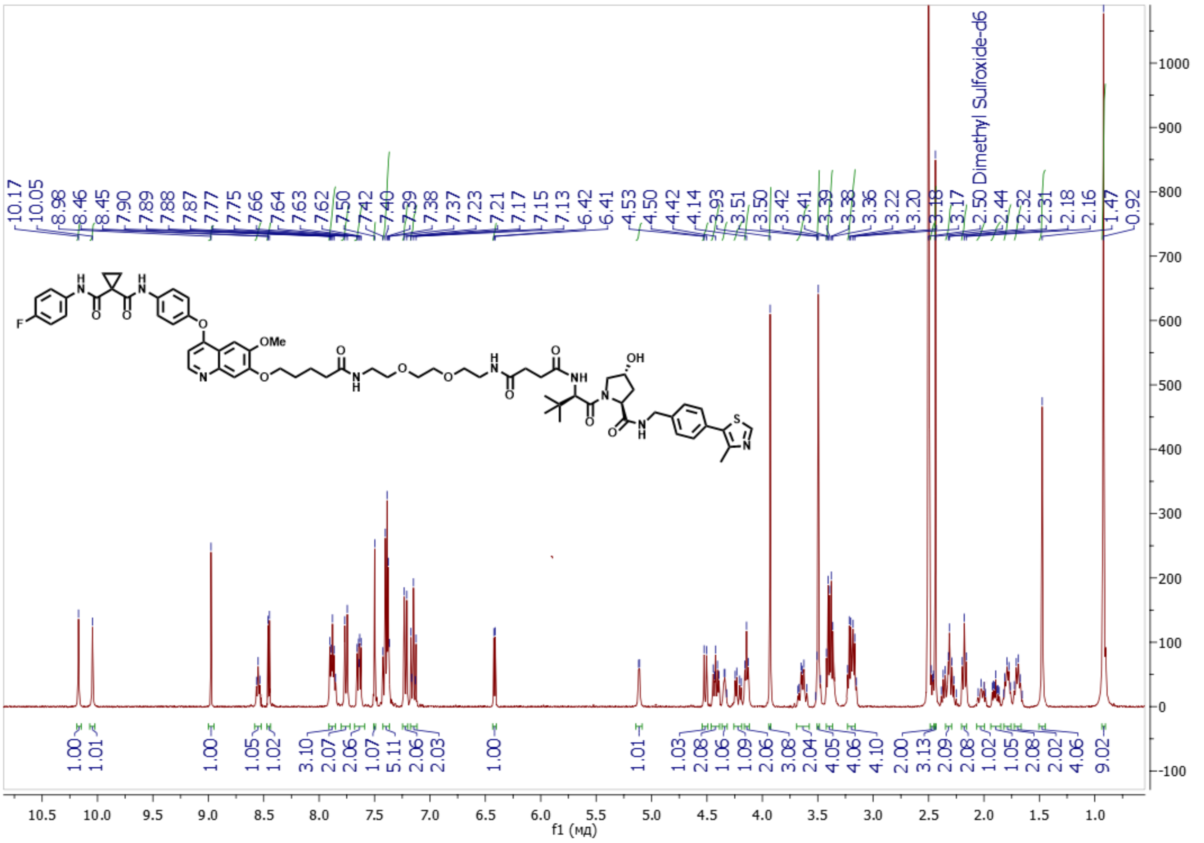

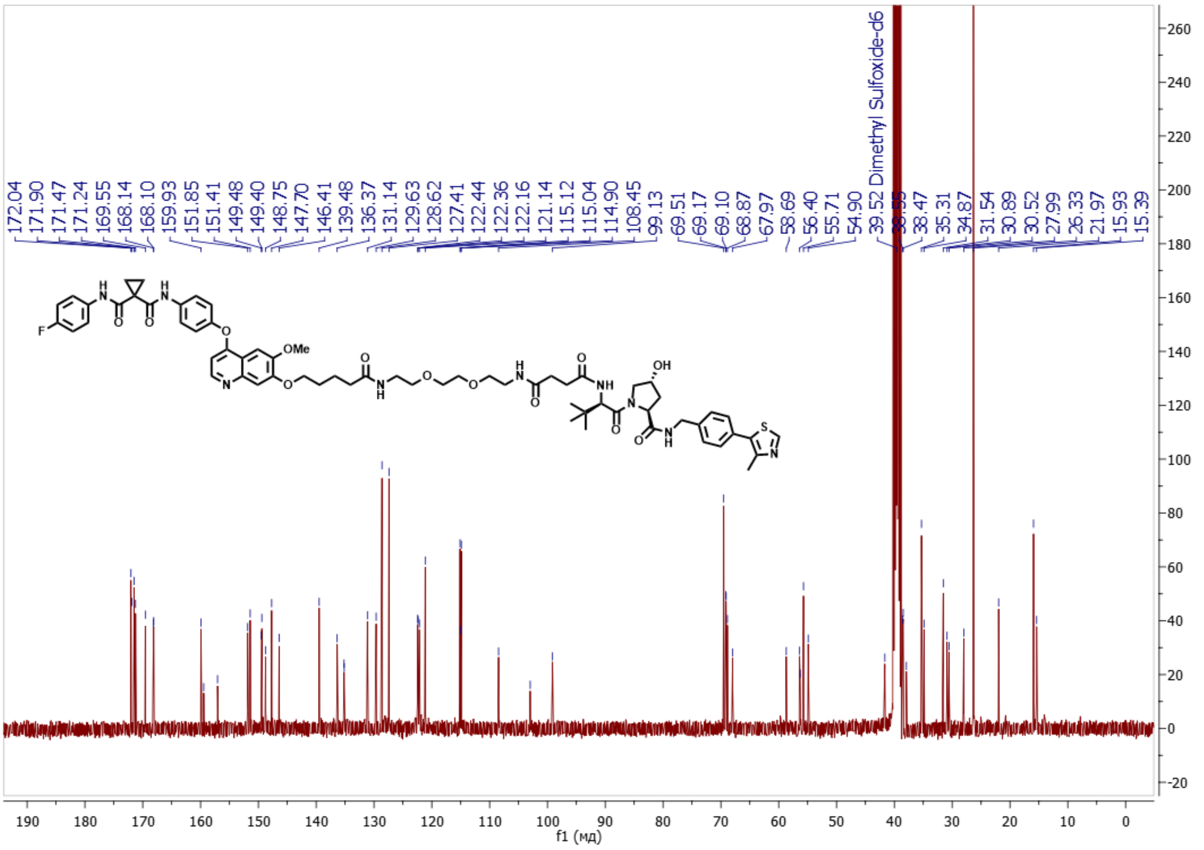

Compound **15c**

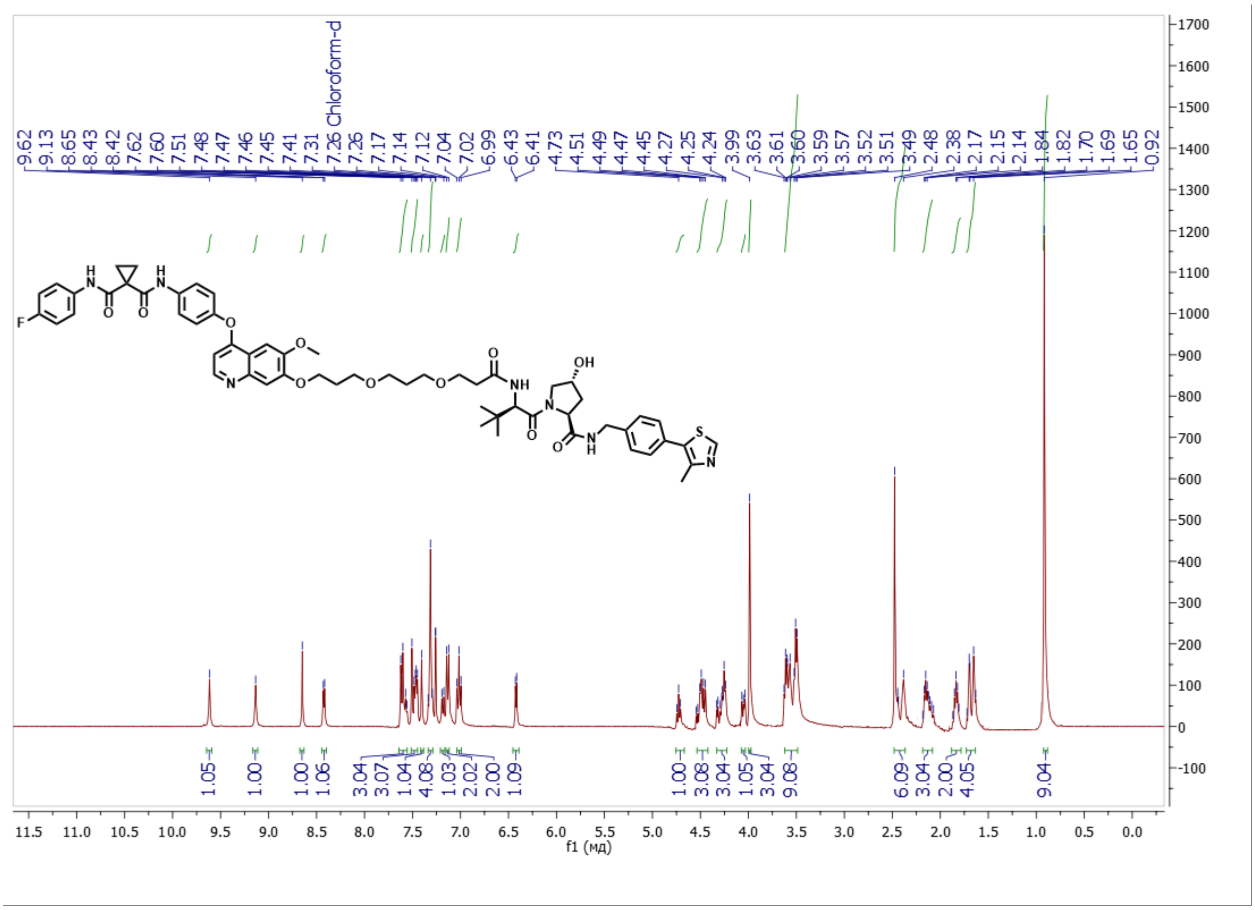

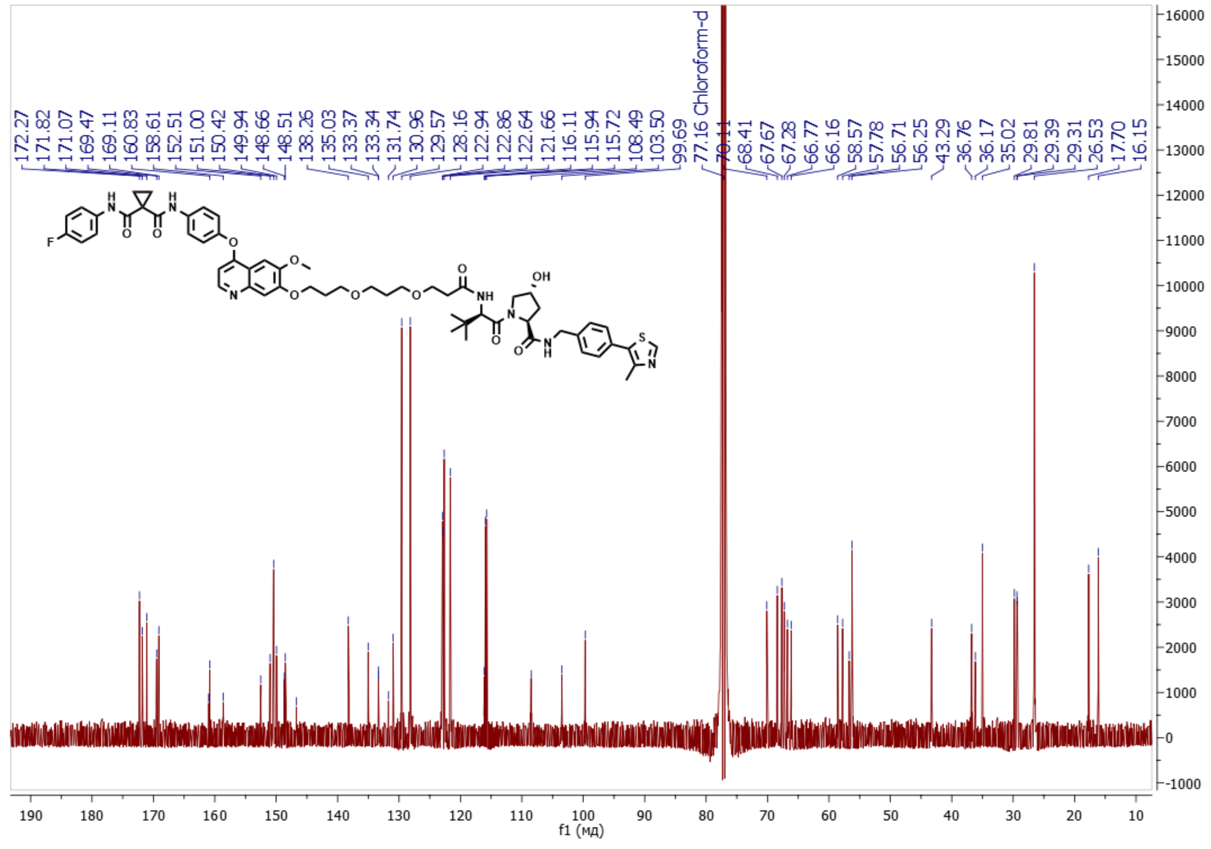

Compound **15d**

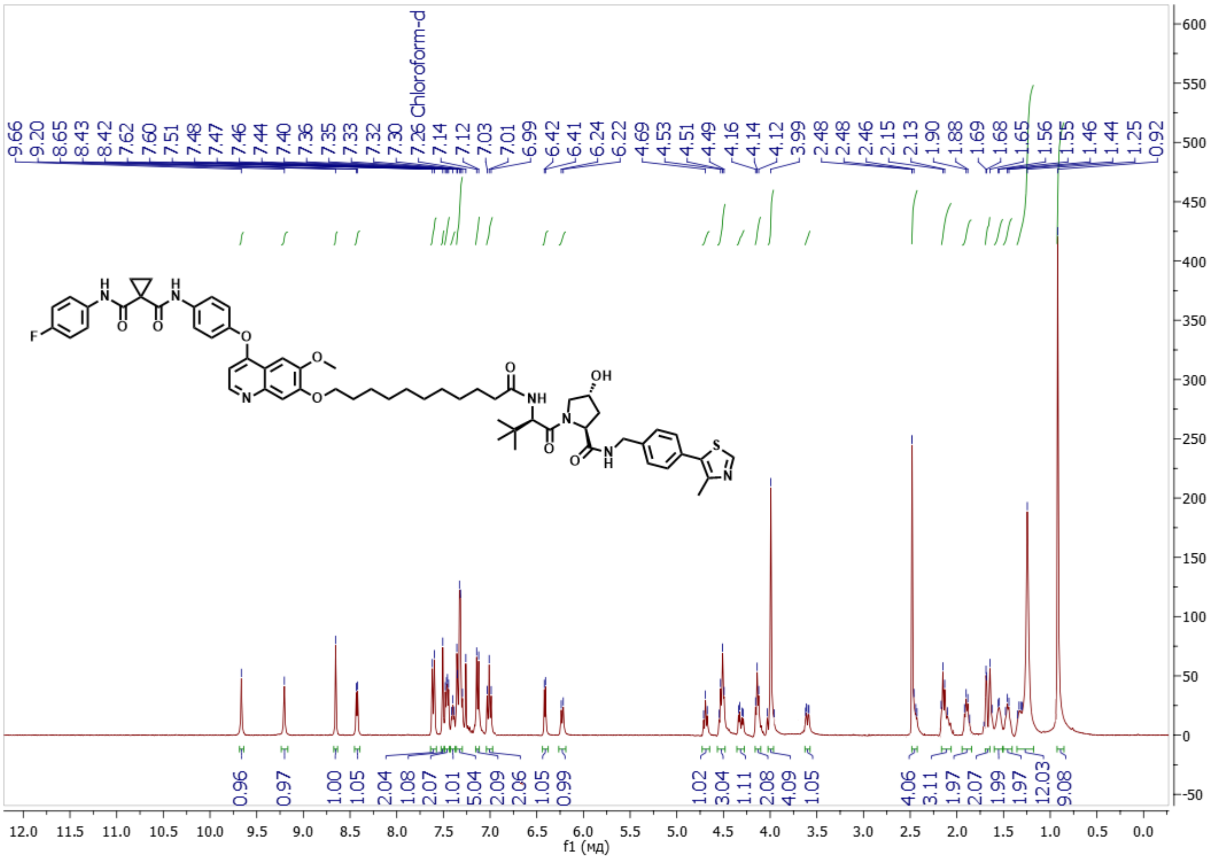


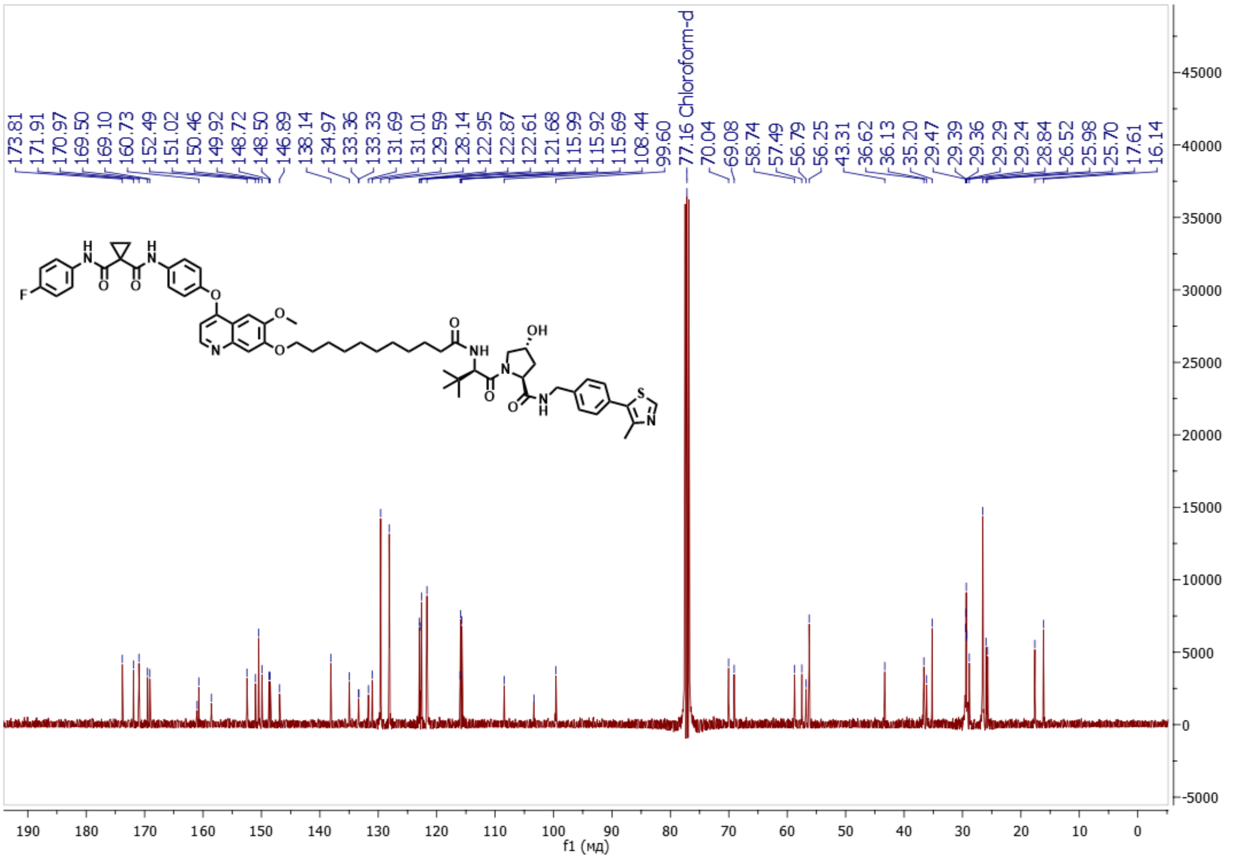

Compound **16a**

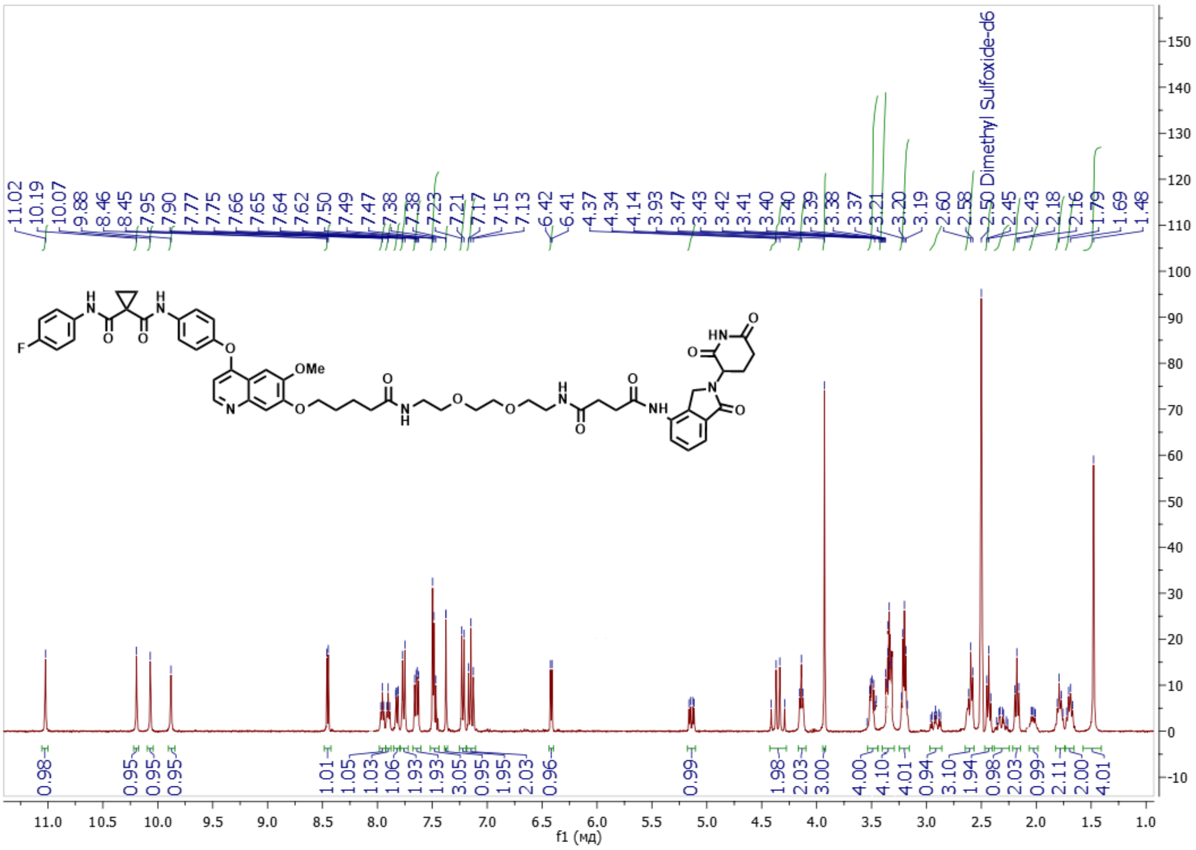


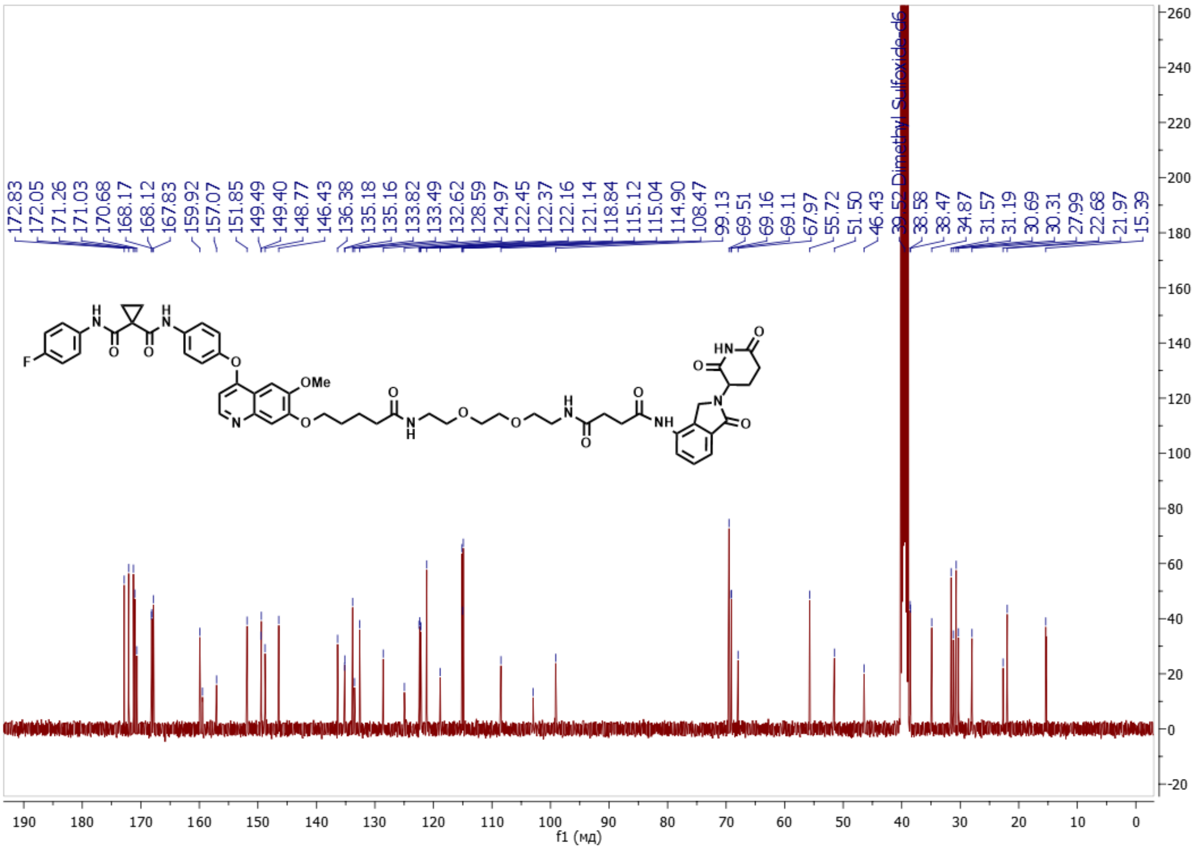

Compound **16b**

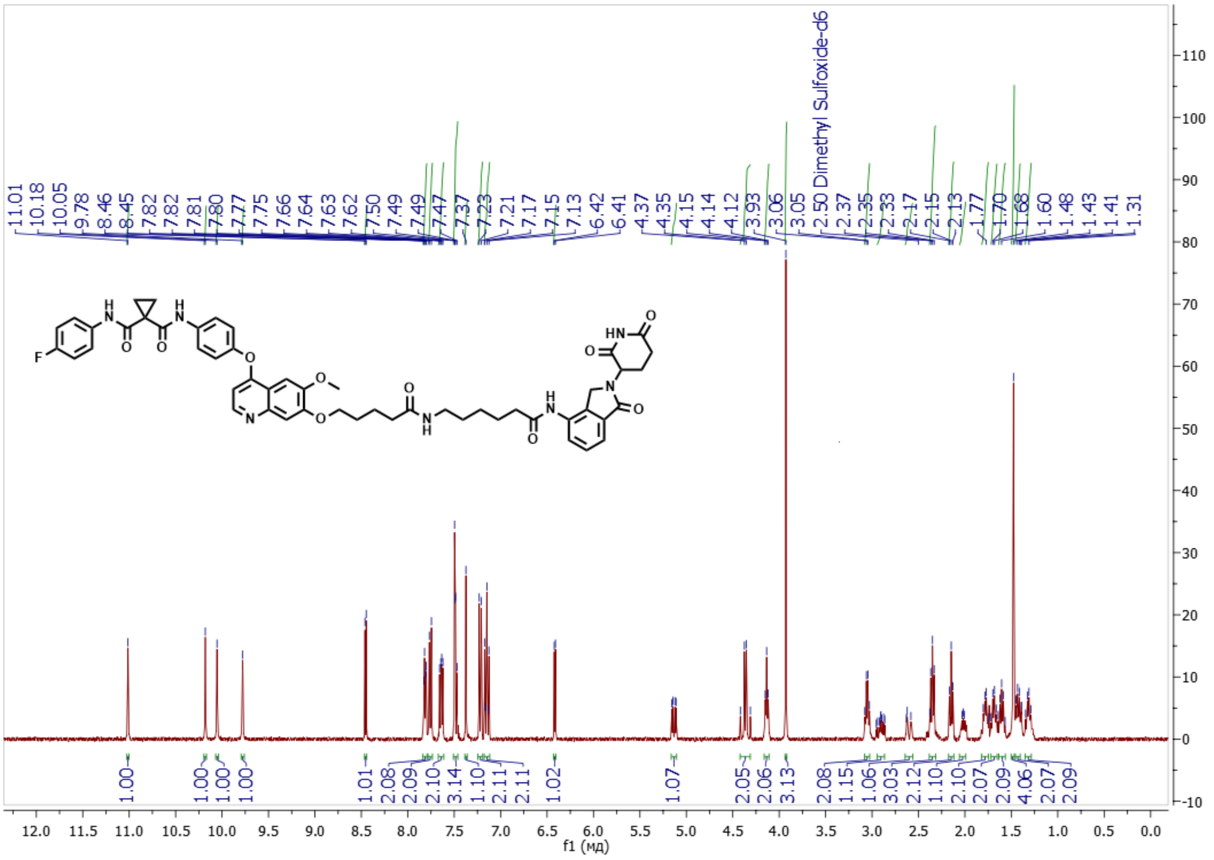


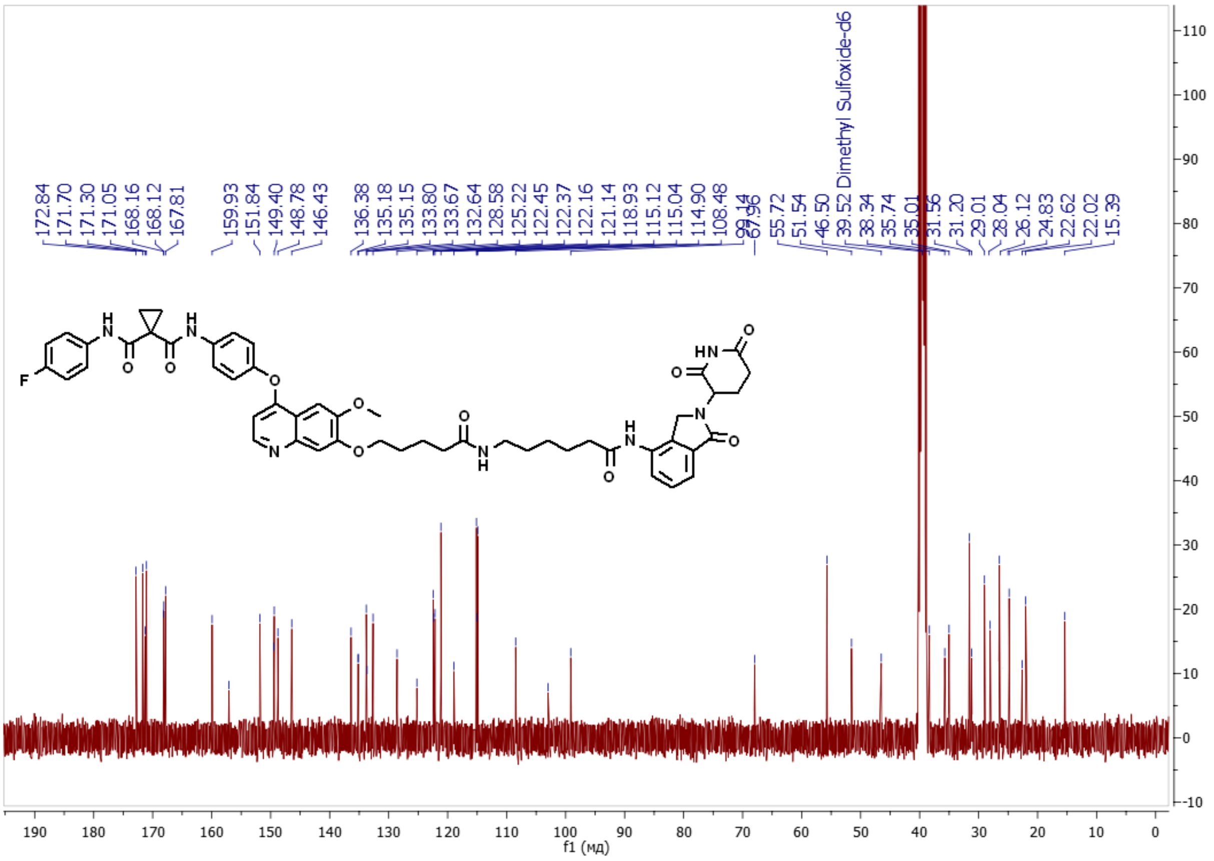

Compound **17a**

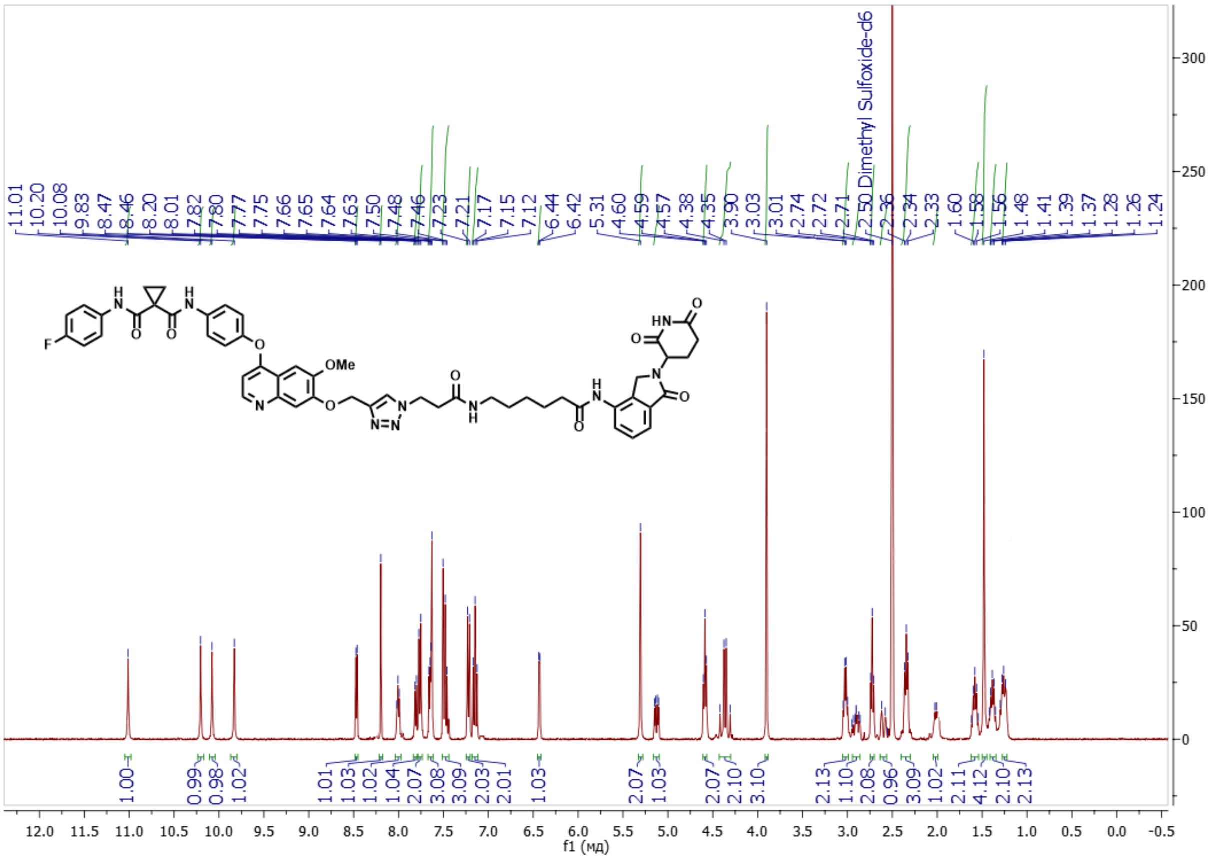

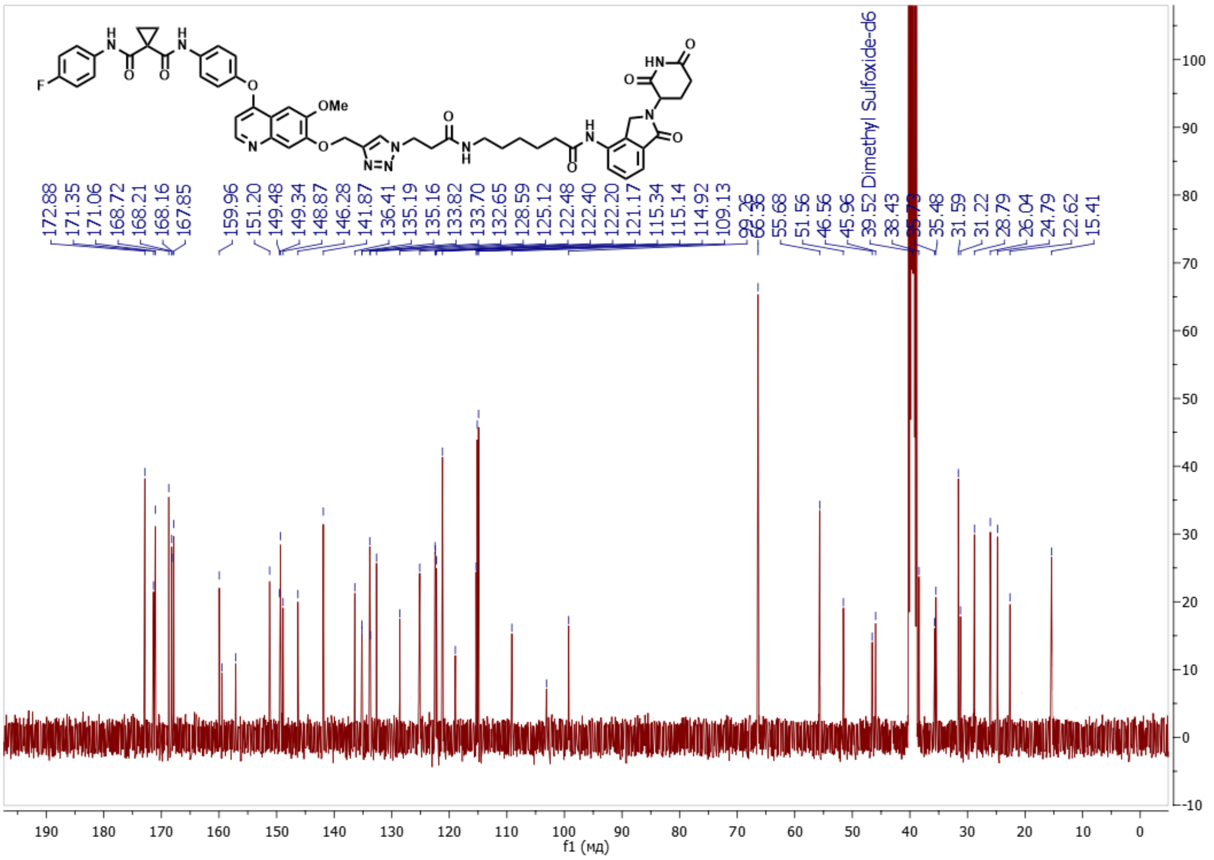

Compound **17b**

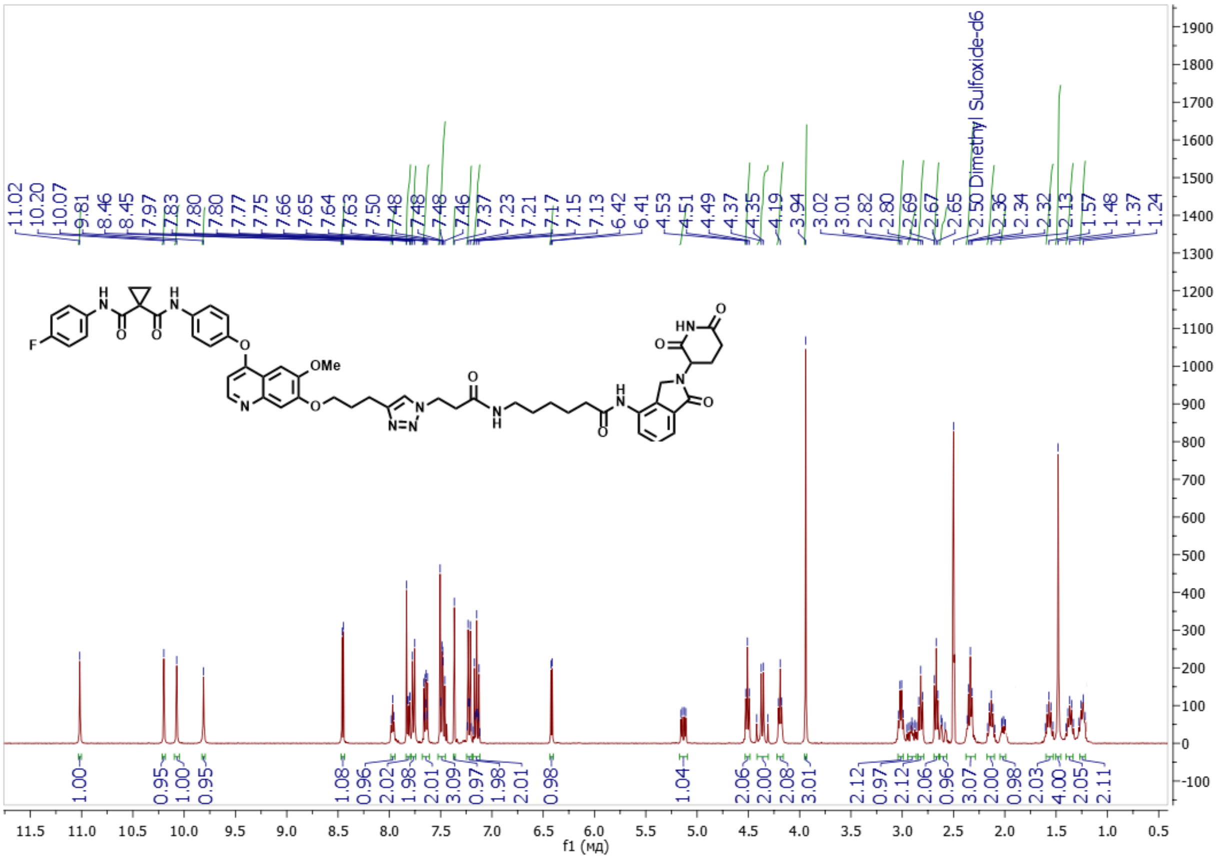

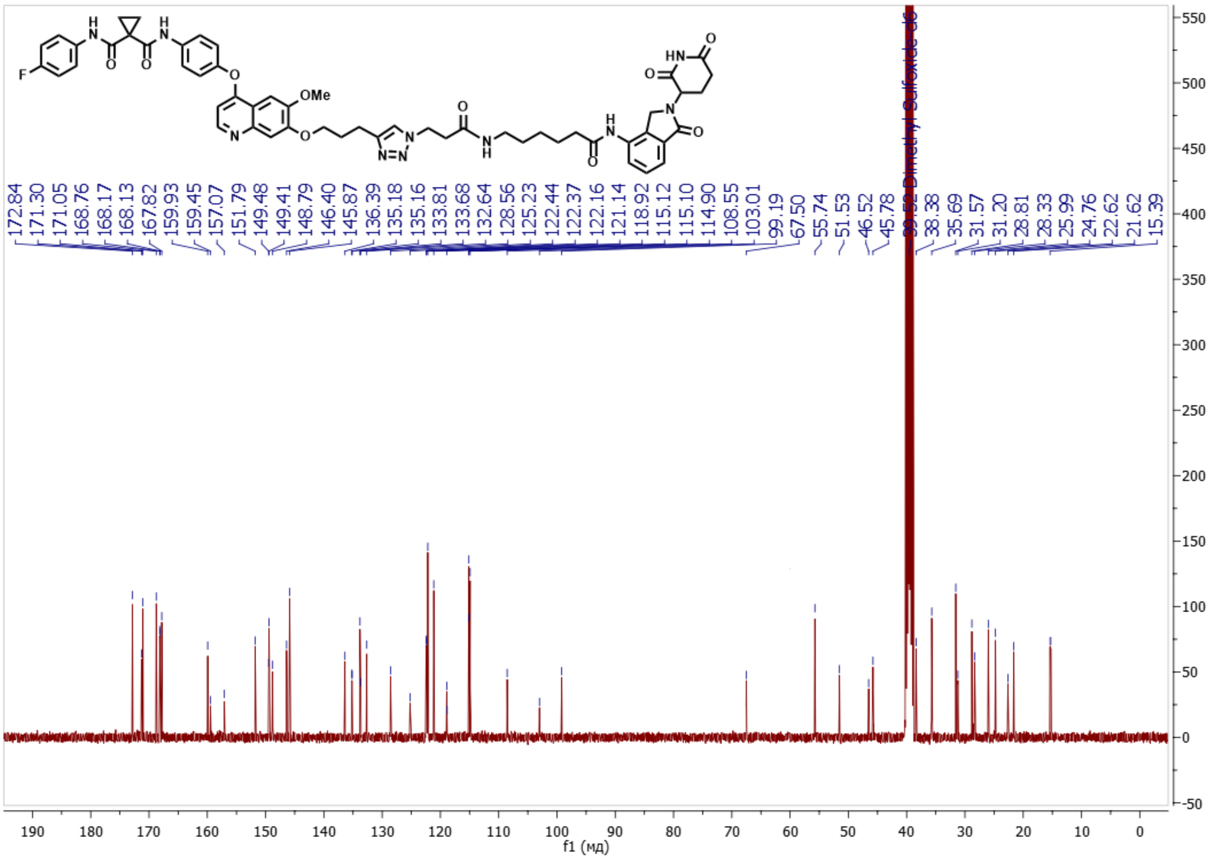

Compound **17c**

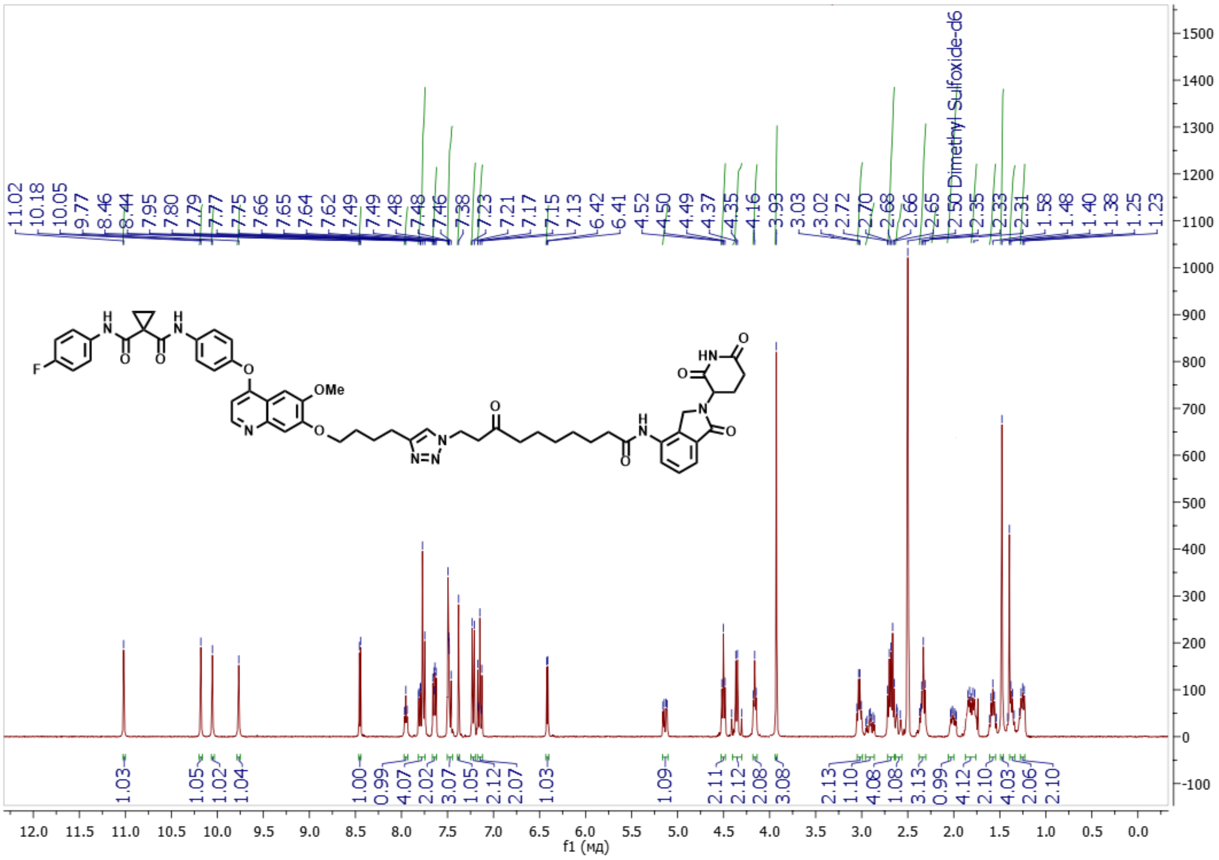

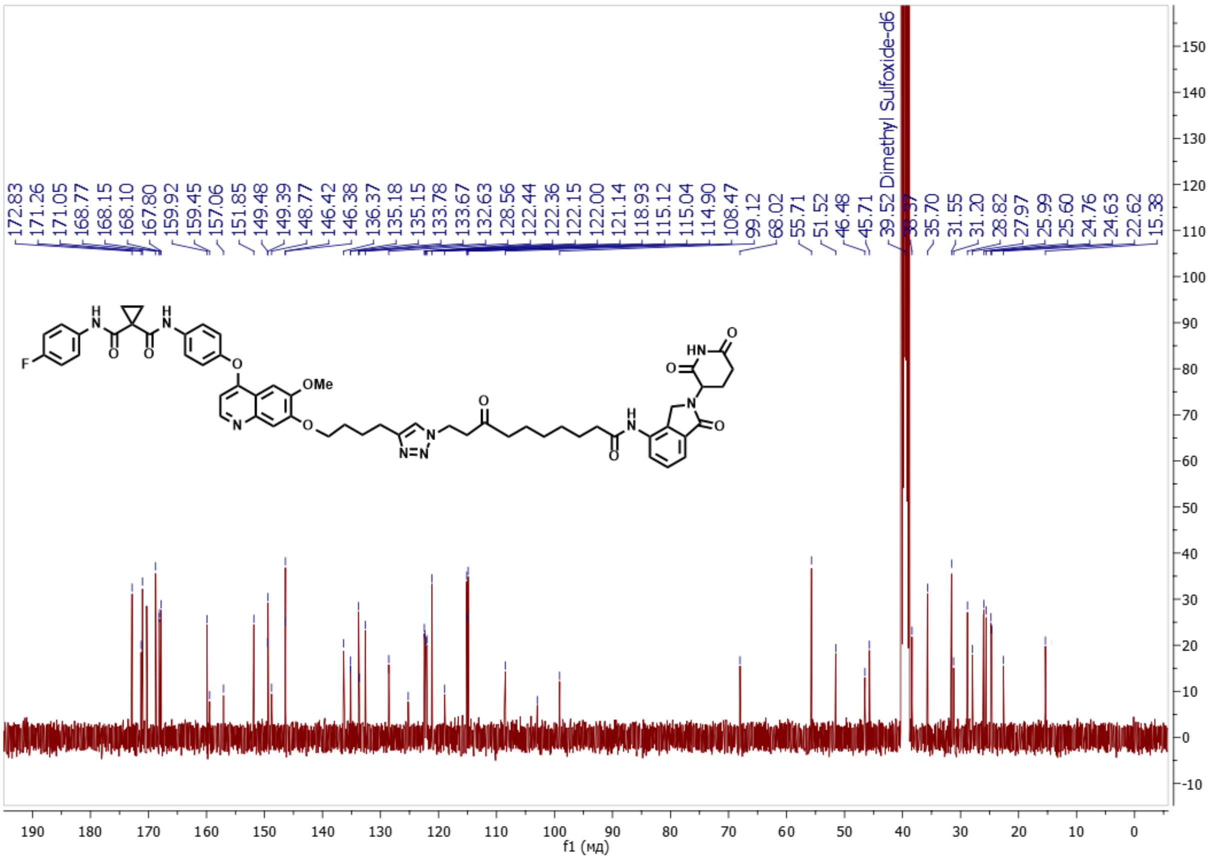

Compound **17d**

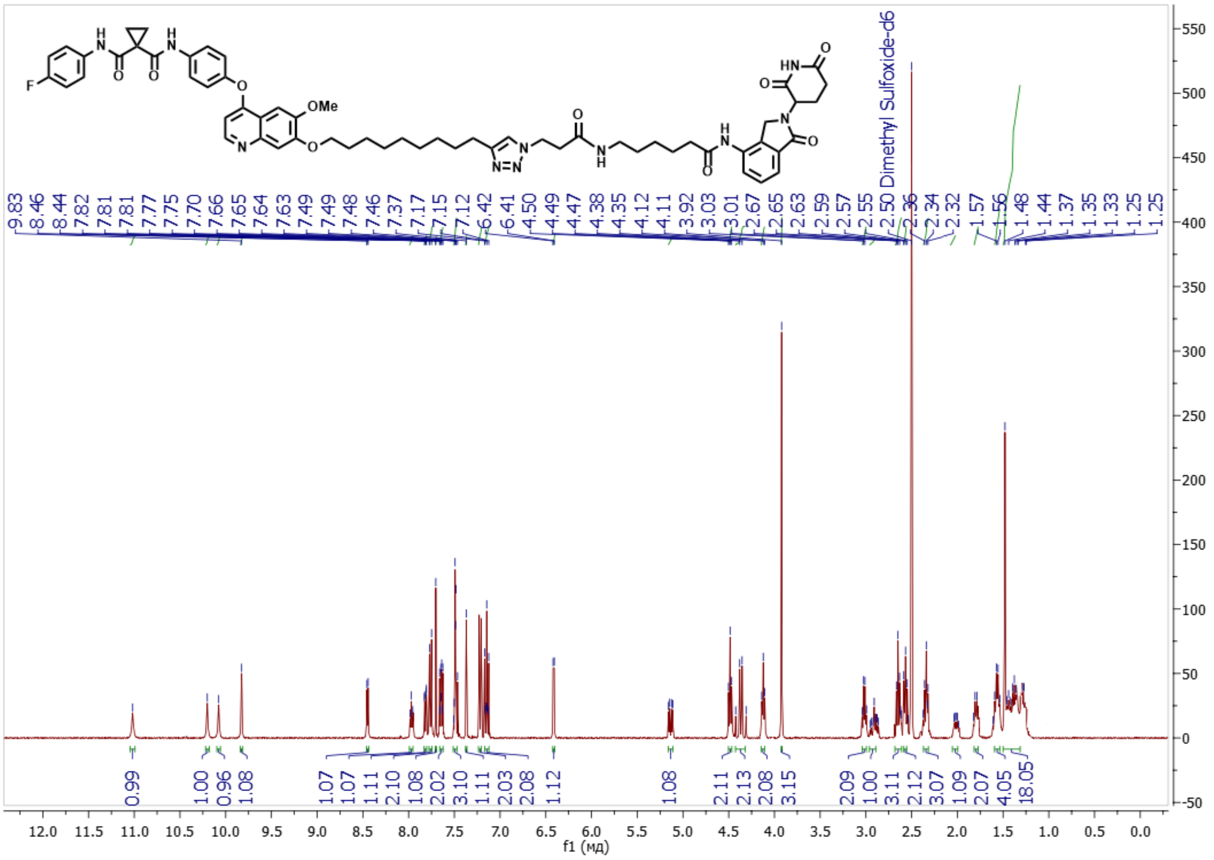

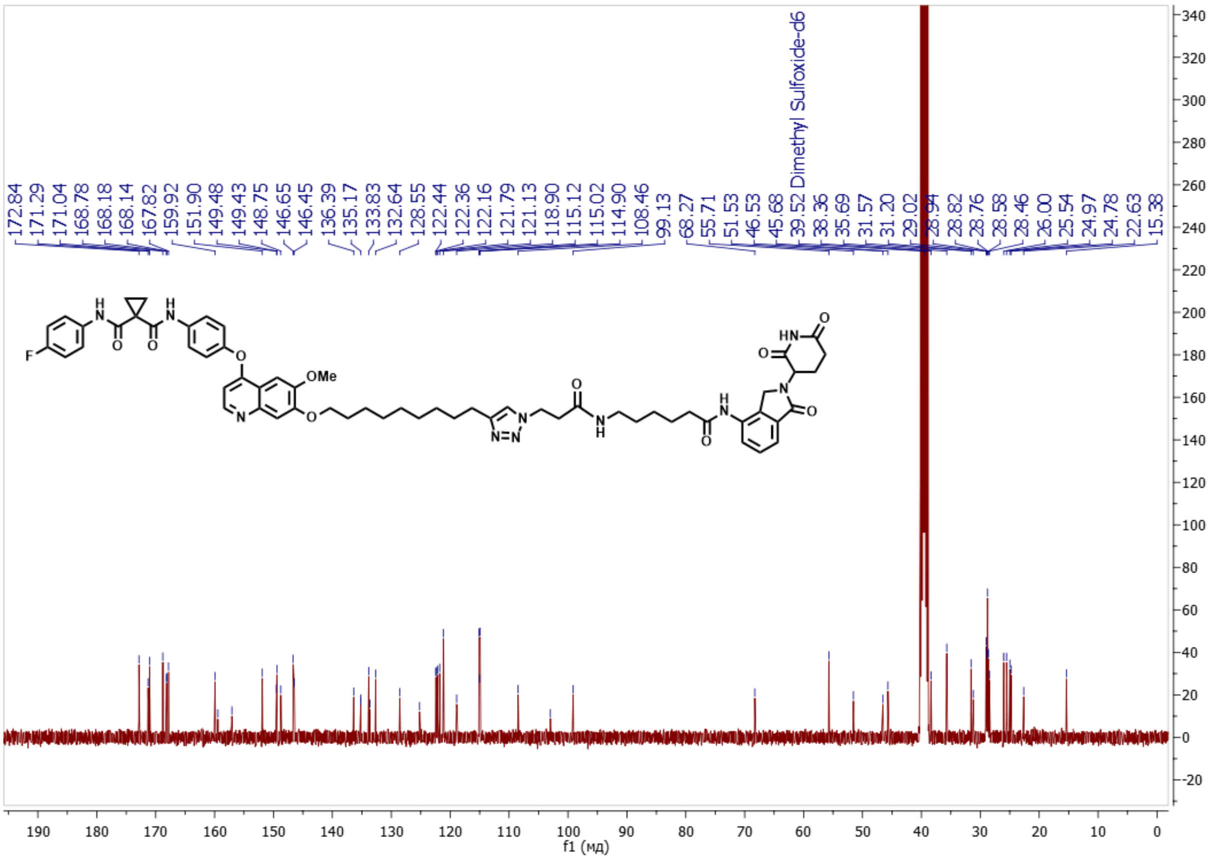

## Antiproliferative activity of target compounds

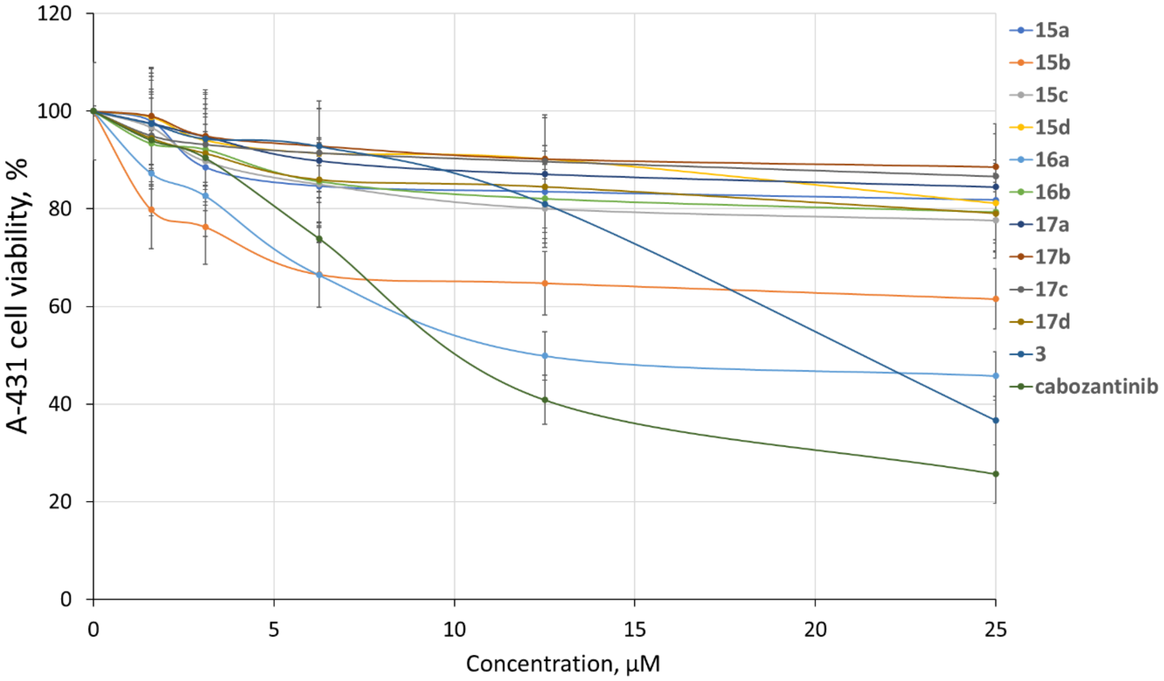


**Figure S1**. Antiproliferative activity of target compounds **15a-d**, **16a**, **b**, **17a-d** against A-431 cells; the A-431 cells were incubated with compounds for 3 days and the cell viability was assessed by the MTT test.

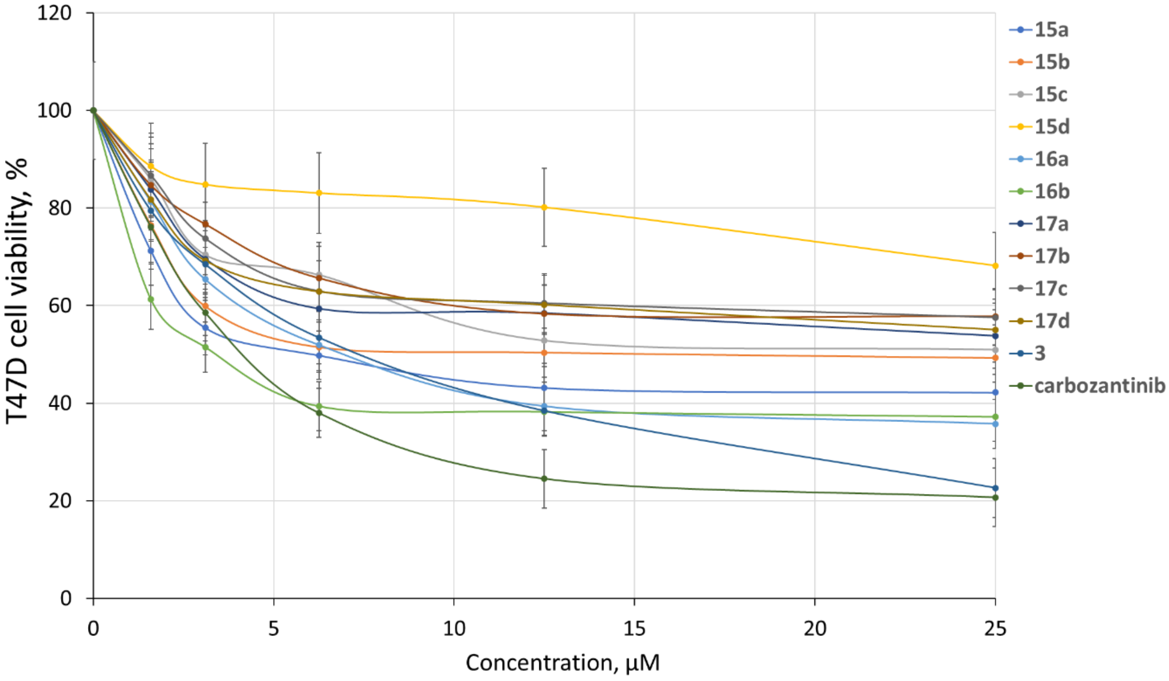


**Figure S2**. Antiproliferative activity of target compounds **15a-d**, **16a**, **b**, **17a-d** against T47D cells.

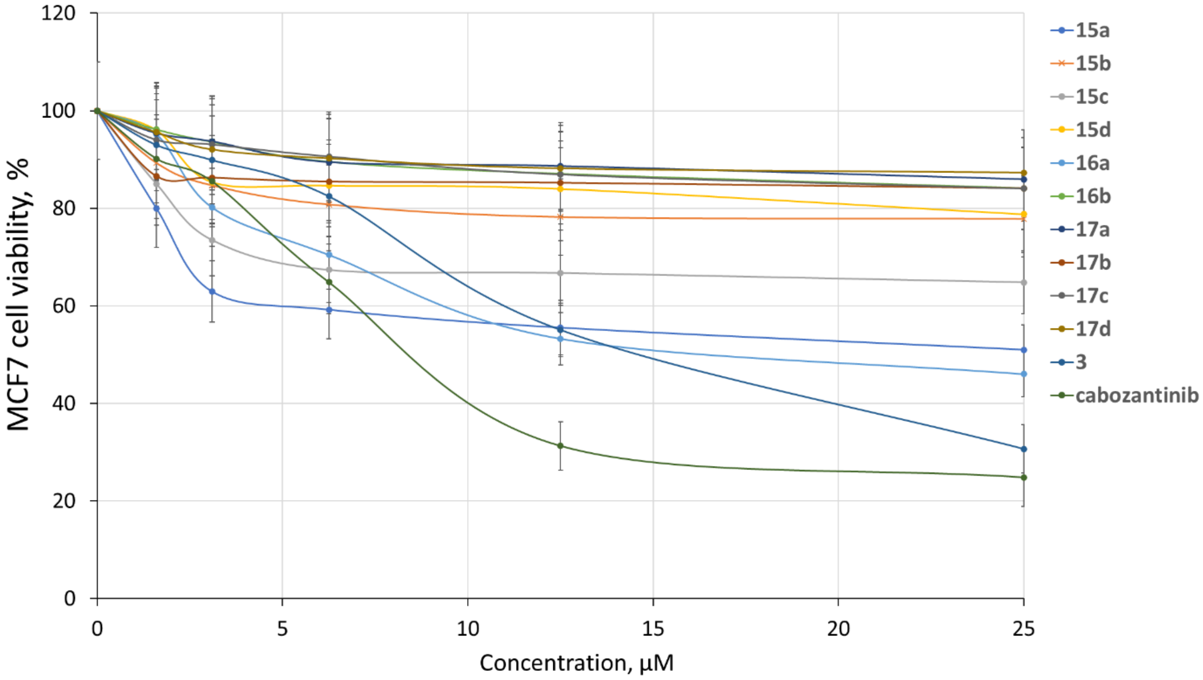


**Figure S3**. Antiproliferative activity of target compounds **15a-d**, **16a**, **b**, **17a-d** against MCF7 cells.

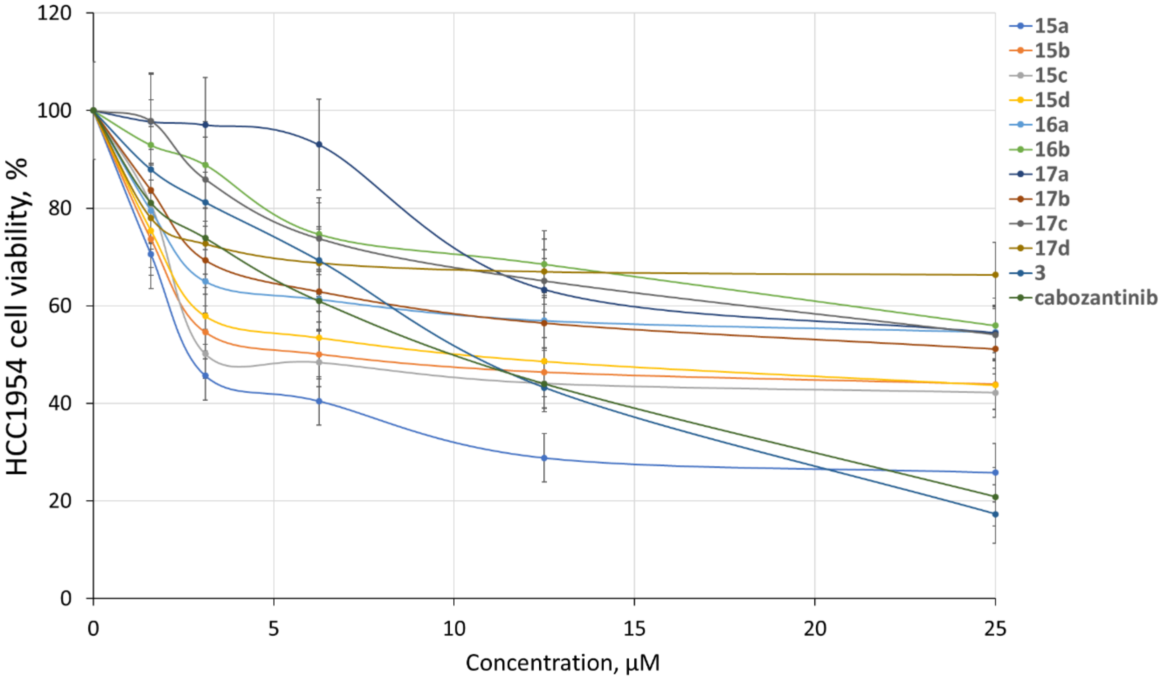


**Figure S4**. Antiproliferative activity of target compounds **15a-d**, **16a**, **b**, **17a-d** against HCC1954 cells.

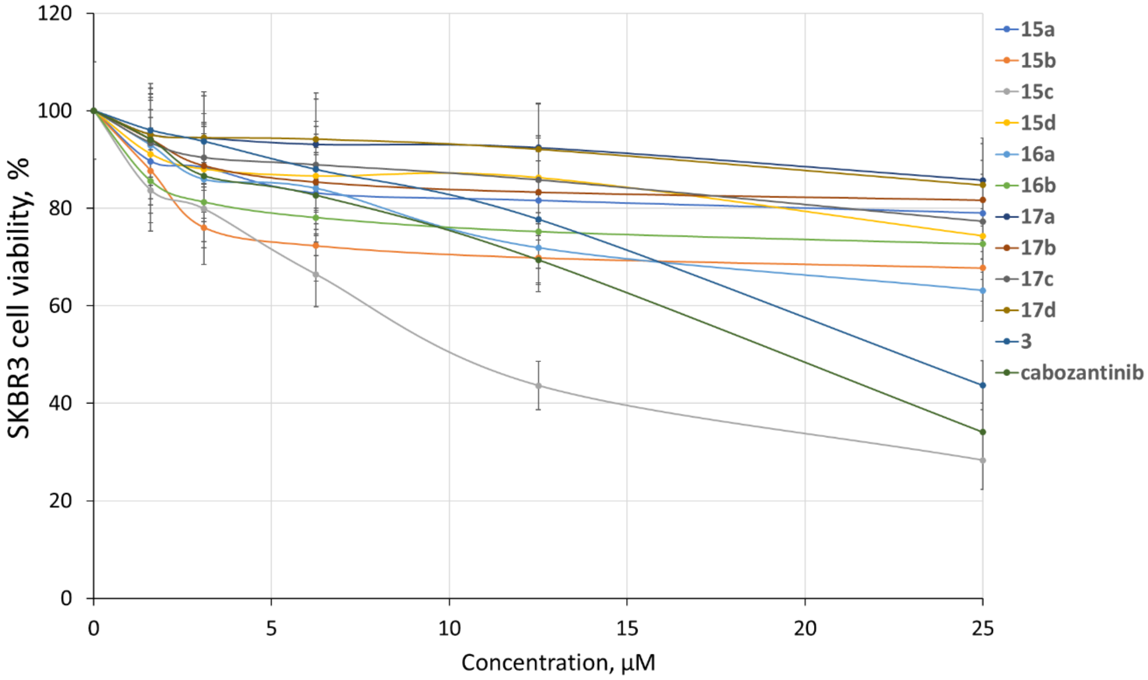


**Figure S5**. Antiproliferative activity of target compounds **15a-d**, **16a**, **b**, **17a-d** against SKBR3 cells.

## References

1. Steinebach, C., Kehm, H., Lindner, S., Vu, L.P., Köpff, S., López Mármol, Á., Weiler, C., Wagner, K.G., Reichenzeller, M., Krönke, J., and Gütschow, M. (2019) PROTAC-mediated crosstalk between E3 ligases. *Chem. Commun. (Camb). 55 (12),* 1821-1824.
2. Zheng, G., Zhou, D., Zhang, X., Wang, Y., and Chang J. WO2017184995 USA.
3. Govind Gore, V., Kumar Shukla, V., Shrikant Bhandari, S., and Hasbe, S. US8946265B2 USA.
4. Zhang, F., Wu, Z., Chen, P., Zhang, J., Wang, T., Zhou, and J., Zhang, H. (2020) Discovery of a new class of PROTAC BRD4 degraders based on a dihydroquinazolinone derivative and lenalidomide/pomalidomide. *Bioorg. Med. Chem. 28 (1)*, 115228.
5. Bannen, L.C, Chan, D.S-M., Forsyth, T.P., Khoury, R.G., Leahy, J.W., Mac-Mann, M.B., L.W., Nuss, J.M., Parks, J. J., Wang, Y., and Xu, W. US7579473B2 USA.
